# Harnessing the electronic structure of active metals to lower the overpotential of the electrocatalytic oxygen evolution reaction†

**DOI:** 10.1039/d3sc05891c

**Published:** 2023-12-12

**Authors:** Lorenzo Baldinelli, Gabriel Menendez Rodriguez, Iolanda D'Ambrosio, Amalia Malina Grigoras, Riccardo Vivani, Loredana Latterini, Alceo Macchioni, Filippo De Angelis, Giovanni Bistoni

**Affiliations:** a Dipartmento di Chimica, Biologia e Biotecnologie, Università Degli Studi Di Perugia Via Elce di sotto, 8 06123 Perugia Italy gabriel.menendezrodriguez@unipg.it giovanni.bistoni@unipg.it; b Dipartimento di Scienze Farmaceutiche, Università Degli Studi Di Perugia Via del Liceo 06123 Perugia Italy; c Computational Laboratory for Hybrid/Organic Photovoltaics (CLHYO), Istituto CNR di Scienze e Tecnologie Chimiche “Giulio Natta” (CNR-SCITEC) 06123 Perugia Italy; d Department of Mechanical Engineering, College of Engineering, Prince Mohammad Bin Fahd University Al Khobar 31952 Saudi Arabia; e SKKU Institute of Energy Science and Technology (SIEST), Sungkyunkwan University Suwon 440-746 Korea

## Abstract

Despite substantial advancements in the field of the electrocatalytic oxygen evolution reaction (OER), the efficiency of earth-abundant electrocatalysts remains far from ideal. The difficulty stems from the complex nature of the catalytic system, which limits our fundamental understanding of the process and thus the possibility of a rational improvement of performance. Herein, we shed light on the role played by the tunable 3d configuration of the metal centers in determining the OER catalytic activity by combining electrochemical and spectroscopic measurements with an experimentally validated computational protocol. One-dimensional coordination polymers based on Fe, Co and Ni held together by an oxonato linker were selected as a case study because of their well-defined electronic and geometric structure in the active site, which can be straightforwardly correlated with their catalytic activity. Novel heterobimetallic coordination polymers were also considered, in order to shed light on the cooperativity effects of different metals. Our results demonstrate the fundamental importance of electronic structure effects such as metal spin and oxidation state evolutions along the reaction profile to modulate ligand binding energies and increase catalyst efficiency. We demonstrated that these effects could in principle be exploited to reduce the overpotential of the electrocatalytic OER below its theoretical limit, and we provide basic principles for the development of coordination polymers with a tailored electronic structure and activity.

## Abstract 2

An integrated computational and experimental strategy is used to shed light on the mechanism of the OER on coordination polymers of earth abundant metals, revealing the crucial influence of spin and oxidation state changes on the overpotential.

## Graphical abstract

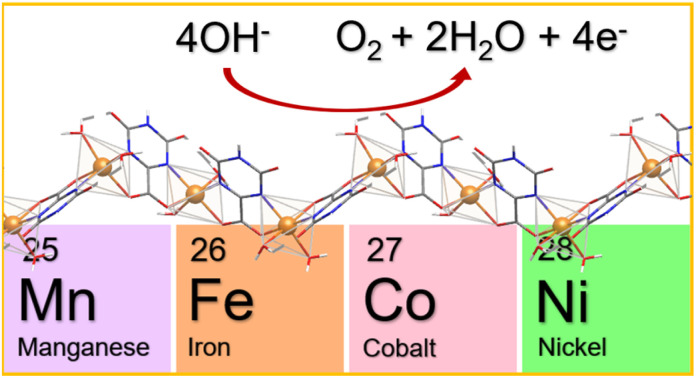

## 1. Introduction

Water electrolysis powered by renewable energies has attracted increasing interest in recent years as a sustainable source of molecular hydrogen, which is one of the most promising alternatives to fossil fuels as an energy carrier.^1,2^ This process is composed of two half-reactions, namely the oxygen evolution reaction (OER) at the anode and the hydrogen evolution reaction (HER) at the cathode. The sluggish four electron transfer processes involved in the OER mechanism limit the overall hydrogen production efficiency, leading to high overpotentials.^3^ This stimulated intense research efforts towards the development of low-cost, efficient and stable OER-electrocatalysts.^4–8^

Under acidic conditions, precious metal-based oxides such as IrO_2_ and RuO_2_ can be considered as the state-of-the-art OER electrocatalysts because of their remarkable activity and stability;^9–12^ however, their large-scale application is hindered by the high cost and low abundance of the metals. Consequently, an intense research activity aimed at minimizing the amount of noble-metal utilization is ongoing.^13–19^

Non-precious metal-oxides and hydroxides under oxidative potentials are only stable under alkaline conditions,^20^ and hence great efforts have been made to develop earth-abundant transition-metal-based OER-electrocatalysts with sustained stability in alkaline solution.^21–25^ Nevertheless, despite substantial advancements in the field, the efficiency and stability of such OER catalysts remain far from ideal. In particular, there seems to be a lower bound for the overpotential that can be obtained, which is approximately 0.2–0.4 V. This effect has been attributed to linear scaling relationships between OOH and OH binding energies limiting the OER activity.^26,27^

A major difficulty in the development of new strategies for breaking such linear scaling relationships is the lack of fundamental knowledge on the electronic and geometric nature of the active site for many heterogeneous catalysts.^28–32^ The difficulty stems from the complex nature of the OER catalytic materials and of their interaction with the environment. On the one hand, such complexity limits the chemical insights that can be obtained from purely experimental investigations.^33^ This is especially evident for metal oxides, for which the geometric and electronic nature of the active site is still a matter of debate,^34,35^ making rational design of new catalysts particularly challenging. On the other hand, the strong dependence of the reaction free energies on the exchange-correlation functional choice together with the limited accuracy of standard computational methodologies in the calculation of redox potentials, acidity constants and reaction energies in water limits the confidence on purely computational predictions.^33,36–39^ Hence, synergistic computational and experimental studies are crucial to elucidate the mechanistic underpinnings responsible for the catalyst activity and stability, and for deriving the rule of thumbs for new materials with lower overpotentials.

Although the integration of experimental and theoretical studies has already proven instrumental in this context,^40^ fundamental questions on the origin of the catalytic activity of many materials still remain unanswered.^41^ In particular, an in-depth understanding of the relationship between a material catalytic activity and its electronic structure is often lacking. For example, it is well known that the metal spin state has a deep influence on the OER efficiency.^42–44^ However, the spin state of metals can change in the course of an electrochemical reaction^45^ or in response to external stimuli such as an external potential.^46^ Therefore, it is somewhat surprising that spin crossover effects in the heterogeneous electrocatalytic OER have not been investigated in detail. As a notable exception, Hegner and coworkers recently suggested on the basis of density functional theory (DFT) calculations that such spin-crossover effects might significantly lower the OER overpotential of a co-hexacyanoferrate electrocatalyst.^47^ Understanding to what extent this effect could be exploited in practical applications, *e.g.*, to break the above-mentioned linear scaling relationships, would be an important step towards rational design.

In this work, our aim is to provide a fundamental insight into the electronic effects that influence a catalyst activity towards the OER using a combined experimental and computational strategy. Among the numerous families of OER catalysts studied and optimized over the years, we selected coordination polymers (CPs)^48–52^ as a prototype case study. These systems are crystalline solids with well-defined periodic structures extending in 1, 2, or 3 dimensions, in which metal “nodes” (clusters or ions) are linked by organic and/or inorganic ligands through coordination bonds. Hence, these materials can be seen as heterogeneous assemblies of transition metal complexes, featuring active sites with well-defined structural and electronic properties, which can be readily correlated with their electrocatalytic activity.^53,54^

**Scheme 1:**
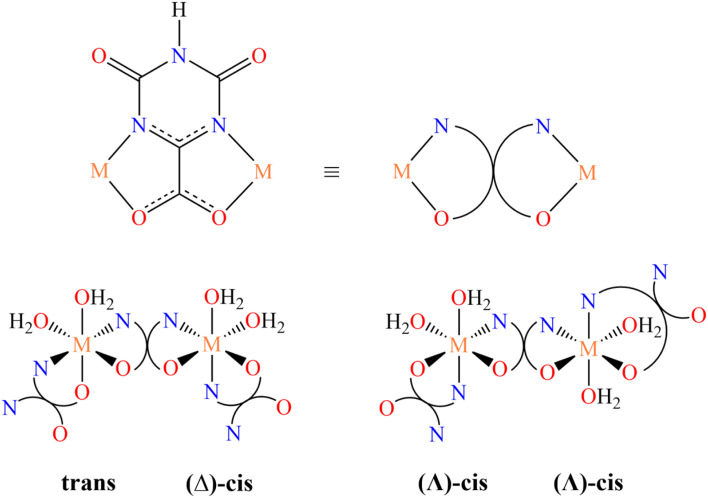
Coordinating and bridging mode of the oxonato ligand in the studied CPs (top). Stereochemistry at the metal center (bottom).

In the present case, we consider a series of one-dimensional CPs containing the versatile oxonato linker (oxo), a bianionic multidentate ligand, exhibiting many coordinating and bridging possibilities, which in the materials synthetized in this study shows only the bidentate chelating and bridging modality illustrated in Scheme 1 (top).

Homometallic CPs based on oxonato linkers have been already reported in the literature for manganese, nickel, cobalt, zinc and iron.^55,56^ Depending on the nature of the metal ion, two different structures can be obtained. For M = Ni,^55^ the asymmetric unit contains two metal ions, one with the nitrogen atoms of oxonato in the *trans* relative position, whereas in the other one they are in the *cis* relative position and the metal center has a *Δ* chirality (Scheme 1, bottom left). For M = Co and Mn,^55^ the asymmetric unit contains one metal ion exhibiting a slightly distorted, *Λ* octahedral stereochemistry with the nitrogen atom in the *cis* relative position (Scheme 1, bottom right).

Heterobimetallic oxonato CPs have never been reported to the best of our knowledge. In addition, oxonato CPs, in general, have never been applied as OER catalysts.

Herein we describe the synthesis and detailed characterization of homometallic and heterobimetallic CPs constituted by the [M(oxo)_2_(H_2_O)_2_] (Mn^2+^, Fe^2+^, Co^2+^, Ni^2+^) coordination unit, and their utilization as electrocatalysts. They were designed to provide insight into cooperative effects between different metal nodes that might lower the overpotential.^48,57^ All systems were synthesized and their electronic and catalytic properties were characterized combining in-depth computational analyses with experimental spectroscopic and cyclic voltammetry measurements. The large number of systems investigated and the integration of theory and experiments allowed us to obtain clear-cut insights into the electronic and geometric effects that influence the oxygen evolution electrocatalytic activity of a material.

## 2. Methods

### 2.1 Mechanistic aspects

The commonly accepted mechanism for the electrocatalytic OER under alkaline conditions involves four electron transfer steps:^26,58^

(i)**M**^**(*n*)**^ + OH^−^ → **M**^**(*n*+1)**^**–OH** + e^−^**M**^**(*n*)**^ + OH^−^ → **M**^**(*n*)**^**˙ ˙OH** + e^−^

(ii)**M**^**(*n*+1)**^**–OH** + OH^−^ → **M**^**(*n*+2)**^**=O** + H_2_O + e^−^**M**^**(*n*+1)**^**–OH** + OH^−^ → **M**^**(*n*+1)**^**˙–˙O** + H_2_O + e^−^**M**^**(*n*)**^**˙ ˙OH** + OH^−^ → **M**^**(*n*+1)**^**˙–˙O** + H_2_O + e^−^

(iii)**M**^**(*n*+2)**^**=O** + OH^−^ → **M**^**(*n*+1)**^**–O–OH** + e^−^**M**^**(*n*+1)**^**˙–˙O** + OH^−^ → **M**^**(*n*)**^**˙ ˙O–OH** + e^−^

(iv)**M**^**(*n*+1)**^**–O–OH** + OH^−^ → **M**^**(*n*)**^ + O_2_ + H_2_O + e^−^**M**^**(*n*)**^**˙ ˙O–OH** + OH^−^ → **M**^**(*n*)**^ + O_2_ + H_2_O + e^−^in which **M** denotes the metal active center of the catalyst and *n* its formal oxidation state. For each oxidative step, an electron can be removed from either the metal or the oxygenated moiety. In the latter case, this process does not lead to a change in the formal oxidation state at the metal center (*vide infra*). In the present example, **M** features a single uncoordinated site that can be occupied by the incoming OH^−^. Computational analysis (*vide infra*) revealed that dissociation of a single water molecule requires a minimum energy penalty (see Tables S14–S24†). Herein, we do not consider the alternative mechanism in which the O–O bond formation involves the coupling of two separate metal-oxo moieties due to the large distance between the metal nodes in the CP studied in the present work. The procedure used to obtain the reaction free energies of these steps from the free energies of the intermediates is described in ref. 26 and 58 (see eqn (S1)–(S17)†).

To elucidate the electronic structure effects on the catalytic activity, it is necessary to compute the energies and properties of CPs for all possible spin states [low-spin (LS), intermediate-spin (IS), and high-spin (HS)] and for all reaction intermediates. Only the spin state with the lowest energy for each intermediate should be considered when drawing reaction free energy profiles. This procedure should be carried out for all the systems investigated in this work in order to draw useful (electronic)structure–activity correlations. Clearly, this requires the accurate calculation of hundreds of relative energies in alkaline solution, which is difficult to achieve with standard computational methodologies.

To address this challenge, we developed a cost-effective yet accurate computational protocol that relies on an “embedded” cluster model approach. A molecular cluster model^59^ was defined with two metal centers fully coordinated by water and oxonato ligands. The environment, *i.e.*, the remaining of the polymer system and (if present) the water solvent, was treated using an implicit solvation approach. The cluster model geometry was obtained from the experimental crystal structure and then optimized at the DFT level. When needed, a series of cluster models were generated for the same polymer to mimic different coordination environment combinations (*vide infra*). For each of these models, the energy and the optical properties were computed for various spin states at the Density Functional Theory (DFT) level using various exchange correlation functionals and computational settings. The resulting absorption spectra were compared with those measured experimentally in the solid state, which served as a calibration point for our computational protocol (see Fig. S12–S14†).

### 2.2 Computational details

All calculations were carried out using the ORCA quantum chemistry program package based on a development version of ORCA 5.0.3.^60^ Due the complex electronic structure of the open shell transition metal systems investigated in this work, the trust-region augmented Hessian approach (TRAH-SCF) was used to facilitate the convergence of the SCF iterations in unrestricted Kohn–Sham calculations. Geometry optimization was carried out using the B3LYP functional together with Grimme's D3 dispersion correction and Becke–Johnson damping.^61,62^ The Ahlrichs def2-TZVP(-f) basis set was used for all the calculations, unless otherwise specified.^63^ The role of the environment was modeled using an implicit solvation scheme (C-PCM).^64^ The dielectric constant of water was used as a model. Thermal and entropy corrections were computed at 298 K. This computational protocol is denoted hereafter as B3LYP-D3+C-PCM(Water)/def2-TZVP(-f). Excited states were calculated at the same level of theory within the TDDFT framework. Unless otherwise specified, the Tamm–Dancoff Approximation (TDA) was used in all TDDFT calculations.

### 2.3 Synthesis, characterization, electrochemical and stability measurements

The synthesis of homometallic [M1M1(oxo)_2_(H_2_O)_2_] CPs was carried out as discussed in ref. 55 and is detailed in Section S3 in the ESI.† Novel heterobimetallic [M1M2(oxo)_2_(H_2_O)_2_] CPs were synthesized analogously, adjusting the relative amounts of the two metal precursors, as described in the ESI.† The crystal structure of representative samples was refined using powder X-ray diffraction (PXRD) data with the Rietveld method, on the basis of literature crystallographic data.^55,56^ UV-Vis spectroscopic measurements of the solid samples were recorded using a Cary4000 (Varian Inc., Palo Alto, California, USA) spectrophotometer, equipped with a 150 mm integration sphere, which uses a barium sulphate tablet as a reference. The spectra are processed with the Kubelka–Munk function.

The electrocatalytic properties of these materials for the OER were investigated in a three-electrode cell containing 1 M KOH solution at room temperature (see Section S4†). A glassy carbon surface was modified with catalysts *via* the drop-casting method and used as the working electrode. Carbon nanotubes (CNTs) were introduced to mix with the electrocatalysts to improve conductivity. Tafel slopes were obtained from linear sweep voltammetry (LSV) curves recorded at a scan rate of 5 mV s^−1^ (Fig. S4†). The stability of CPs under catalytic conditions was tested by continuously cycling the potential from 0.918 to 1.918 V *vs.* RHE at a 100 mV s^−1^ scan rate (Fig. S5†). These findings are corroborated by Raman, SEM, and EDX characterization, as discussed in the following.

## 3. Results and discussion

### 3.1 Geometric and electronic structure of the catalysts

**Fig. 1:**
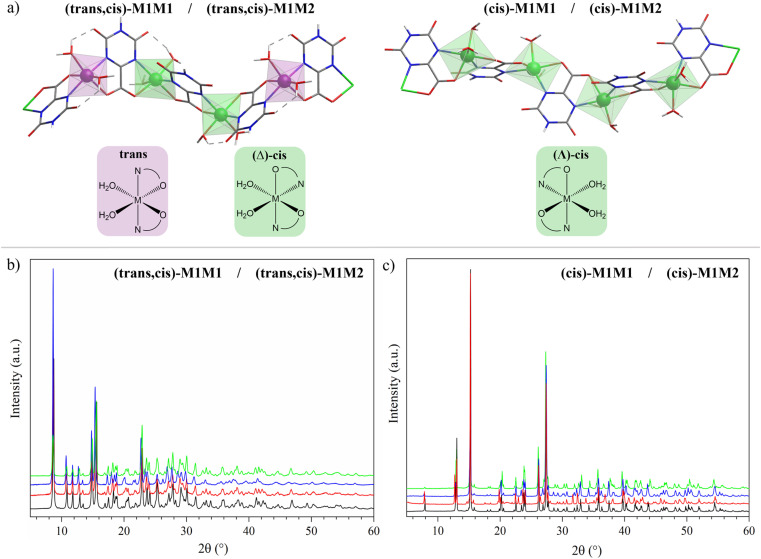
(a) 3D atomic representation of the CP structures for the systems considered in this work. (b) PXRD patterns of (*trans*,*cis*)-NiNi (black), (*trans*,*cis*)-NiCo (red), (*trans*,*cis*)-NiMn (blue), and (*trans*,*cis*)-NiFe (green). (c) PXRD patterns of (*cis*)-CoCo (black), (*cis*)-FeMn (red), (*cis*)-CoFe (blue), and (*cis*)-CoNi (green) samples.

The structures and labels of the synthesized CPs are shown in Fig. 1a. Depending on the metals used in the synthesis, two different CP families were identified through X-ray characterization featuring slightly different coordination environments for the metal ions, in which the nitrogen atoms of the oxonato ligands are placed in the relative *trans* or *cis* position.

In the *trans* environment, the two nitrogen atoms (blue) are on opposite faces of the octahedron, while in the *cis* environment the two nitrogen atoms are coordinated 90° apart from one another with respect to the metal center. Henceforth, we will denote as (*trans*,*cis*)-M1M1 the homometallic polymers where the *trans* and the (*Λ*)-*cis* coordination environments alternate along the structure, and as (*cis*)-M1M1 the ones where the nodes show only the (*Δ*)-*cis* coordination environment (M = Mn, Fe, Co, Ni). For the heterobimetallic polymers, we will use the notation (*trans*,*cis*)-M1M2 and (*cis*)-M1M2. These two families were easily identified by powder X-ray diffraction (PXRD) characterization, because they showed different patterns (Fig. 1b and c). Within each family, the powder patterns are only slightly affected by the nature of metal centers, thus indicating that the introduction of a second metal ion does not lead to any significant structural variation with respect to the homometallic counterparts. Rietveld refinements of the structure of (*cis*)-CoCo, (*trans*,*cis*)-NiNi, and (*trans*,*cis*)-NiMn using PXRD data from a conventional diffractometer provided results in close agreement with the reference structural data.^55,56^

**Fig. 2:**
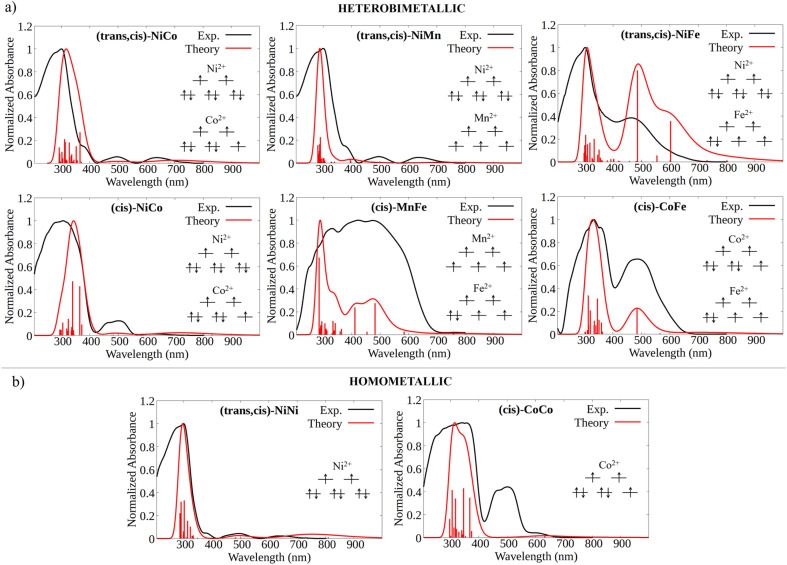
(a) Computed (red) *vs.* experimental (black) UV/Vis spectra for the heterobimetallic CPs, together with an illustrative scheme of the electronic configuration of the d orbitals at the metal centers. For the (*trans*,*cis*)-M1M2 case, the computed spectrum is the convolution of the spectra obtained considering both possible cluster environment configurations, *i.e.*, M1(*trans*)M2(*cis*) and M1(*cis*)M2(*trans*). (b) Computed (red) *vs.* experimental (black) UV/Vis spectra for the homometallic CPs, together with an illustrative scheme of the electronic configuration of the d orbitals in their pseudo-octahedral ligand field.

The experimental absorption spectra of the solid samples were compared with those obtained at the DFT level (Fig. 2a and b). For all systems, computational analysis (see Fig. S25–S30†) revealed that the band at the highest energy (below 400 nm) is characterized by electronic transitions from delocalized metal–ligand orbitals to the π* orbital of the oxonato ligand 
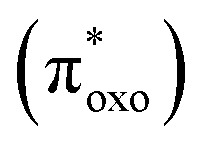
. In all Fe-containing systems, *i.e.*, (*trans*,*cis*)-NiFe, (*cis*)-CoFe, and (*cis*)-MnFe, additional intense absorption features are observed in the 400–600 nm range and they all correspond to Fe-ligand → 
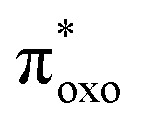
 excitations. For the other samples ((*trans*,*cis*)-NiCo, (*trans*,*cis*)-NiMn, (*cis*)-NiCo, (*trans*,*cis*)-NiNi and (*cis*)-CoCo), visible features with lower intensity are observed which can all be attributed to localized d–d transitions. For the Co-containing CPs, such features appear with higher intensity in the experimental spectra compared to the computed spectra.

It is important to emphasize here that the metal center spin state has a huge influence on the CP photophysical properties, influencing both the intensity and the energy of the key electronic transitions (see Fig. S15–S18†). However, the absorption spectra computed for the DFT ground state match well with those experimentally measured. This remarkable agreement between theory and experiment served as a first validation of our computational protocol and allowed us to establish the most likely spin state at the metal centers for all the CPs considered in this work.

### 3.2 Electrochemical measurements

**Fig. 3:**
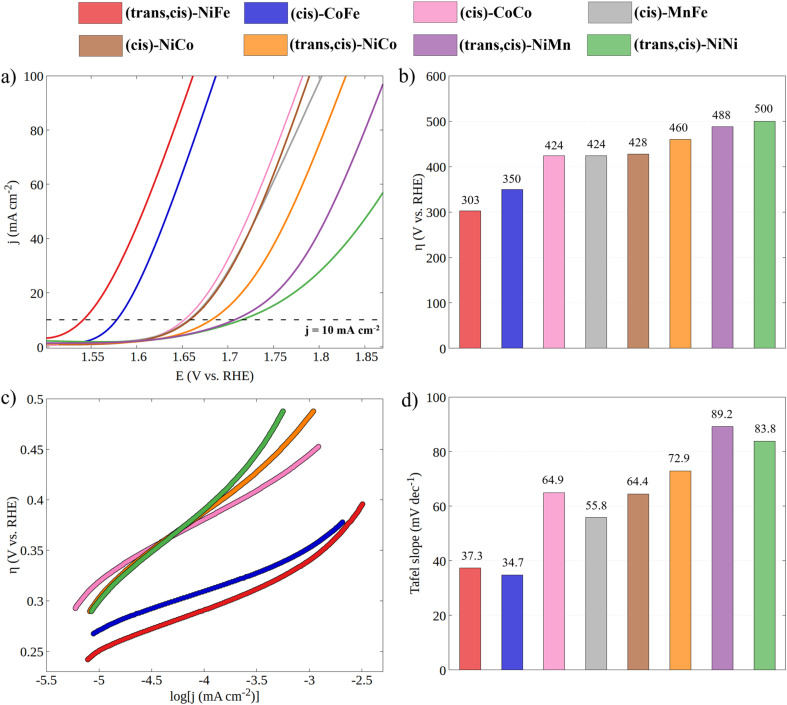
(a) Cyclic voltammetry curves for all synthesized CPs in 1 M KOH with a scan rate of 100 mV s^−1^ (the reverse scan was omitted for clarity). (b) Experimental overpotentials of CPs obtained at *j* = 10 mA cm^−2^. (c) Tafel plots obtained from LSV curves recorded at a 5 mV s^−1^ scan rate (see Fig. S3†) and (d) histogram of Tafel slopes.

Having established the geometric and electronic structures of the catalysts in their ground state, we moved to the characterization of their electrocatalytic properties through cyclic voltammetry experiments under alkaline conditions (Fig. 3). It is important to emphasize that the incorporation of a second metal in the CP-backbone did not result in any change of its structure, both in terms of the coordination surrounding of the metal center and in the topological structure of the CP, as confirmed by UV-Vis and PXRD data. Thus, the electrocatalytic performances of CPs can be attributed, to a large extent, to the intrinsic activity of the active site, which depends on the specific electronic properties of the metal ion (see the note for Fig. S6 and S7†).

The electrocatalytic performance of CPs was compared by evaluating their overpotential at a current density of 10 mA cm^−2^ (*η*_10_). The *η*_10_ for each catalytic system was obtained by averaging the values recorded from the first CV of four independently prepared working electrodes (WEs). As shown in Fig. S3,† the excellent overlapping of the CVs of different working electrodes demonstrates the good reproducibility of the catalytic system and the accuracy of the results. The stability of CPs under catalytic conditions was tested by continuously cycling the potential from 0.918 to 1.918 V *vs.* RHE at a 100 mV s^−1^ scan rate (Fig. S5†). The gradual decrease in catalytic current indicates the limited stability of CPs under OER electrocatalytic conditions. However, since the *η*_10_ is taken from the first cycle, where no degradation has occurred, the electrocatalytic performances can be directly related to the electronic structure of the as-synthesized CPs.

Both the measured overpotentials and the Tafel slopes show a clear catalytic trend, highlighting the direct dependence of the CP activity on the metal nature. Remarkably enough, the most efficient CPs (*i.e.*, those with a lower overpotential) contain Fe ions. In particular, the catalyst with the lowest overpotential is (*trans*,*cis*)-NiFe (303 mV), which is closely followed by (*cis*)-CoFe (350 mV). These figures well compare with that of the benchmark IrO_2_ (289 mV) electrocatalyst. The excellent OER kinetics of Fe-containing CPs, approaching that of the best performing materials,^65^ was further highlighted by their smaller Tafel slope. For instance, extremely low Tafel slopes of 37.3 and 34.7 mV dec^−1^ were obtained for (*trans*,*cis*)-NiFe and (*cis*)-CoFe, respectively. These results suggest that the Fe centers, which are present in both systems, feature an intrinsic catalytic activity that is only slightly modulated by ion mixing with different metals. The fact that homometallic (*cis*,*trans*)-NiNi (*η*_10_ = 500 mV and Tafel slope = 83.8 mV dec^−1^) and (*cis*)-CoCo (*η* = 424 mV and Tafel slope = 64.9 mV dec^−1^) feature widely different catalytic activity provides further evidence of the special role of the Fe centers in this context. This finding is especially interesting considering that, in most cases of the electrocatalytic OER on CPs, it is generally assumed that the reaction occurs at the Ni sites whenever Ni ions are present.^54^ Interestingly, recent computational studies^50,51,66^ suggested a significant activity for the Fe centers in 1D coordination polymers, MOFs and metal oxides, which would be consistent with the experimental findings presented in this work. These intriguing results deserve a more detailed discussion and computational analysis.

### 3.3 Stability analysis

In this section, our aim is to assess the stability of the CPs under catalytic conditions in order to establish a solid foundation upon which the exploration of their structure–activity relationship is initiated. Hence, Raman spectra, SEM images and EDX analysis were carried out in order to characterize the (*trans*,*cis*)-NiFe sample structurally and morphologically.

**Fig. 4:**
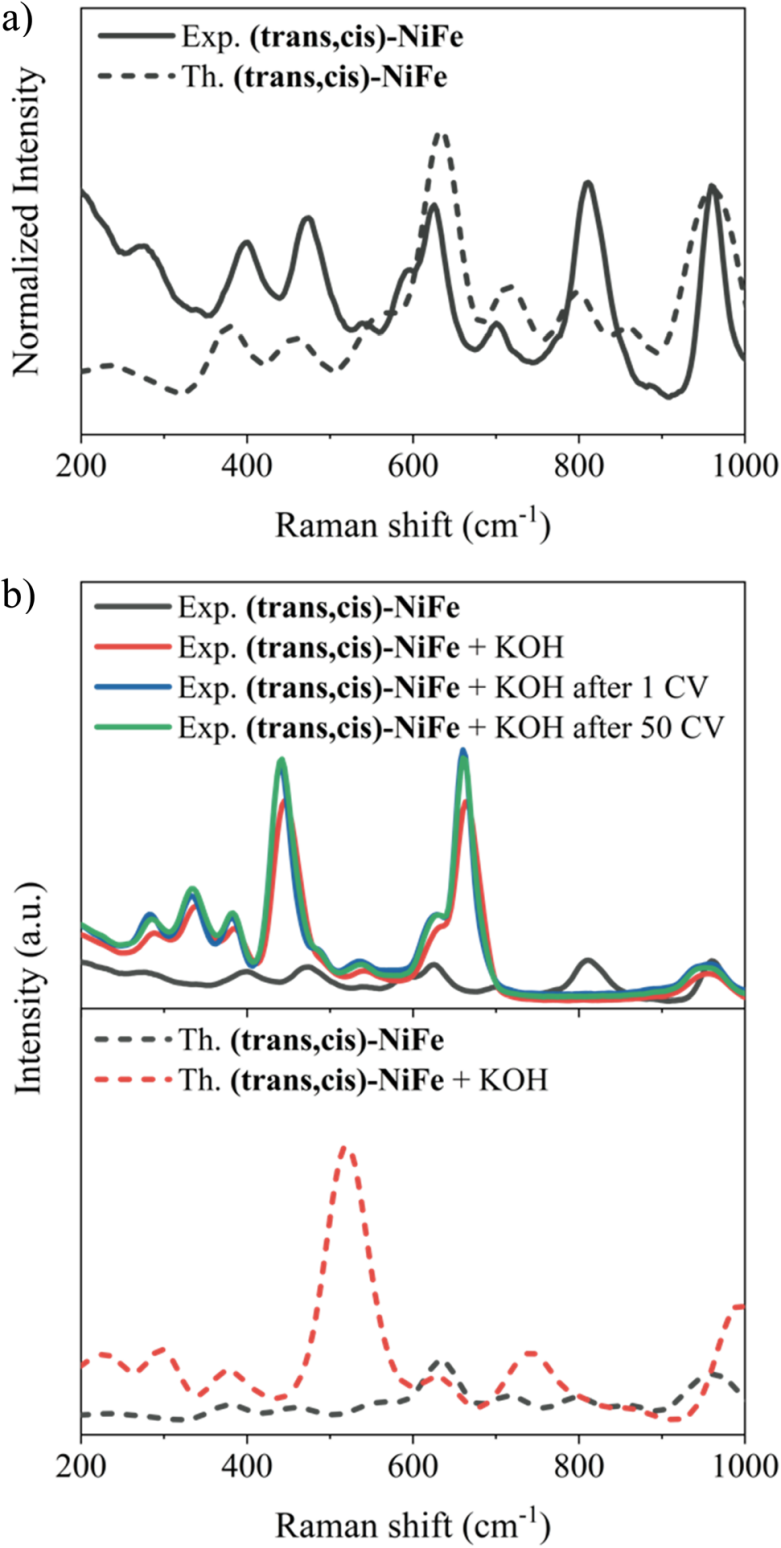
(a) Experimental Raman spectrum of embedded (*trans*,*cis*)-NiFe (solid black) and the computed one. (b) Top: experimental Raman spectra of embedded (*trans*,*cis*)-NiFe (solid grey), after KOH treatment (solid red), after 1 CV (solid blue) and after 50 CV (solid green). Bottom: computed Raman spectra for (*trans*,*cis*)-NiFe (dashed grey) and (*trans*,*cis*)-NiFe with two OH^−^ coordinated to Fe (dashed red).

Initially, matrix effects on the Raman signals were evaluated, and no changes in the band structure were identified once the polymer is embedded in the carbon nanotube, as shown in Fig. S8.† The comparison between the computed Raman spectrum for our cluster model and the experimental spectrum associated with the embedded polymer is reported in Fig. 4a. Strikingly, the computed and experimental spectra exhibit remarkable similarity, with all experimental features accurately replicated. The characteristic signals of Ni–O bonding^67,68^ (474.5 and 544.6 cm^−1^) and Fe–O/N vibrations^69,70^ (599.8 cm^−1^) are observed. The band centred at 628.7 and 700.5 cm^−1^ could be assigned to the displacement of the C–N bond connected to the Fe atom.^71^

To assess the stability of the catalyst under operando conditions, these analyses were repeated on the material under three different experimental conditions: (i) after treatment with a 1 M KOH solution; (ii) after 1 CV; (iii) after 50 CVs (Fig. 4b, top panel).

An obvious change in the structure of the Raman signals was observed upon treatment with 1 M KOH solution at zero potential, with several signals showing a remarkable increase in intensity. This effect could be attributed to the change in the polarization of the bonds following treatment with KOH, *e.g.*, due to partial deprotonation of the H_2_O molecules coordinated to the metal centres. To verify this hypothesis, calculations were run to assess the origin of the change in the shape and intensity of the Raman peaks after treatment with KOH. Specifically, Raman spectra were computed by gradually deprotonating the water molecules coordinated to the two metals. Fig. 4b (lower panel) shows the results obtained for the cluster model where Fe is coordinated by two OH^−^. The remarkable agreement between theory and experiment in this case provides strong evidence that the changes observed in the Raman features upon treatment with KOH are associated with water deprotonation. This has a strong influence on the Fe–O_water_ vibrations, which are drastically affected in both energy and intensity.

**Fig. 5:**
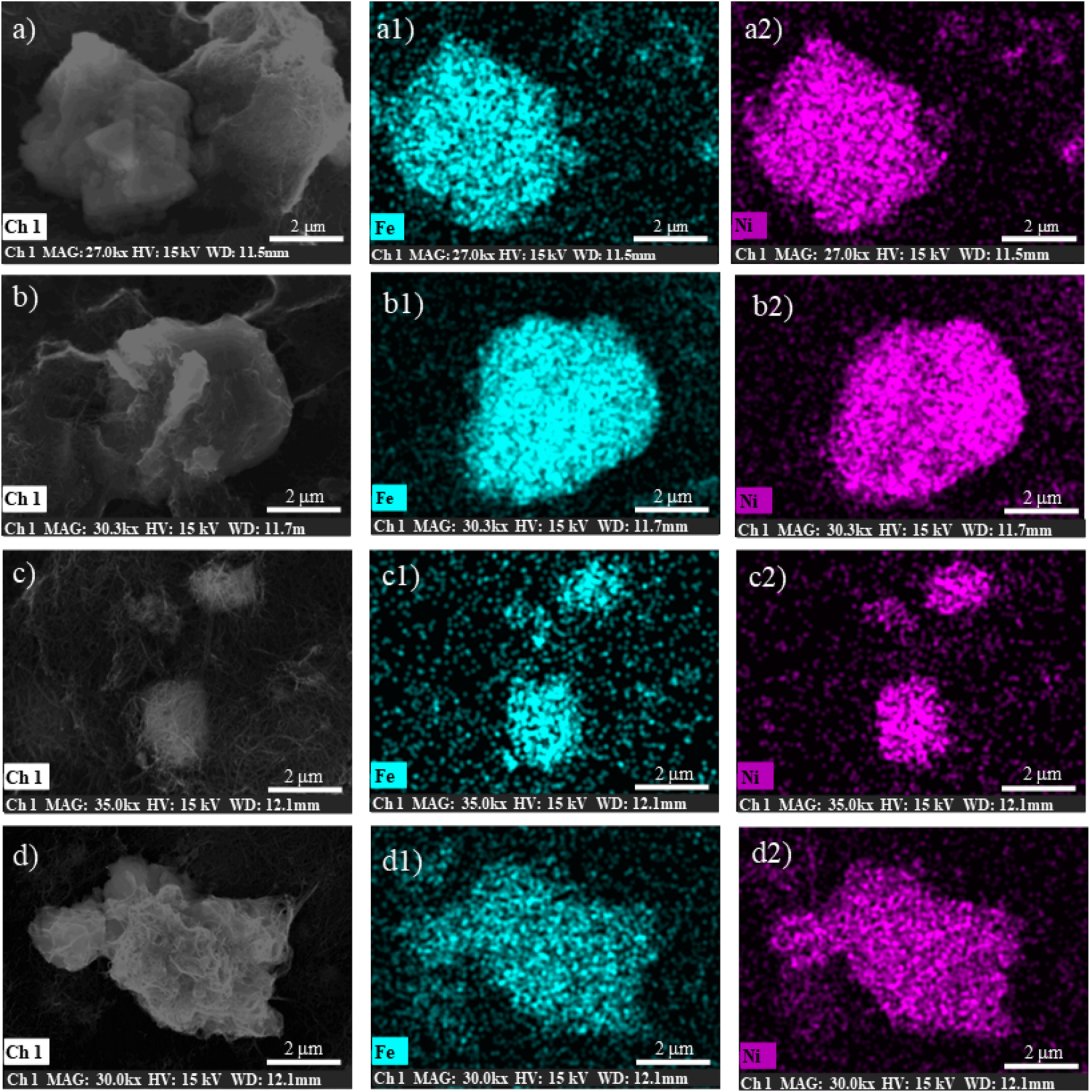
EDX elemental analysis of Fe (a1)–(d1) and Ni (a2)–(d2). (a) NTC matrix + (*trans*,*cis*)-NiFe untreated; (b) NTC matrix + (*trans*,*cis*)-NiFe treated with KOH; (c) NTC matrix + (*trans*,*cis*)-NiFe treated with KOH after 1CV; (d) NTC matrix + (*trans*,*cis*)-NiFe treated with KOH after 50 CVs.

It is important to emphasize here that no further changes in the band shape of the signals are noticeable after the material has been subjected to 50 CVs, indicating that the catalyst remains stable under catalytic potentials. Indeed, elemental EDX analysis (Fig. 5 and Table S3†) and SEM images (Fig. S9 and S10†) provide further evidence for the stability of the catalysts, demonstrating that there are no particular morphological or compositional changes in the analysed samples under operando conditions.

Overall, the combination of experimental and computational results provides compelling evidence for catalyst stability under the experimental conditions.

### 3.4 Identification of the active metal centers

For CPs featuring different metals (heterobimetallic) and/or coordination environments (*cis* or *trans*), the OER mechanism was investigated computationally by considering all possible metal/coordination environment combinations. The combination leading to the lower computed overpotential was identified as the active site of the reaction. The computed and experimental overpotentials for all the systems investigated in this work are compared in Table 1.

**Table 1:** Experimental (*η*_exp_) and computed (*η*_theory_) overpotentials and rate determining step (RDS) for the OER on CPs. The active metal is emphasized in red. Unless otherwise specified, the active metal was placed in the *cis* coordination environment

CP	*η*_exp_ (eV)	*η*_theory_ (eV)	RDS
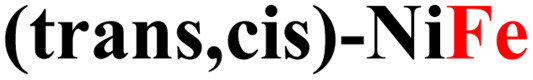	0.30	0.56^a^	**MOH** → **MO**
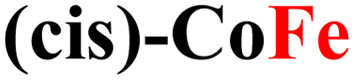	0.35	0.52	**MO** → **MOOH**
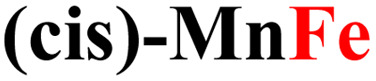	0.42	0.59	**MO** → **MOOH**
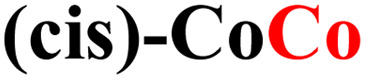	0.42	0.60^b^	**MOH** → **MO**
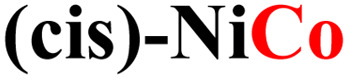	0.43	0.69	**MOH** → **MO**
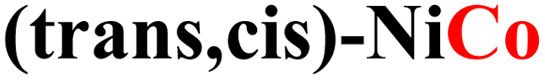	0.46	0.68	**MOH** → **MO**
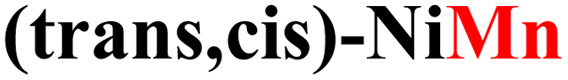	0.49	0.86	**MOH** → **MO**
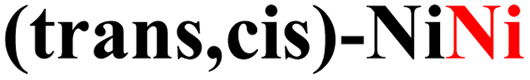	0.50	0.94^c^	**MOH** → **MO**

a For the OER on the Fe site *η*_theory_ = 0.56 eV for both *cis* and *trans* coordination environments. For the OER on the Ni site, *η*_theory_ = 0.95 eV.

b If the electronic structure of the active site is constrained to ^4^Co(ii), *η*_theory_ = 0.92 eV.

c If the electronic structure of the active site is constrained to ^3^Ni(ii), *η*_theory_ = 1.27 eV.

While the computed overpotentials are shifted to higher values than the experimental ones, the overall catalytic trend is retained, which provides another validation of our computational protocol as well as further evidence for the structural stability of CPs. This is especially remarkable considering the drastically different electronic properties of the metal centers (*vide infra*) and the complex nature of the systems investigated in this work. Again, the most active catalysts contain Fe^2+^ ions, followed by those incorporating Co^2+^ and finally by Ni^2+^.

In all cases, the rate determining step of the reaction is **MOH** → **MO**. The only exceptions were found for (*cis*)-CoFe and (*cis*)-MnFe, for which the **MOH** → **MO** and **MO** → **MOOH** steps feature essentially the same energy. As it will be discussed in the following sections in greater detail, this effect originates from the peculiar electronic properties of the Fe centers.

Remarkably enough, spin effects play a fundamental role in these systems. Enforcing the same spin configuration at the metal center along the reaction profile introduces a huge error in the overpotential estimate, which amounts to *ca.* 0.3 eV for both (*trans*,*cis*)-NiNi and (*cis*)-CoCo. In the former, neglecting spin transitions even leads to a change in the estimated rate determining step of the reaction from **MOH** → **MO** to **M** → **MOH**. Importantly, (*trans*,*cis*)-NiFe and (*trans*,*cis*)-NiNi feature almost identical overpotentials when the reaction takes place at the Ni(*cis*) site. This provides further evidence that cooperativity effects between different metal centers are, to a large extent, either independent from the metal nature or negligible for the CPs considered in this work, consistent with the experimental findings discussed above. In addition, the results obtained for (*trans*,*cis*)-NiFe for different metal sites suggest that the Fe nodes (in either *cis* or *trans* configurations) are indeed the active sites of the reaction, which is again consistent with experimental observations. Interestingly, the *cis*/*trans* nature of the coordination environment has a negligible effect on the overpotential (therefore, unless otherwise specified, only the *cis* coordination environment is considered in the subsequent calculations). It can thus be concluded that in heterobimetallic CPs one transition metal acts as the main active site while the other has a structural role.

### 3.5 Metal electronic structure evolution along the reaction profile

**Fig. 6:**
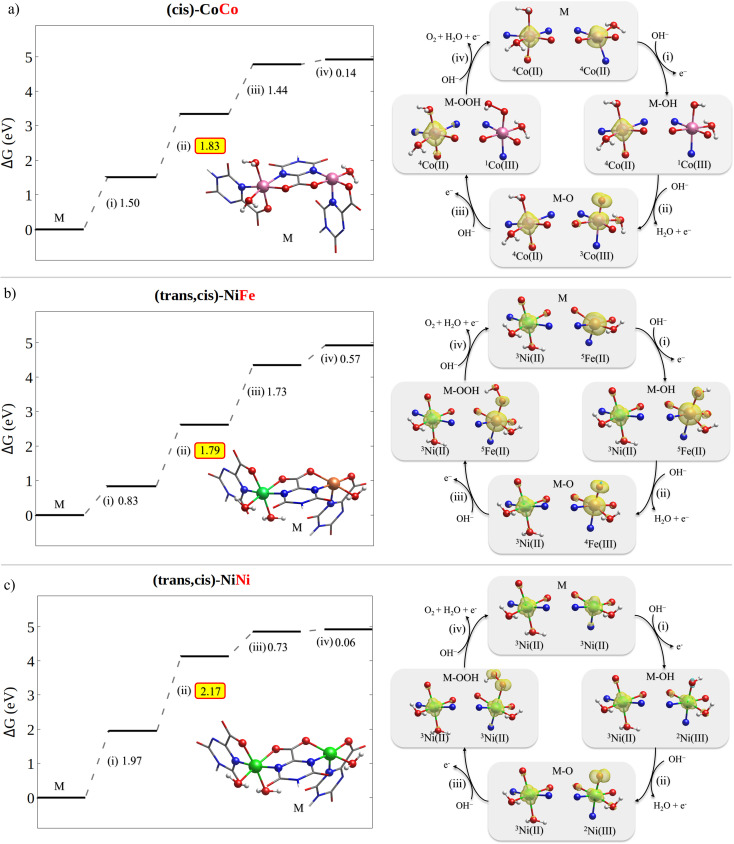
OER free energy profile (left) and spin density evolution along the reaction path (right) for (a) (*cis*)-CoCo, (b) (*trans*,*cis*)-NiFe and (c) (*trans*,*cis*)-NiNi. To facilitate the chemical interpretation of the results, the metal oxidation state and local spin configuration are also reported. They were determined using Mulliken spin populations (they were rounded up to the nearest integer). The free energies for the other catalysts considered in this work can be found in Tables S14–S24.†

The free energy diagrams for the OER on (*cis*)-CoCo, (*trans*,*cis*)-NiFe and (*trans*,*cis*)-NiNi are shown in Fig. 6, together with a graphical representation of the corresponding variations in the metal electronic structure along the reaction profile. For the sake of clarity, the formal oxidation state and the estimated spin multiplicity at the metal centers are shown in Fig. 6. However, since electrons are typically removed from orbitals that are delocalized between the metal and the ligand, such assignments should be taken with caution. During the OER, the actual oxidation state of the metal centers is best described in a continuum that ranges from +2 (the electron is removed from the ligand) to +3 (the electron is removed from the metal), as discussed in Section 2.1.

Importantly, our computational results indicate that, once the active metal node is oxidized, the oxidation of the neighboring metal nodes is disfavored. For example, the potential required to oxidize nickel(ii) to nickel(iii) in (*trans*,*cis*)-NiFe increases by *ca.* 0.2 V after step (i) on Fe. These results are consistent with prior experimental studies from the group of Prof. Peter Strasser,^72^ which demonstrated the presence of a large fraction of reduced Ni(ii) in Ni–Fe catalysts at catalytic potential. For this reason, in the OER profiles shown in Fig. 6, the oxidation state of the neighboring metal nodes was kept constant thorough the reaction at the value it has in the catalyst ground state (+2).

For these systems, the rate determining step is the PCET leading to the formation of the **MO** intermediate. This is the most common situation among the catalysts studied in this work. Importantly, different metals show different evolution of the electronic configuration along the reaction path.

The OER on (*cis*)-CoCo is characterized by multiple spin transitions along the reaction path (see Fig. 6a). Step (i) leads to a change in both the formal oxidation state and the spin configuration of the active metal, which goes from Co(d^7^)/HS in **Co(****iii****)** to Co(d^6^)/LS in **Co(****iii****)–OH**. After the subsequent rate determining step (ii), the metal assumes a Co(d^6^)/IS configuration with two unpaired electrons, while one additional unpaired electron is located at the oxygen [**Co(****iii****)˙–˙O**]. Importantly, this step is associated with a significant shortening of the key Co–O bond distance from 1.86 Å [**Co(****iii****)–OH**] to 1.63 Å [**Co(****iii****)˙–˙O**], which is accompanied by a corresponding increase in the Co–O Mayer bond order from 0.91 to 1.71. These results suggest that for (*cis*)-CoCo the adsorption energy of the oxygen containing ligand increases from **MOH** to **MO**. To a large extent, this effect is responsible for the low overpotential computed for this system, consistent with the experimental findings. The subsequent step (iii) leads to a Co(d^6^)/LS metal with no unpaired electrons [**Co(****iii****)–OOH**]. Both the Co–O distance and the Mayer bond order in **Co(****iii****)–OOH** are similar to those obtained for **Co(****iii****)–OH**, being 1.88 Å and 0.89, respectively. Finally, step (iv) regenerates the catalyst and releases O_2_.

The OER on (*trans*,*cis*)-NiFe shows a different evolution of the electronic structure of the metal center along the reaction path. As discussed above, the active site in this case is the Fe center, which invariably assumes the highest possible spin configuration (Fig. 6b). Step (i) in this case does not change significantly either the oxidation state or the electronic structure of the metal, which can be described as Fe(d^6^)/HS for both **M** [**Fe(****ii****)**] and **MOH** [**Fe(****ii****)˙ ˙OH**]. In contrast, the rate determining step (ii) oxidizes the metal to Fe(d^5^)/HS [**Fe(****iii****)˙–˙O**]. Consistent with the (*cis*)-CoCo case, this step is associated with a significant shortening of the Fe–O bond (1.86 Å → 1.63 Å) and with an increase in the Fe–O Mayer bond order (1.0 → 1.8). Again, these results are consistent with an increase in the ligand adsorption energy in the rate determining step of the reaction, and hence with the low overpotential experimentally measured. Step (iii) gives back Fe(d^6^)/HS [**Fe(****ii****)–OOH**], a M–O bond distance of 1.90 Å and a Mayer bond order of 0.93. The last step regenerates the catalysts and releases O_2_.

The variations in the electronic structure of Ni during the OER on (*trans*,*cis*)-NiNi are especially interesting (Fig. 6c). Step (i) in this case removes one electron from the metal center, leading to a Ni(d^7^) metal with a single unpaired electron [**Ni(****iii****)–OH**]. The free reaction energy for this step amounts to 1.97 V, indicating that ligand absorption in this system is comparatively less favorable than in (*cis*)-Co and (*trans*,*cis*)-NiFe. The subsequent rate determining step (ii) oxidizes the axial ligand, leaving the metal center largely unaltered in its Ni(d^7^)/LS state [**Ni(****iii****)˙–˙O**]. These results differ from those just discussed for different active metals, in which the metal is either oxidized or promoted to a different spin state in the rate determining step. Remarkably enough, only slight variations in the Ni–O distances and bond orders were observed in this case; the Ni–O bond decreases from 1.84 Å in **MOH** to 1.78 Å in **MO**, which is accompanied by a minor increase in the Ni–O Mayer bond order (0.89 → 0.92). These results suggest that for (*trans*,*cis*)-NiNi the ligand adsorption energy does not increase significantly on going from **MOH** to **MO**, in striking contrast to the other cases discussed above. This observation is consistent with the larger computed and experimental overpotentials for (*trans*,*cis*)-NiNi. Finally, the Ni center in **MOOH** can be described as Ni(d^8^) with two unpaired electrons [**Ni(****ii****)˙ ˙O–OH**]. Interestingly, the Ni–O distance in **MOOH** amounts to 2.22 Å, while the corresponding Mayer bond order is just 0.26. These results suggest an extremely weak ligand adsorption energy in **MOOH** for this system, which is consistent with the computed free energy variation for step (iv).

### 3.6 Breaking linear scaling relationships

The results just described emphasize that variations in the electronic structure of the metal center have a huge impact on the intermediate adsorption energies and hence on the catalyst efficiency. We believe that this effect can be exploited in practical applications.

For example, it has been shown^26,27^ that the energies of the **MOH** and **MOOH** intermediates are strongly related, displaying a linear correlation over a large number of systems with different nature and properties. The chemical origin of this effect has been attributed to the bond order conservation principle.^73^ The linear correlation implies that the free energy difference between those intermediates is roughly constant, and in fact it typically amounts to *ca.* 3.2 eV (the exact value might change depending on the specific exchange correlation functional employed for the calculations).^26,27^ As each step in the OER mechanism has a thermodynamic limit of 1.23 eV, it poses a theoretical limit of 0.3–0.4 V (3.2–1.23 × 2 eV) to the minimal overpotential that can be obtained for the OER. Our hypothesis is that inducing a change in the electronic structure of the metal along the energy profile, *e.g.*, through appropriate ligand design strategies, could in principle influence the relative adsorption energies of the oxygen-containing ligands, thus breaking the relationship between the free energies of the intermediates along the reaction profile.

For (*cis*)-CoCo and (*trans*,*cis*)-NiFe, this is not the case. The metal centers in these systems feature the same electronic structure in both **MOH** and **MOOH**, *i.e*., Co(d^6^)/LS and Fe(d^6^)/HS for (*cis*)-CoCo and (*trans*,*cis*)-NiFe, respectively. In addition, no significant variations were observed in the metal–ligand distance and bond order between these two intermediates. Consistent with the discussion above, the free energy difference between these intermediates is indeed fairly close to 3.2 eV for both catalysts, being 3.27 eV and 3.52 eV for (*cis*)-CoCo and (*trans*,*cis*)-NiFe, respectively. The excellent performance of (*trans*,*cis*)-NiFe originates from the fact that the free energy of the **MO** intermediate lies exactly halfway between those of **MOH** and **MOOH**, and hence the two **MOH** → **MO** and **MO** → **MOOH** steps require exactly the same energy.

In contrast, (*trans*,*cis*)-NiNi shows a different metal electronic structure for **MOH** [Ni(d^7^) with one unpaired electron] and **MOOH** [Ni(d^8^) with two unpaired electrons]. Remarkably enough, the energy difference between the **MOH** and **MOOH** intermediates in this case is just 2.90 eV, which is lower than that computed for all the other systems investigated in this work. In this case, this partial breaking in the linear scaling relationships does not lead to a lower overpotential due to the large reaction free energies associated with steps (i) and (ii). For step (ii), this effect originates from the peculiar electronic structure of the **MO** intermediate in this system (see the discussion above), which is closer in energy to **MOOH** than to **MOH**. However, these results confirm our assumption that variations in the electronic structure of the metal center can in principle be exploited to break the linear scaling relationships between the OER intermediates.

**Fig. 7:**
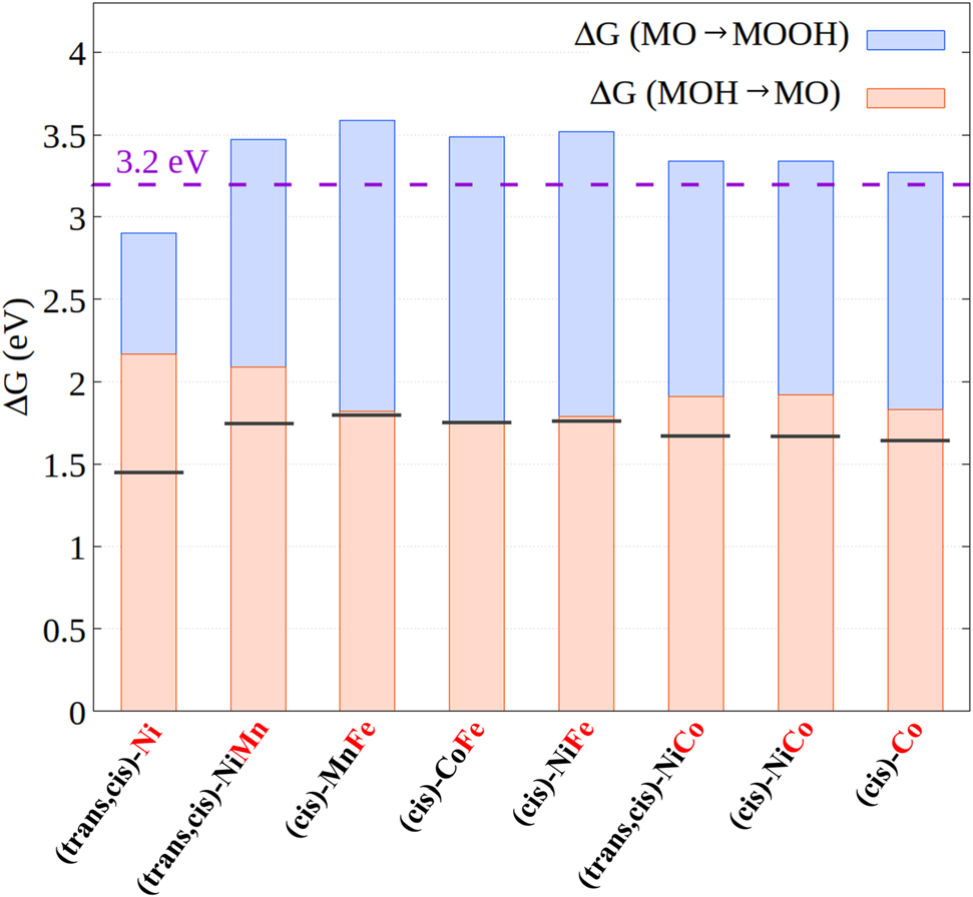
Free energy difference between the **MOOH** and **MOH** intermediates as well as its decomposition into the **MOH** → **MO** (orange bar) and **MO** → **MOOH** (blue bar) reaction free energies. The horizontal black lines represent the ideal case in which both steps have the same energy. The dashed violet horizontal line represents the theoretical value for the energy difference between **MOOH** and **MOH** obtained from linear scaling relationships. The active metal is emphasized in red.

To explore the validity of these findings over a broader set of systems, we report in Fig. 7 the relative energy between the **MOOH** and **MOH** intermediates, as well as its decomposition into the free energy associated with the **MOH** → **MO** and **MO** → **MOOH** steps for all the CPs investigated in this work.

Based on these data, three main considerations can be drawn concerning the relationship between the metal electronic structure in coordination polymers and their catalytic activity:

(i) The electronic interaction between different metal centers has a weak influence on the free energy profiles and hence on the catalytic activity, consistent with the experimental results discussed above. Thus, ion mixing has mainly a structural effect in the systems considered here. However, the use of ligands with different electronic and geometric properties could have a deep impact on metal–metal cooperativity, and hence different ligand/metal combinations should be explored in future studies.

(ii) When the active site is Fe, the **MOH** → **MO** and **MO** → **MOOH** steps always feature essentially the same energy, as in the ideal catalyst case. This effect originates from electronic structure effects modulating the ligand adsorption energies in these systems. This seems to be the main effect responsible for the greater efficiency for the electrocatalytic OER on Fe-containing CPs, consistent with the experimental observation.

(iii) The only system featuring a free energy difference between the **MOOH** and **MOH** intermediates that is lower than the 3.2 eV limit is (*trans*,*cis*)-NiNi, which is also the only system for which the electronic structure of the metal center changes on going from **MOH** to **MOOH**. Hopefully, the electronic structure effects of this kind could be exploited in practical applications to circumvent the theoretical limit for the electrocatalytic OER. Novel design strategies should be explored to selectively stabilize specific reaction intermediates along the reaction profile, *e.g.*, by modulating the electron-donating ability of the ligand. Efforts to exploit such electronic structure effects are currently underway in our laboratory.

## 4. Conclusions

This work provides evidence for the notion that electronic structure effects, such as spin transitions or variations in the transition metal oxidation state during the electrocatalytic OER, play a fundamental role in determining the reaction efficiency; manipulating the electronic structure of the reaction intermediates with appropriate design strategies could pave the way for the development of new catalysts with lower overpotentials. A systematic exploitation of (electronic) structure–property relationships in hydrogen production technologies is hampered by the complexity of the catalytic system, which limits the ability of current experimental and computational methodologies to provide detailed information into the evolution of the metal electronic structure during the reaction.

In the case study presented here, we discussed one-dimensional coordination polymers of earth-abundant metals, which are characterized by active sites with well-defined geometric and electronic properties that can be readily correlated with their oxygen evolution electrocatalytic activity. In particular, we focused on one-dimensional coordination polymers in which metal ions (Ni^2+^, Co^2+^, and Fe^2+^), in a pseudo-octahedral environment, bearing two oxonato bridging ligands and two water molecules in the first coordination sphere. Both homometallic and novel heterobimetallic CPs were synthesized and their oxygen evolution electrocatalytic activity under alkaline conditions was determined experimentally. The electronic structure of catalysts and reaction intermediates was characterized combining spectroscopic measurements with an experimentally validated computational protocol.

The acquired mechanistic information suggests that in this case the OER involves a single metal center. The most active centers are Fe^2+^ ions, followed by Co^2+^ and finally by Ni^2+^. Ion mixing has a secondary effect, indicating that the cooperativity effects of different metals play a secondary role in these systems. The greater efficiency of the Fe centers was attributed to their ability to provide almost identical free energies for the **MOH** → **MO** and **MO** → **MOOH** steps, consistent with the ideal catalyst case. In all cases, the variations of the metal electronic structure during the OER were correlated with the catalyst activity. It was found that spin transitions lower the overpotential by as much as 0.3 eV and that electronic structure effects modulating the ligand adsorption energy are crucial to increase catalyst efficiency. Finally, we demonstrated that a change in the electronic structure of the metal along the energy profile can be used to break the linear scaling relationship between the energies of the **MOH** and **MOOH** intermediates, which currently poses a theoretical lower bound to the experimental overpotential. We believe that our study provides the basis to exploit such electronic structure effects in water splitting technologies through appropriate ligands.

## Data availability

The computational and experimental procedures as well as all the relevant data generated and analysed in this study are available in the manuscript and its ESI.† Additional raw and unprocessed data are available from the corresponding author on reasonable request.

## Author contributions

L. B. performed all the calculations. L. B. prepared most of the figures in the Article. G. M. R. conceptualized and synthesized all catalysts. I. D. A. and G. M. R. tested their catalytic performances. A. M. G. and L. L. performed UV-vis, Raman, SEM and EDX measurements and analyzed the data preparing the graphs and Figures. R. V. performed PXRD characterization. L. B. and G. B. wrote the original draft of the manuscript with input from all authors. All authors discussed the results and contributed to the final version of the manuscript. G. M. R. and G. B. directed the study.

## Conflicts of interest

There are no conflicts to declare.

## Supplementary Material

SC-015-D3SC05891C-s001

## References

[cit1] Ursua A., Gandia L. M., Sanchis P. (2012). Hydrogen Production from Water Electrolysis: Current Status and Future Trends. Proc. IEEE.

[cit2] Abdin Z., Zafaranloo A., Rafiee A., Mérida W., Lipiński W., Khalilpour K. R. (2020). Hydrogen as an Energy Vector. Renewable Sustainable Energy Rev..

[cit3] Chatenet M., Pollet B. G., Dekel D. R., Dionigi F., Deseure J., Millet P., Braatz R. D., Bazant M. Z., Eikerling M., Staffell I., Balcombe P., Shao-Horn Y., Schäfer H. (2022). Water Electrolysis: From Textbook Knowledge to the Latest Scientific Strategies and Industrial Developments. Chem. Soc. Rev..

[cit4] Song J., Wei C., Huang Z.-F., Liu C., Zeng L., Wang X., Xu Z. J. (2020). A Review on Fundamentals for Designing Oxygen Evolution Electrocatalysts. Chem. Soc. Rev..

[cit5] Huynh M., Ozel T., Liu C., Lau E. C., Nocera D. G. (2017). Design of Template-Stabilized Active and Earth-Abundant Oxygen Evolution Catalysts in Acid. Chem. Sci..

[cit6] Hu C., Zhang L., Gong J. (2019). Recent Progress Made in the Mechanism Comprehension and Design of Electrocatalysts for Alkaline Water Splitting. Energy Environ. Sci..

[cit7] Wang S., Lu A., Zhong C.-J. (2021). Hydrogen Production from Water Electrolysis: Role of Catalysts. Nano Convergence.

[cit8] Pei Z., Tan H., Gu J., Lu L., Zeng X., Zhang T., Wang C., Ding L., Cullen P. J., Chen Z., Zhao S. (2023). A Polymeric Hydrogel Electrocatalyst for Direct Water Oxidation. Nat. Commun..

[cit9] Seitz L. C., Dickens C. F., Nishio K., Hikita Y., Montoya J., Doyle A., Kirk C., Vojvodic A., Hwang H. Y., Norskov J. K., Jaramillo T. F. (2016). A Highly Active and Stable IrO_*x*_/SrIrO_3_ Catalyst for the Oxygen Evolution Reaction. Science.

[cit10] Pi Y., Shao Q., Wang P., Guo J., Huang X. (2017). General Formation of Monodisperse IrM (M = Ni, Co, Fe) Bimetallic Nanoclusters as Bifunctional Electrocatalysts for Acidic Overall Water Splitting. Adv. Funct. Mater..

[cit11] Lin Y., Tian Z., Zhang L., Ma J., Jiang Z., Deibert B. J., Ge R., Chen L. (2019). Chromium-Ruthenium Oxide Solid Solution Electrocatalyst for Highly Efficient Oxygen Evolution Reaction in Acidic Media. Nat. Commun..

[cit12] Yao Y., Hu S., Chen W., Huang Z.-Q., Wei W., Yao T., Liu R., Zang K., Wang X., Wu G., Yuan W., Yuan T., Zhu B., Liu W., Li Z., He D., Xue Z., Wang Y., Zheng X., Dong J., Chang C.-R., Chen Y., Hong X., Luo J., Wei S., Li W.-X., Strasser P., Wu Y., Li Y. (2019). Engineering the Electronic Structure of Single Atom Ru Sites via Compressive Strain Boosts Acidic Water Oxidation Electrocatalysis. Nat. Catal..

[cit13] Zhai P., Xia M., Wu Y., Zhang G., Gao J., Zhang B., Cao S., Zhang Y., Li Z., Fan Z., Wang C., Zhang X., Miller J. T., Sun L., Hou J. (2021). Engineering Single-Atomic Ruthenium Catalytic Sites on Defective Nickel-Iron Layered Double Hydroxide for Overall Water Splitting. Nat. Commun..

[cit14] Macchioni A. (2019). The Middle-Earth between Homogeneous and Heterogeneous Catalysis in Water Oxidation with Iridium. Eur. J. Inorg. Chem..

[cit15] Fagiolari L., Bini M., Costantino F., Gatto G., Kropf A. J., Marmottini F., Nocchetti M., Wegener E. C., Zaccaria F., Delferro M., Vivani R., Macchioni A. (2020). Iridium-Doped Nanosized Zn–Al Layered Double Hydroxides as Efficient Water Oxidation Catalysts. ACS Appl. Mater. Interfaces.

[cit16] Fagiolari L., Zaccaria F., Costantino F., Vivani R., Mavrokefalos C. K., Patzke G. R., Macchioni A. (2020). Ir- and Ru-Doped Layered Double Hydroxides as Affordable Heterogeneous Catalysts for Electrochemical Water Oxidation. Dalton Trans..

[cit17] Li W., Feng B., Yi L., Li J., Hu W. (2021). Highly Efficient Alkaline Water Splitting with Ru-Doped Co−V Layered Double Hydroxide Nanosheets as a Bifunctional Electrocatalyst. ChemSusChem.

[cit18] Li Z., Liu D., Lu X., Du M., Chen Z., Teng J., Sha R., Tian L. (2022). Boosting Oxygen Evolution of Layered Double Hydroxide through Electronic Coupling with Ultralow Noble Metal Doping. Dalton Trans..

[cit19] Zhang S., Tan C., Yan R., Zou X., Hu F., Mi Y., Yan C., Zhao S. (2023). Constructing Built-in Electric Field in Heterogeneous Nanowire Arrays for Efficient Overall Water Electrolysis. Angew. Chem., Int. Ed..

[cit20] McCrory C. C. L., Jung S., Peters J. C., Jaramillo T. F. (2013). Benchmarking Heterogeneous Electrocatalysts for the Oxygen Evolution Reaction. J. Am. Chem. Soc..

[cit21] Yu M., Budiyanto E., Tüysüz H. (2022). Principles of Water Electrolysis and Recent Progress in Cobalt-, Nickel-, and Iron-Based Oxides for the Oxygen Evolution Reaction. Angew. Chem., Int. Ed..

[cit22] Zhang J., Liu J., Xi L., Yu Y., Chen N., Sun S., Wang W., Lange K. M., Zhang B. (2018). Single-Atom Au/NiFe Layered Double Hydroxide Electrocatalyst: Probing the Origin of Activity for Oxygen Evolution Reaction. J. Am. Chem. Soc..

[cit23] Liu K., Wang F., He P., Shifa T. A., Wang Z., Cheng Z., Zhan X., He J. (2018). The Role of Active Oxide Species for Electrochemical Water Oxidation on the Surface of 3d-Metal Phosphides. Adv. Energy Mater..

[cit24] Li Z., Niu W., Zhou L., Yang Y. (2018). Phosphorus and Aluminum Codoped Porous NiO Nanosheets as Highly Efficient Electrocatalysts for Overall Water Splitting. ACS Energy Lett..

[cit25] Wu Z., Liu X., Li H., Sun Z., Cao M., Li Z., Fang C., Zhou J., Cao C., Dong J., Zhao S., Chen Z. (2023). A Semiconductor-Electrocatalyst Nano Interface Constructed for Successive Photoelectrochemical Water Oxidation. Nat. Commun..

[cit26] Man I. C., Su H., Calle-Vallejo F., Hansen H. A., Martínez J. I., Inoglu N. G., Kitchin J., Jaramillo T. F., Nørskov J. K., Rossmeisl J. (2011). Universality in Oxygen Evolution Electrocatalysis on Oxide Surfaces. ChemCatChem.

[cit27] Craig M. J., Coulter G., Dolan E., Soriano-López J., Mates-Torres E., Schmitt W., García-Melchor M. (2019). Universal Scaling Relations for the Rational Design of Molecular Water Oxidation Catalysts with Near-Zero Overpotential. Nat. Commun..

[cit28] Pérez-Ramírez J., López N. (2019). Strategies to Break Linear Scaling Relationships. Nat. Catal..

[cit29] Haase F. T., Bergmann A., Jones T. E., Timoshenko J., Herzog A., Jeon H. S., Rettenmaier C., Cuenya B. R. (2022). Size Effects and Active State Formation of Cobalt Oxide Nanoparticles during the Oxygen Evolution Reaction. Nat. Energy.

[cit30] Lu M., Zheng Y., Hu Y., Huang B., Ji D., Sun M., Li J., Peng Y., Si R., Xi P., Yan C.-H. (2022). Artificially Steering Electrocatalytic Oxygen Evolution Reaction Mechanism by Regulating Oxygen Defect Contents in Perovskites. Sci. Adv..

[cit31] Cheng W., Xi S., Wu Z.-P., Luan D., (David) Lou X. W. (2021). In Situ Activation of Br-Confined Ni-Based Metal-Organic Framework Hollow Prisms toward Efficient Electrochemical Oxygen Evolution. Sci. Adv..

[cit32] Wu Z.-P., Zhang H., Zuo S., Wang Y., Zhang S. L., Zhang J., Zang S.-Q., (David) Lou X. W. (2021). Manipulating the Local Coordination and Electronic Structures for Efficient Electrocatalytic Oxygen Evolution. Adv. Mater..

[cit33] Hammes-Schiffer S., Galli G. (2021). Integration of Theory and Experiment in the Modelling of Heterogeneous Electrocatalysis. Nat. Energy.

[cit34] Song F., Bai L., Moysiadou A., Lee S., Hu C., Liardet L., Hu X. (2018). Transition Metal Oxides as Electrocatalysts for the Oxygen Evolution Reaction in Alkaline Solutions: An Application-Inspired Renaissance. J. Am. Chem. Soc..

[cit35] Rao R. R., Corby S., Bucci A., García-Tecedor M., Mesa C. A., Rossmeisl J., Giménez S., Lloret-Fillol J., Stephens I. E. L., Durrant J. R. (2022). Spectroelectrochemical Analysis of the Water Oxidation Mechanism on Doped Nickel Oxides. J. Am. Chem. Soc..

[cit36] Barlocco I., Cipriano L. A., Di Liberto G., Pacchioni G. (2023). Modeling Hydrogen and Oxygen Evolution Reactions on Single Atom Catalysts with Density Functional Theory: Role of the Functional. Adv. Theory Simul..

[cit37] Di Liberto G., Cipriano L. A., Pacchioni G. (2022). Universal Principles for the Rational Design of Single Atom Electrocatalysts? Handle with Care. ACS Catal..

[cit38] Mavros M. G., Tsuchimochi T., Kowalczyk T., McIsaac A., Wang L.-P., Voorhis T. V. (2014). What Can Density Functional Theory Tell Us about Artificial Catalytic Water Splitting?. Inorg. Chem..

[cit39] Mom R. V., Cheng J., Koper M. T. M., Sprik M. (2014). Modeling the Oxygen Evolution Reaction on Metal Oxides: The Influence of Unrestricted DFT Calculations. J. Phys. Chem. C.

[cit40] Seh Z. W., Kibsgaard J., Dickens C. F., Chorkendorff I., Nørskov J. K., Jaramillo T. F. (2017). Combining Theory and Experiment in Electrocatalysis: Insights into Materials Design. Science.

[cit41] Resasco J., Abild-Pedersen F., Hahn C., Bao Z., Koper M. T. M., Jaramillo T. F. (2022). Enhancing the Connection between Computation and Experiments in Electrocatalysis. Nat. Catal..

[cit42] Suntivich J., May K. J., Gasteiger H. A., Goodenough J. B., Shao-Horn Y. (2011). A Perovskite Oxide Optimized for Oxygen Evolution Catalysis from Molecular Orbital Principles. Science.

[cit43] Wang H., Zhang K. H. L., Hofmann J. P., de la Peña O'Shea V. A., Oropeza F. E. (2021). The Electronic Structure of Transition Metal Oxides for Oxygen Evolution Reaction. J. Mater. Chem. A.

[cit44] Sun Z., Lin L., He J., Ding D., Wang T., Li J., Li M., Liu Y., Li Y., Yuan M., Huang B., Li H., Sun G. (2022). Regulating the Spin State of Fe^III^ Enhances the Magnetic Effect of the Molecular Catalysis Mechanism. J. Am. Chem. Soc..

[cit45] Duan Z., Henkelman G. (2020). Surface Charge and Electrostatic Spin Crossover Effects in CoN_4_ Electrocatalysts. ACS Catal..

[cit46] Yang C., Nikiforidis G., Park J. Y., Choi J., Luo Y., Zhang L., Wang S.-C., Chan Y.-T., Lim J., Hou Z., Baik M.-H., Lee Y., Byon H. R. (2018). Designing Redox-Stable Cobalt–Polypyridyl Complexes for Redox Flow Batteries: Spin-Crossover Delocalizes Excess Charge. Adv. Energy Mater..

[cit47] Hegner F. S., Galán-Mascarós J. R., López N. (2022). Lowering the Water Oxidation Overpotential by Spin-Crossover in Cobalt Hexacyanoferrate. J. Phys. Chem. Lett..

[cit48] Zhao S., Wang Y., Dong J., He C.-T., Yin H., An P., Zhao K., Zhang X., Gao C., Zhang L., Lv J., Wang J., Zhang J., Khattak A. M., Khan N. A., Wei Z., Zhang J., Liu S., Zhao H., Tang Z. (2016). Ultrathin Metal–Organic Framework Nanosheets for Electrocatalytic Oxygen Evolution. Nat. Energy.

[cit49] Pintado S., Goberna-Ferrón S., Escudero-Adán E. C., Galán-Mascarós J. R. (2013). Fast and Persistent Electrocatalytic Water Oxidation by Co–Fe Prussian Blue Coordination Polymers. J. Am. Chem. Soc..

[cit50] Wang X.-L., Dong L.-Z., Qiao M., Tang Y.-J., Liu J., Li Y., Li S.-L., Su J.-X., Lan Y.-Q. (2018). Exploring the Performance Improvement of the Oxygen Evolution Reaction in a Stable Bimetal–Organic Framework System. Angew. Chem., Int. Ed..

[cit51] Hai Y., Liu L., Gong Y. (2021). Iron Coordination Polymer, Fe(Oxalate)(H_2_O)_2_ Nanorods Grown on Nickel Foam via One-Step Electrodeposition as an Efficient Electrocatalyst for Oxygen Evolution Reaction. Inorg. Chem..

[cit52] Biradha K., Goswami A., Moi R. (2020). Coordination Polymers as Heterogeneous Catalysts in Hydrogen Evolution and Oxygen Evolution Reactions. Chem. Commun..

[cit53] Menendez Rodriguez G., Macchioni A. (2023). Metal Doping and Ligand Engineering as Tools for Tailoring the Electronic Structure of Coordination Polymers and Their Oxygen Evolution Electrocatalytic Activity. Eur. J. Inorg. Chem..

[cit54] Zhou J., Han Z., Wang X., Gai H., Chen Z., Guo T., Hou X., Xu L., Hu X., Huang M., Levchenko S. V., Jiang H. (2021). Discovery of Quantitative Electronic Structure-OER Activity Relationship in Metal-Organic Framework Electrocatalysts Using an Integrated Theoretical-Experimental Approach. Adv. Funct. Mater..

[cit55] Galli S., Tagliabue G., Masciocchi N., Wang W. G., Barea E., Navarro J. A. R. (2011). From 1D Homoleptic to 2D Heteroleptic Pillared Coordination Polymers Containing Oxonato Bridges. Inorg. Chim. Acta.

[cit56] Goswami S., Biswas S., Konar S. (2015). Concomitant Spin-Canted Antiferromagnetic Ordering and Proton Conduction in Homometallic and Homoleptic Coordination Polymers. Dalton Trans..

[cit57] Wan W., Zhao Y., Wei S., Triana C. A., Li J., Arcifa A., Allen C. S., Cao R., Patzke G. R. (2021). Mechanistic Insight into the Active Centers of Single/Dual-Atom Ni/Fe-Based Oxygen Electrocatalysts. Nat. Commun..

[cit58] Liang Q., Brocks G., Bieberle-Hütter A. (2021). Oxygen Evolution Reaction (OER) Mechanism under Alkaline and Acidic Conditions. JPhys Energy.

[cit59] Ortuño M. A., Bernales V., Gagliardi L., Cramer C. J. (2016). Computational Study of First-Row Transition Metals Supported on MOF NU-1000 for Catalytic Acceptorless Alcohol Dehydrogenation. J. Phys. Chem. C.

[cit60] Neese F. (2012). The ORCA Program System. Wiley Interdiscip. Rev.: Comput. Mol. Sci..

[cit61] Grimme S., Antony J., Ehrlich S., Krieg H. (2010). A Consistent and Accurate *Ab Initio* Parametrization of Density Functional Dispersion Correction (DFT-D) for the 94 Elements H-Pu. J. Chem. Phys..

[cit62] Grimme S. (2011). Density Functional Theory with London Dispersion Corrections. Wiley Interdiscip. Rev.: Comput. Mol. Sci..

[cit63] Weigend F., Ahlrichs R. (2005). Balanced Basis Sets of Split Valence, Triple Zeta Valence and Quadruple Zeta Valence Quality for H to Rn: Design and Assessment of Accuracy. Phys. Chem. Chem. Phys..

[cit64] Cossi M., Rega N., Scalmani G., Barone V. (2003). Energies, Structures, and Electronic Properties of Molecules in Solution with the C-PCM Solvation Model. J. Comput. Chem..

[cit65] Du J., Li F., Sun L. (2021). Metal–Organic Frameworks and Their Derivatives as Electrocatalysts for the Oxygen Evolution Reaction. Chem. Soc. Rev..

[cit66] Friebel D., Louie M. W., Bajdich M., Sanwald K. E., Cai Y., Wise A. M., Cheng M.-J., Sokaras D., Weng T.-C., Alonso-Mori R., Davis R. C., Bargar J. R., Nørskov J. K., Nilsson A., Bell A. T. (2015). Identification of Highly Active Fe Sites in (Ni,Fe)OOH for Electrocatalytic Water Splitting. J. Am. Chem. Soc..

[cit67] Deabate S., Fourgeot F., Henn F. (2000). X-Ray Diffraction and Micro-Raman Spectroscopy Analysis of New Nickel Hydroxide Obtained by Electrodialysis. J. Power Sources.

[cit68] Yeo B. S., Bell A. T. (2012). In Situ Raman Study of Nickel Oxide and Gold-Supported Nickel Oxide Catalysts for the Electrochemical Evolution of Oxygen. J. Phys. Chem. C.

[cit69] Chen Z., Jiang S., Kang G., Nguyen D., Schatz G. C., Van Duyne R. P. (2019). Operando Characterization of Iron Phthalocyanine Deactivation during Oxygen Reduction Reaction Using Electrochemical Tip-Enhanced Raman Spectroscopy. J. Am. Chem. Soc..

[cit70] Zhao K., Liu S., Li Y., Wei X., Ye G., Zhu W., Su Y., Wang J., Liu H., He Z., Zhou Z., Sun S. (2022). Insight into the Mechanism of Axial Ligands Regulating the Catalytic Activity of Fe–N_4_ Sites for Oxygen Reduction Reaction. Adv. Energy Mater..

[cit71] Wei J., Xia D., Wei Y., Zhu X., Li J., Gan L. (2022). Probing the Oxygen Reduction Reaction Intermediates and Dynamic Active Site Structures of Molecular and Pyrolyzed Fe–N–C Electrocatalysts by In Situ Raman Spectroscopy. ACS Catal..

[cit72] Görlin M., Ferreira de Araújo J., Schmies H., Bernsmeier D., Dresp S., Gliech M., Jusys Z., Chernev P., Kraehnert R., Dau H., Strasser P. (2017). Tracking Catalyst Redox States and Reaction Dynamics in Ni–Fe Oxyhydroxide Oxygen Evolution Reaction Electrocatalysts: The Role of Catalyst Support and Electrolyte pH. J. Am. Chem. Soc..

[cit73] Abild-Pedersen F., Greeley J., Studt F., Rossmeisl J., Munter T. R., Moses P. G., Skúlason E., Bligaard T., Nørskov J. K. (2007). Scaling Properties of Adsorption Energies for Hydrogen-Containing Molecules on Transition-Metal Surfaces. Phys. Rev. Lett..

