# Supplementary material for: Harnessing the electronic structure of active metals to lower the overpotential of the electrocatalytic oxygen evolution reaction
Source: Chem Sci. 2023 Dec 12;15(4):1348–63. doi: 10.1039/d3sc05891c (PMC10806668; doi:10.1039/d3sc05891c)
Supplement: SC-015-D3SC05891C-s001 [file SC-015-D3SC05891C-s001.pdf]

Electronic Supplementary Material (ESI) for Chemical Science.
This journal is © The Royal Society of Chemistry 2023

Electronic Supporting Information (ESI)

Harnessing the Electronic Structure of the Active Metal to Lower the Overpotential of the Electrocatalytic Oxygen Evolution Reaction

Lorenzo Baldinelli,[a] Gabriel Menendez Rodriguez,*[a] Iolanda D'Ambrosio,[a] Amalia Malina Grigoras,[a] Riccardo Vivani,[b] Loredana Latterini,[a] Alceo Macchioni,[a] Filippo De Angelis,[a,c,d,e] Giovanni Bistoni*[a]

[a]*Dipartmento di Chimica, Biologia e Biotecnologie, Università Degli Studi Di Perugia, Via Elce di sotto, 8, 06123 Perugia (Italy);*

[b]*Dipartimento di Scienze Farmaceutiche, Università Degli Studi Di Perugia, Via del Liceo, 06123 Perugia (Italy)*;

[c]*Computational Laboratory for Hybrid/ Organic Photovoltaics (CLHYO), Istituto CNR di Scienze e Tecnologie Chimiche "Giulio Natta" (CNR-SCITEC), 06123 Perugia, Italy;*

[d]*Department of Mechanical Engineering, College of Engineering, Prince Mohammad Bin Fahd University, Al Khobar 31952, Saudi Arabia;*

[e]*SKKU Institute of Energy Science and Technology (SIEST), Sungkyunkwan University, Suwon 440-746, Korea;*

gabriel.menendezrodriguez@unipg.it.; giovanni.bistoni@unipg.it

Table of Contents

I. Experimental Part 3
S1 Experimental procedures 3
S2 Powder X-Ray Diffraction 3
S3 Synthesis of CPs 5
S4 Electrochemical measurements 6
S5 Working electrode preparation 7
S6 Reference electrode calibration 8
S7 Stability evaluation via Raman, SEM and EDX analysis 13
S8 Reflectance spectra 16
II. Computational Part 17
S9 Computational protocol 17
S10 Method and model validation 18
S11 Ground state electronic structure 21
S12 Validation of the ground state spin configuration 26
S13 Electronic structure characterization 30
S14 Electronic transitions characterization 33
S15 OER reaction free energies in alkaline conditions 39
S16 Reaction intermediates analysis 40
S17 OER intermediates 46
S18 Structures 46
III. References 70

I. Experimental Part

S1 Experimental procedures

Nickel(II) perchlorate hexahydrate ($Ni(ClO_4)_2$ x6H_2O), Cobalt(II) chloride hexahydrate ($CoCl_2$ x6H_2O), Iron(II) perchlorate hydrate ($Fe(ClO_4)_2$ xH_2O), Manganese(II) chloride tetrahydrate ($MnCl_2$ x4H_2O) and 4,6-Dihydroxy-1,3,5-triazine-2-carboxylic acid potassium salt (Oxonic acid potassium salt) were purchased from Sigma-Aldrich and used as received. Distilled water was further purified using a Milli-Q Ultrapure water purification system. Metal analyses were performed with a Varian 700-ES series inductively coupled plasma-optical emission spectrometer (ICP-OES).

S2 Powder X-Ray Diffraction

Powder X-ray diffraction (PXRD) patterns were collected with the Cu-Kα radiation on a Bruker D8 Advance diffractometer, equipped with a Lynxeye XE-T detector. The long fine focus (LFF) tube was operated at 40 kV and 40 mA. To minimize preferred orientations of the microcrystals, the samples were carefully side-loaded onto a zero-background sample holder. Unit cell parameters were determined by the N-Treor program operating within the EXPO2014 software.[1] Indication of possible space groups was obtained by the same EXPO software, which confirmed those found in the literature. Rietveld refinement procedures were performed with the help of the GSAS-II package.[2] The refinement involved unit cell parameters, positional atomic coordinates, background, and peak profile parameters. Atomic displacement factors were set to the literature values and were not refined. Soft constraints were adopted for light atoms to avoid unrealistic bond distances and angles. The statistical weight of these constraints was slowly decreased during the refinement procedure. Atomic positions and site occupancy factors of water molecules were not refined. At the end of the refinement, the shifts in all parameters were less than their standard deviations. **Table S1** resumes the main crystallographic data of the analyzed samples, while **Figure S1** shows the related final Rietveld plots.

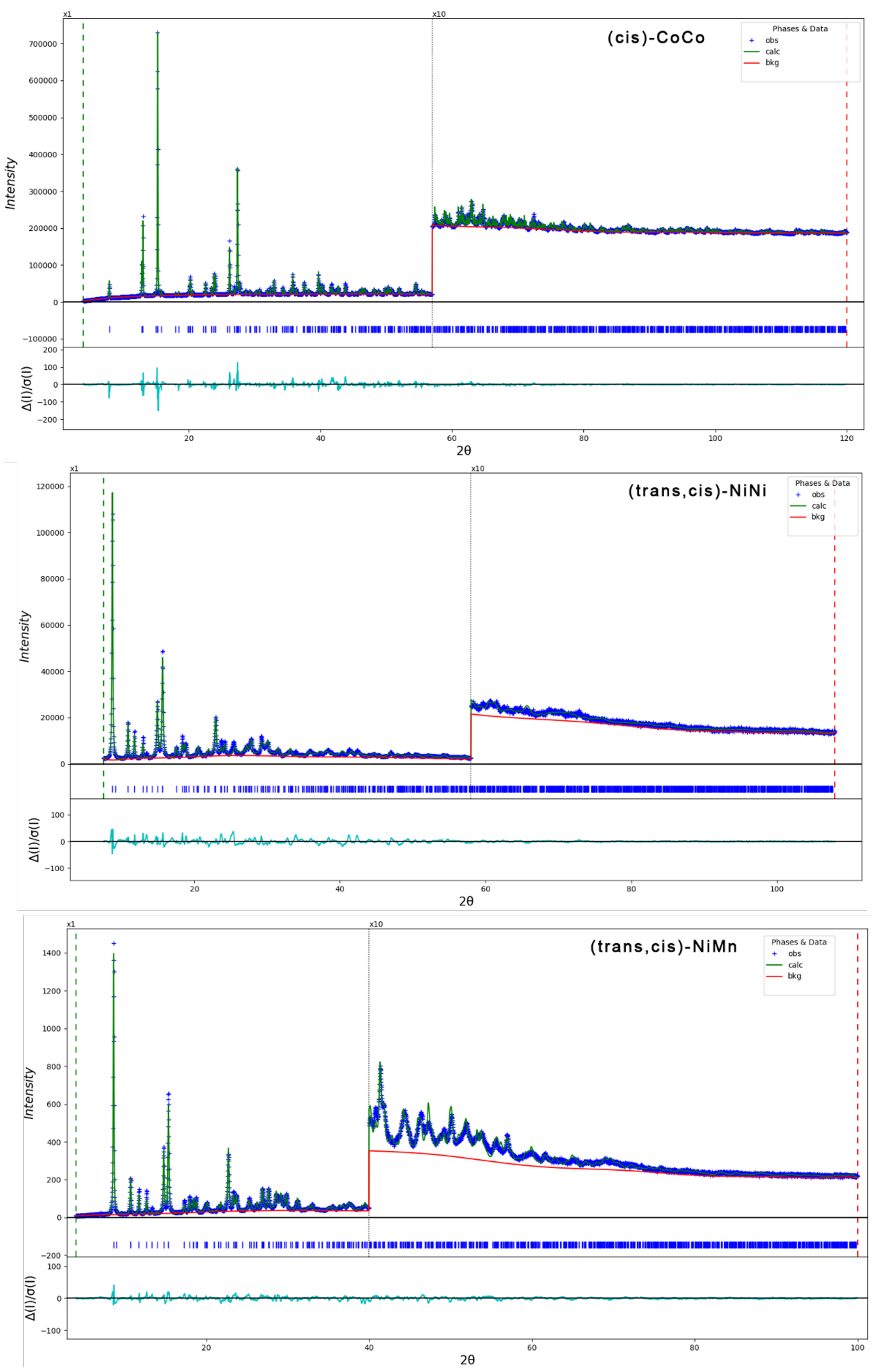

Figure S1. Final Rietveld plots for **(cis)-CoCo**, **(trans,cis)-NiNi** and **(trans,cis)-NiMn**, reporting the observed pattern (blue symbols), the calculated pattern (green line), and their difference (cyan line). Blue markers at the bottom indicate the calculated positions of peaks.

Table S1. Main crystallographic data of the analyzed samples. The structures of all samples were found to have a tetragonal symmetry.

CP	sp.gr.	a (Å)	c (Å)	V (Å^3)	R_{wp} [(b)]	χ^2 [(c)]	M_{20} [(d)]
**(cis)-CoCo**[(a)]		13.6281(2)	19.7905(4)	3675.6(1)	0.073	10.9	
**(cis)-CoFe**		13.625(1)	19.826(3)	3680.4(5)			21
**(cis)-CoNi**	I4$_1$/a	13.659(1)	19.635(2)	3663.3(6)			29
**(cis)-FeMn**		13.614(1)	20.248(2)	3752.9(6)			34
**(trans,cis)-NiNi**[(a)]		13.7020(1)	15.0717(1)	2829.6(3)	0.116	6.9	
**(trans,cis)-NiMn**[(a)]		13.950(1)	15.0621(7)	2930.9(5)	0.072	4.1	
**(trans,cis)-NiCo**	P4$_2$/n	13.781(3)	15.037(5)	2856(1)			17
**(trans,cis)-NiFe**		13.812(5)	15.021(4)	2865(1)			20

[(a)] refined with the Rietveld method; [(b)] $R_{wp} = [\Sigma\, w(Io\text{-}Ic)^2 / \Sigma\, wIo^2]^{1/2}$; [(c)] $\chi^2 = [\Sigma\, w(Io\text{-}Ic)^2 / (No - Nvar)]^{1/2}$; [(d)] $M_{20} = Q_{20}/(2N_{20}|\Delta Q|_{avg})^3$

S3 Synthesis of CPs

Homometallic **(trans,cis)-NiNi** and **(cis)-CoCo** were synthetized slightly modifying the procedure reported in the literature.[4] Oxonic acid potassium salt (0.50 mmol) was dissolved in 5 mL of water and heated at 70-80 ^{0}C under stirring. After its complete dissolution, $Ni(ClO_4)_2$ x6H_2O or $CoCl_2$ x6H_2O (0.50 mmol) was added to the solution and the resulting mixture was allowed to react for 4 hours. The resulting suspension was filtered; the collected powder was washed with deionized water, ethanol, diethyl ether and dried in air for 24 h. Heterobimetallic CPs were obtained with an analogous procedure, introducing

two metal salts in the suitable proportions. **Table S2** show the reagents used for the synthesis of heterobimetallic CPs and the molar fraction of each metal ion derived by ICP.

Table S2. Reagents and ICP data of the synthesized heterobimetallic CPs.

**CP**	**Metal salt (mmol)**		**ICP molar fraction**	
**(cis)-CoFe**	$CoCl_2$ $6H_2O$ (0.3)	$Fe(ClO_4)_2$ H_2O (0.2)	Co (0.87)	Fe (0.13)
**(cis)-CoMn**	$CoCl_2$ $6H_2O$ (0.2)	$MnCl_2$ $4H_2O$ (0.3)	Co (0.84)	Mn (0.16)
**(cis)-CoNi**	$CoCl_2$ $6H_2O$ (0.35)	$Ni(ClO_4)_2$ $6H_2O$ (0.15)	Co (0.68)	Ni (0.32)
**(cis)-FeMn**	$Fe(ClO_4)_2$ H_2O (0.2)	$MnCl_2$ $4H_2O$ (0.3)	Fe (0.78)	Mn (0.22)
**(trans,cis)-NiFe**	$Ni(ClO_4)_2$ $6H_2O$ (0.3)	$Fe(ClO_4)_2$ H_2O (0.2)	Ni (0.83)	Fe (0.17)
**(trans,cis)-NiCo**	$Ni(ClO_4)_2$ $6H_2O$ (0.35)	$CoCl_2$ $6H_2O$ (0.15)	Ni (0.76)	Co (0.24)
**(trans,cis)-NiMn**	$Ni(ClO_4)_2$ $6H_2O$ (0.2)	$MnCl_2$ $4H_2O$ (0.3)	Ni (0.86)	Mn (0.14)

S4 Electrochemical measurements

Electrochemical measurements were performed with a Squidstat Solo potentiostat (manufactured by Admiral Instruments) using a one-compartment three-electrode cell. In all experiments, a classical electrochemical cell with a capacity of 20.0 mL equipped with a Hg/HgO (1 M NaOH) reference electrode, a platinum wire as the counter electrode and a glassy carbon working electrode was used. All measurements were carried out at room temperature without stirring using a 1 M KOH solution as electrolyte without iR-correction. The potentials reported in the work were calibrated to reversible hydrogen electrode (RHE) according to the Nernst equation: E (V vs. RHE) = E (V vs. Hg/HgO) + 0.918 V. Cyclic voltammograms (CVs) were recorded with a scan rate of 100 mV/s whereas linear scan voltammetry (LSV) was performed at a scan rate of 5 mV/s. The long-term stability of CPs was assessed

by evaluating the current decrease at 1.9 V vs RHE after 50 cycles from 0.918 to 1.918 V at 100 mV/s. The electrochemically active surface area (ECSA) was calculated using the fitted value of the double-layer capacitance (Cdl) using the formula:

ECSA = Cdl/Cs

Cdl was determined by recording CVs at different scan rates (5 - 100 mV/s) in a non-Faradaic region and corresponds to one-half the slope of the linear fit of the capacitive current ($\Delta i = ia - ic$) versus scan rate plot. The specific capacitance of the electrolyte (Cs) was taken as 40 μF/cm^2.[5]

S5 Working electrode preparation

The working electrodes for the electrochemical studies were prepared as reported in Scheme S1. First, an electrocatalyst suspension was prepared by adding 1 mL of ethanol to 1 mg of CP. The resulting suspension was sonicated for 5 minutes. In parallel, a carbon nanotubes (CNTs) suspension was prepared by sonicating 5 mg of CNT in 5 mL of ethanol for 30 minutes. The electrocatalyst ink was obtained by mixing 0.1 mL of electrocatalyst suspension and 0.4 mL of CNTs suspension. Finally, 10 μL of electrocatalyst ink was carefully drop casted onto the glassy carbon working electrode and allowed to dry in air for 4 hours.

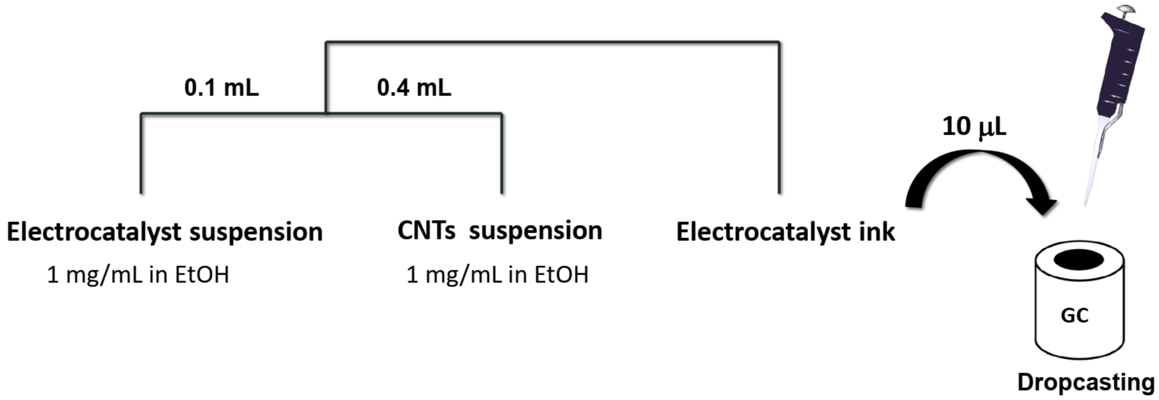

Scheme S1. Schematic representation of the working electrode preparation.

S6 Reference electrode calibration

The Hg/HgO (NaOH 1M) reference electrode was calibrated with respect to reversible hydrogen electrode (RHE) by measuring the open circuit potential (OCP) between a Pt/H_2 working electrode and the Hg/HgO (NaOH 1 M) reference electrode. Both electrodes were immersed in the electrolyte KOH solution at pH 14 and the OCP was measured for 10 minutes (**Figure S2**). The experiment was repeated three times and an average value of 0.918 V was obtained, which is close to the theoretical value calculated by the Nernst equation (0.926 V).

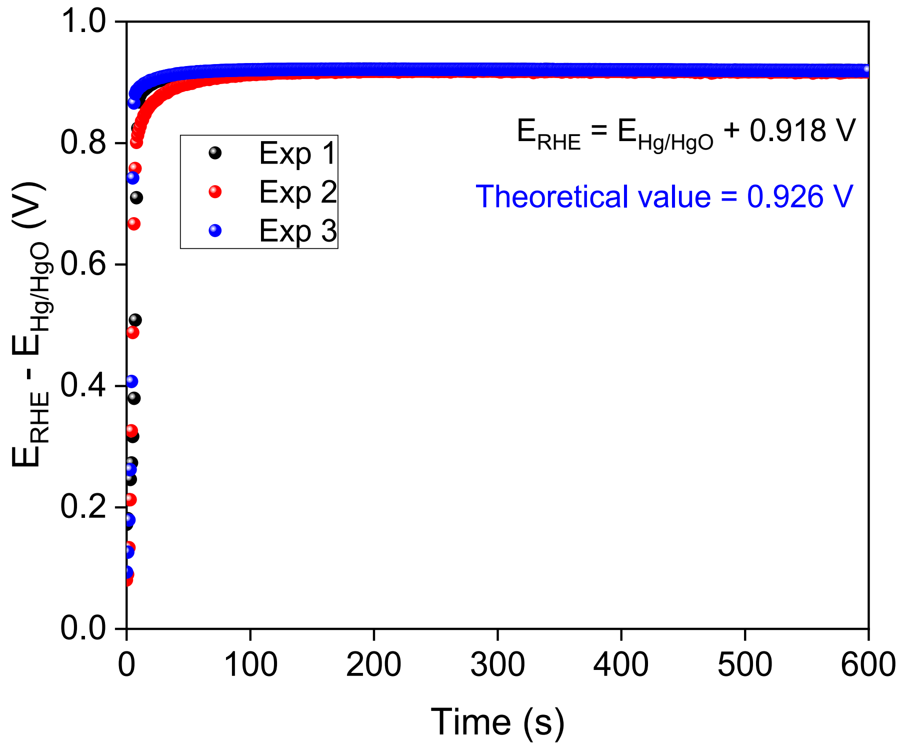

Figure S2. Calibration of Hg/HgO (NaOH 1M) in KOH electrolyte solution (pH = 14) with respect to RHE.

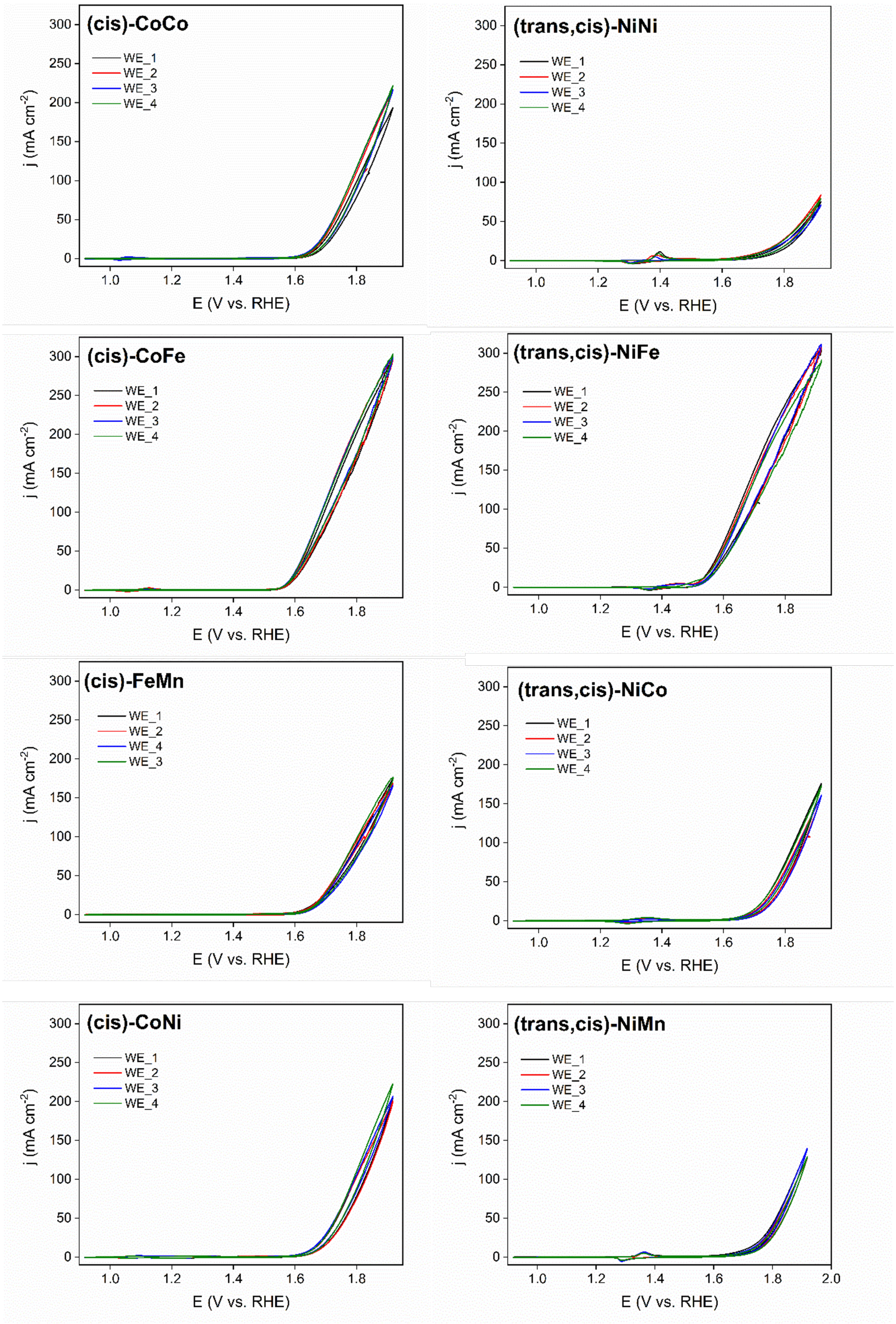

Figure S3. Comparison of four independently prepared working electrode (WEs) for each CP electrocatalytic system, recorded in 1 M KOH at a scan rate of 100 mV/s.

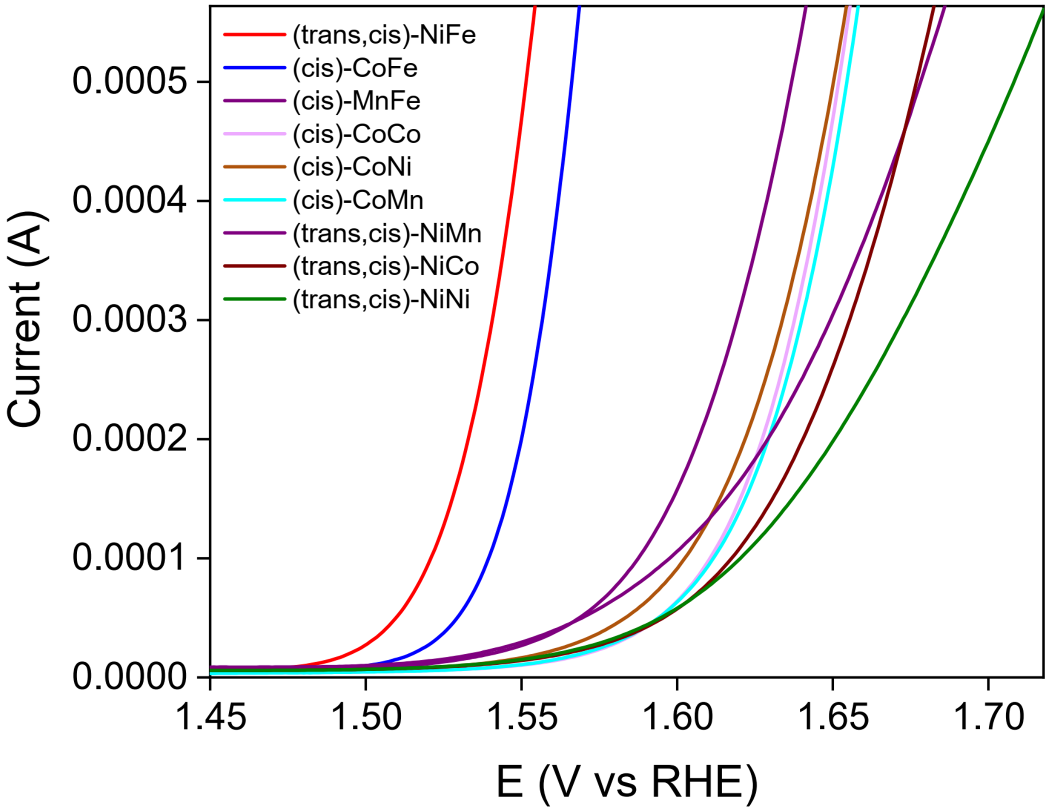

Figure S4. LSV curves of CPs recorded in 1 M KOH at a scan rate of 5 mV/s.

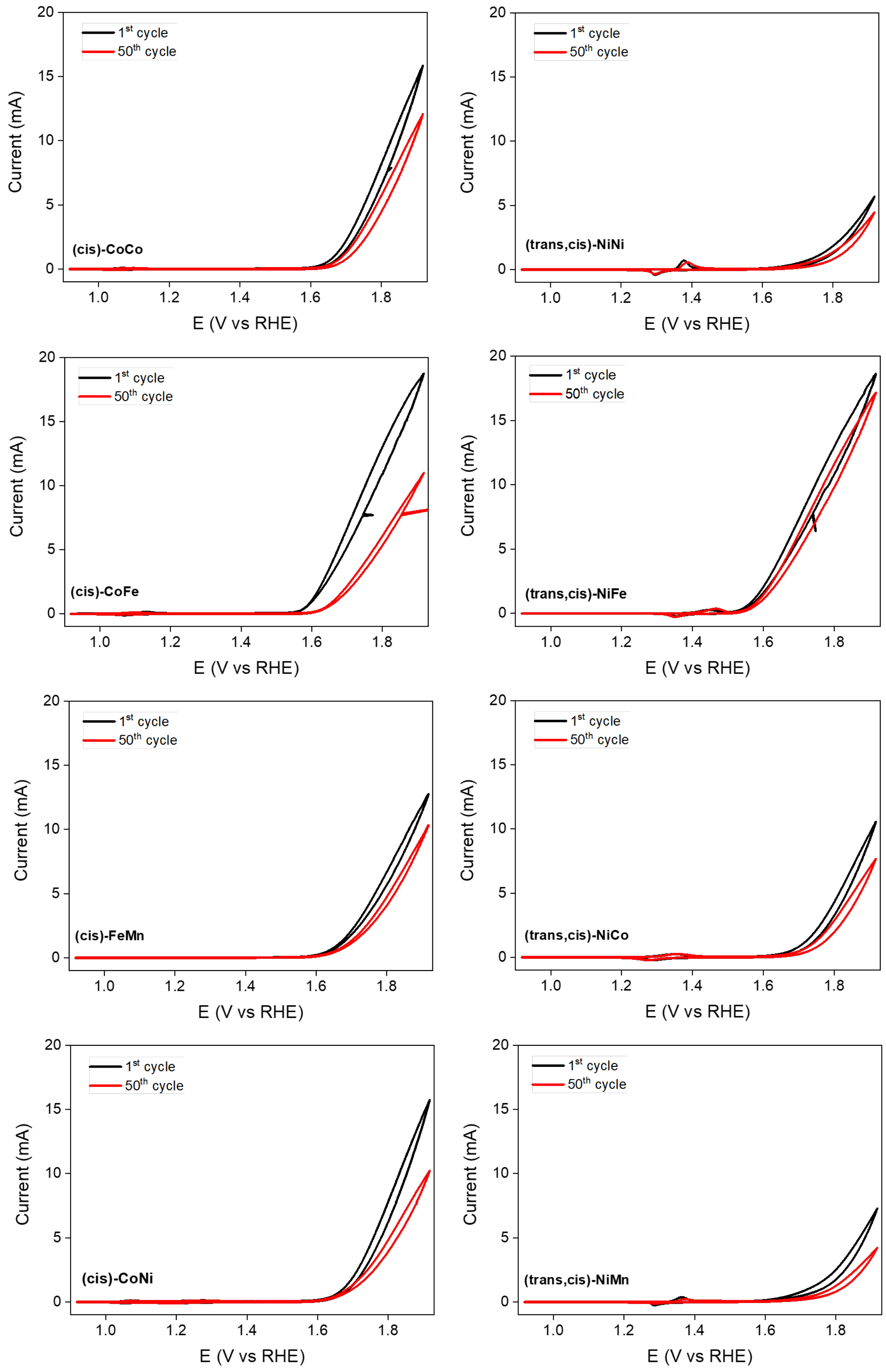

Figure S5. Comparison of the 1st (black) and 50th (red) cyclic voltammogram of CPs recorded in 1 M KOH at a scan rate of 100 mV/s. The gradual decrease in catalytic current indicates the limited stability of CPs under electrocatalytic conditions.

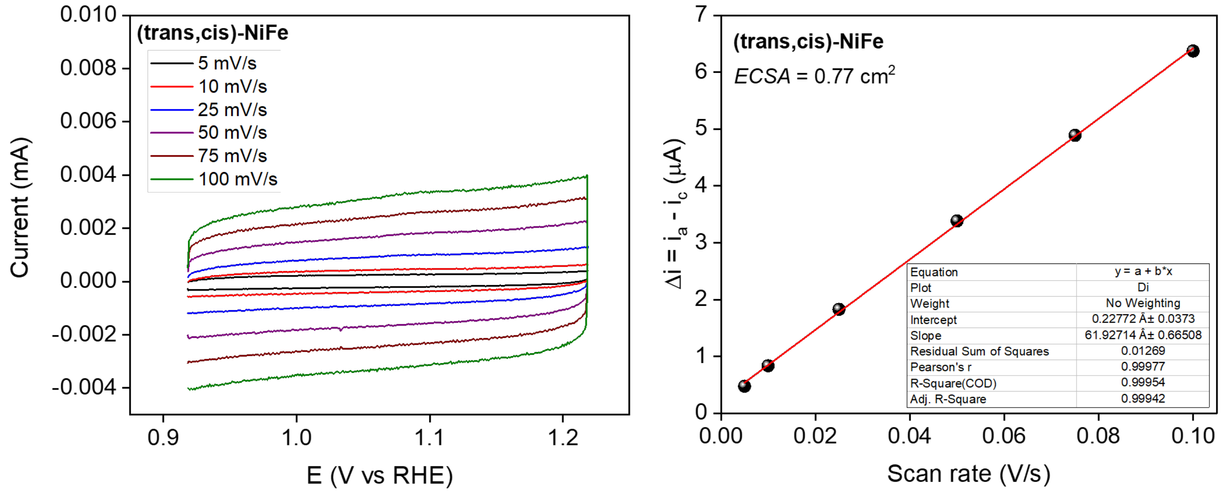

Figure S6. CVs of **(trans,cis)-NiFe** at the different scan rates from 5-100 mV/s (left) and the plot of the capacitive currents versus scan rate (right).

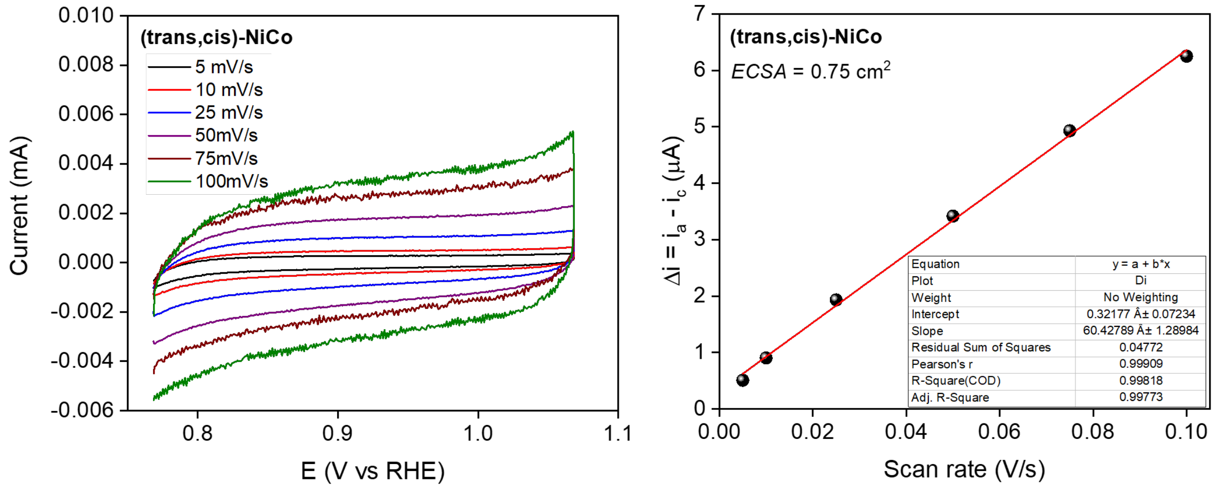

Figure S7. CVs of **(trans,cis)-NiCo** at the different scan rates from 5-100 mV/s (left) and the plot of the capacitive currents versus scan rate (right).

Note: As shown in **Figure S6Figure S7**, the ECSA determined for **(trans,cis)-NiFe** (0.77 cm^2) and **(trans,cis)-NiCo** (0.75 cm^2) is substantially the same, thus the huge difference of electrocatalytic performances between theses CPs can only be attributed to their different intrinsic activity and not to the accessibility of active sites to the medium/electrolyte.

S7 Stability evaluation via Raman, SEM and EDX analysis

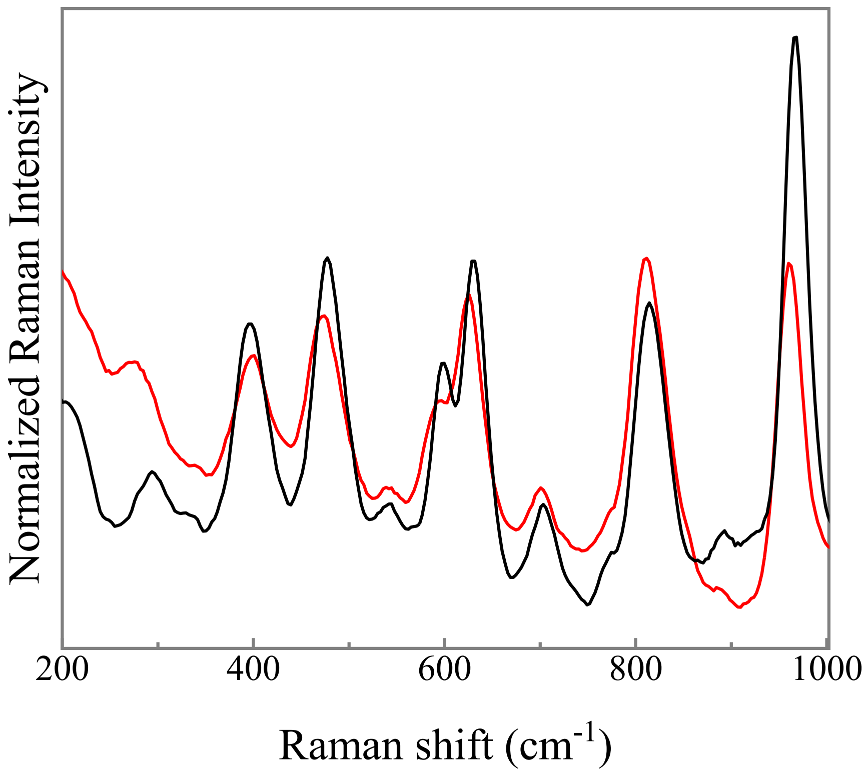

Figure S8. Raman spectra of **(trans,cis)-NiFe** (black line) and NTC matrix + **(trans,cis)-NiFe** untreated (red line).

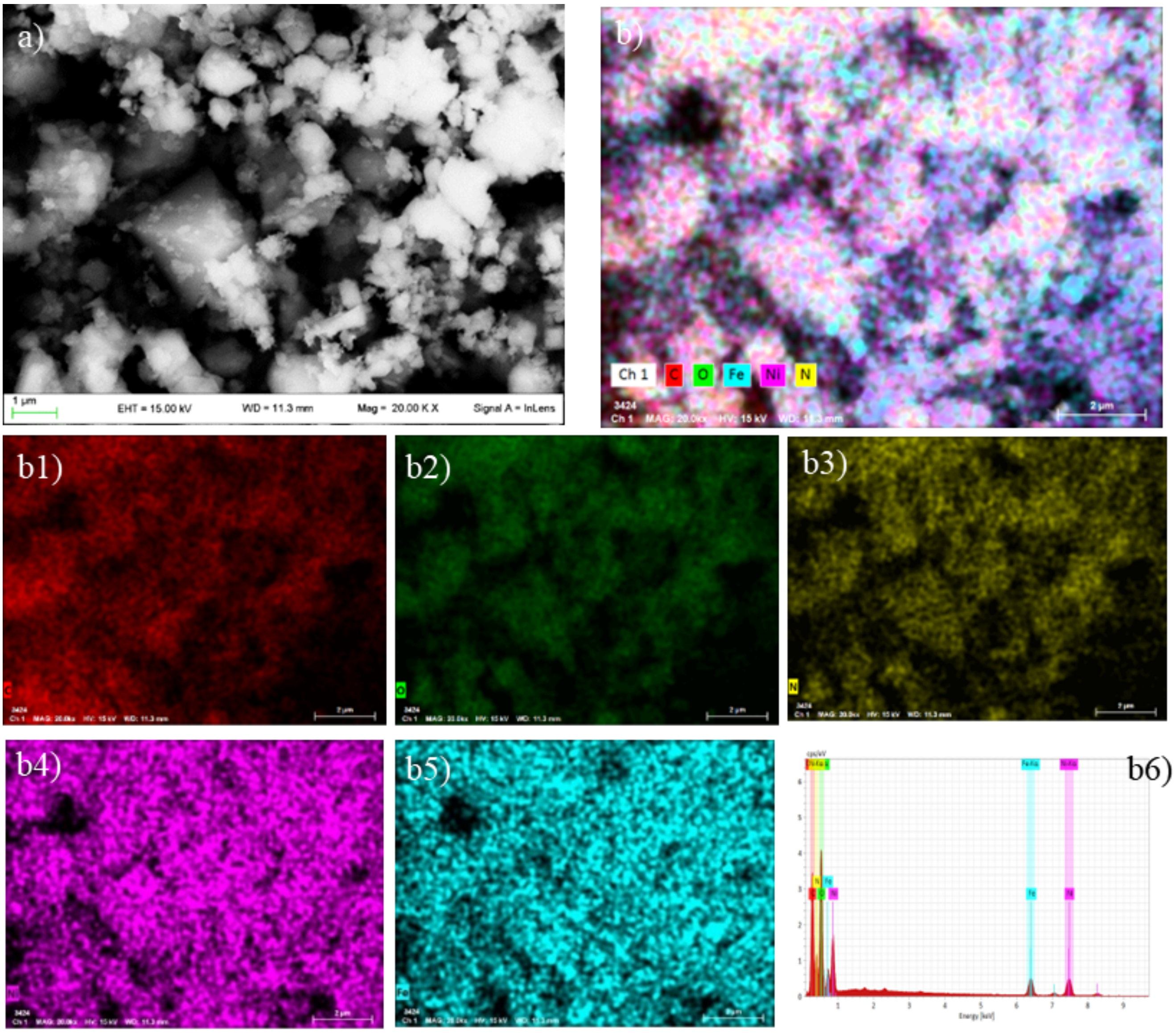

Figure S9. a) SEM image; b) EDX mapping and elemental analysis, including b1) C; b2) O; b3) N; b4) Ni; b5) Fe; b6) elemental analysis for **(trans,cis)-NiFe**.

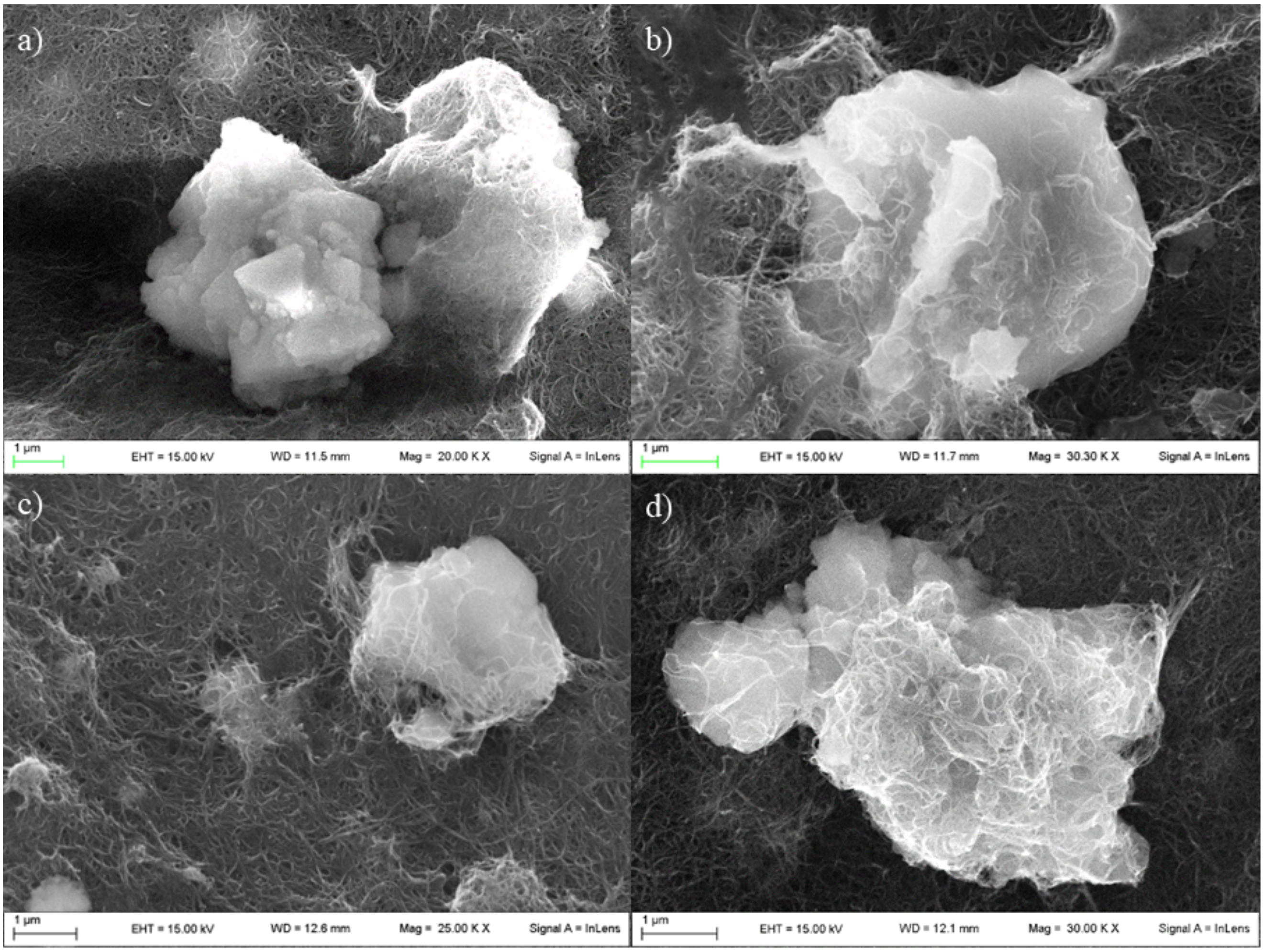

Figure S10. SEM images of a) NTC matrix + **(trans,cis)-NiFe** untreated; b) NTC matrix + **(trans,cis)-NiFe** treated with KOH; c) NTC matrix + **(trans,cis)-NiFe** treated with KOH after 1CV; NTC matrix + **(trans,cis)-NiFe** treated with KOH after 50 CVs.

Table S3. Observed Ni:Fe and M:O ratio based on EDX analysis.

Sample	Ni : Fe
**(trans,cis)-NiFe**	1 : 0.7
**NTC matrix + (trans,cis)-NiFe untreated**	1 : 0.4
**NTC matrix + (trans,cis)-NiFe 1M KOH 30s**	1 : 0.7
**NTC matrix + (trans,cis)-NiFe 1M KOH 30s 1CV**	1 : 0.4
**NTC matrix + (trans,cis)-NiFe 1M KOH 30s 50CV**	1 : 0.5

S8 Reflectance spectra

Diffuse reflectance recorded from the powder samples display different contributions from scattering process (evaluated from R% at 800 nm), which are likely due to different grain size of the samples.

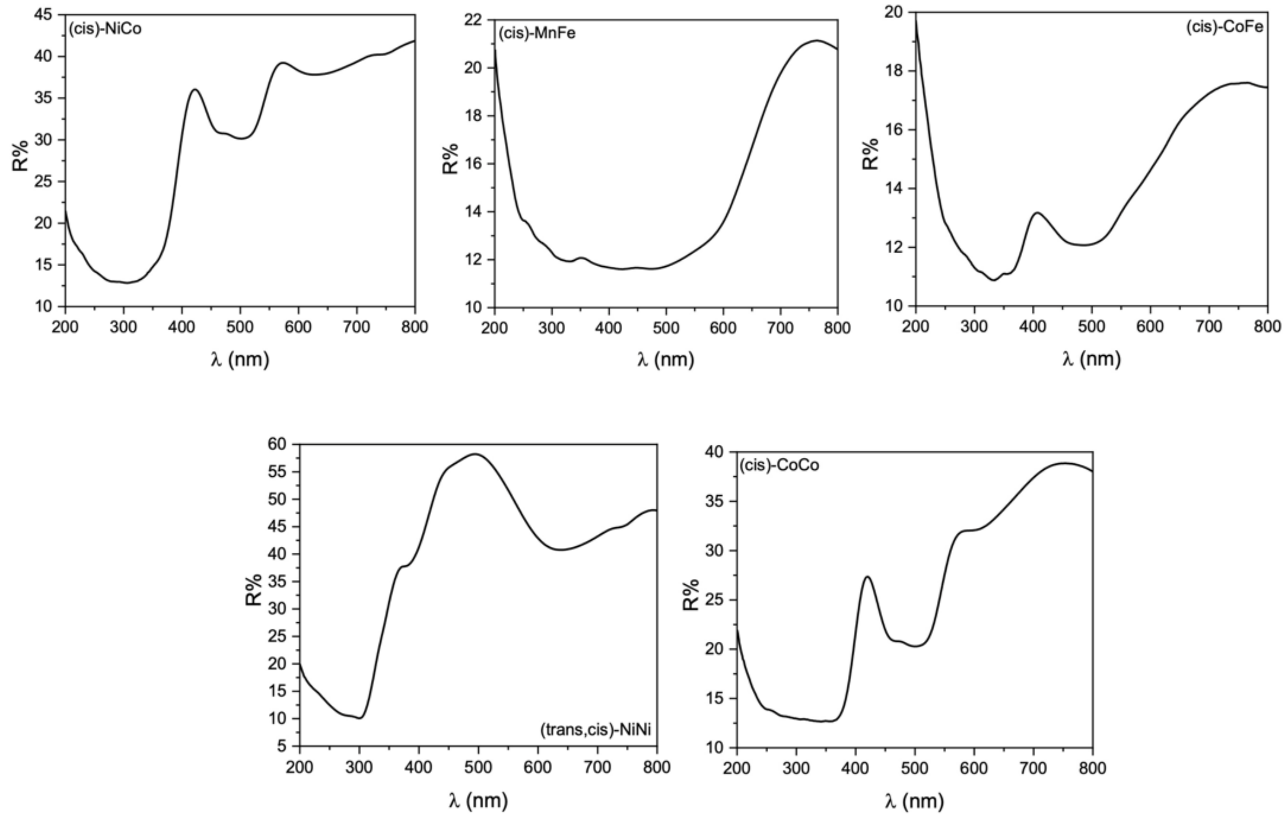

Figure S11. Reflectance spectra of the investigated samples.

II. Computational Part

S9 Computational protocol

All calculations were carried out using the ORCA quantum chemistry program package based v. 5.0.3.[6] Geometry optimizations were carried out using the B3LYP functional together with Grimme's D3 dispersion correction and Becke-Johnson damping.[7–12] The Ahlrichs def2-TZVP(-f) basis set was used for all the calculations, unless otherwise specified.[13] To model CPs, we defined a molecular cluster with two metal centers fully coordinated by water and oxonato ligands, in which the environment, i.e., the remaining of the system and (if present) the water solvent, is treated using an implicit solvation approach (C-PCM).[14,15] The dielectric constant of water was used as a model. This computational protocol is denoted hereafter as B3LYP-D3+C-PCM(Water)/def2-TZVP(-f). Excited states were calculated at the same level of theory within the TDDFT framework. Unless otherwise specified, the Tamm-Dancoff Approximation (TDA) was used in all TDDFT calculations. The default SCF convergence criteria and DFT grid settings (defgrid2) of ORCA v. 5.0.3 were used in all cases. For the Raman spectra the polarizability was computed analytically by solving the CP-SCF equations (POLAR 1). When two deprotonated water molecules bind to Fe, constraints were employed for the metals and the atoms with dangling bonds. In this case Partial Hessian Vibrational Analysis (PHVA) calculations were run.[16]

S10 Method and model validation

In this section we calibrate our computational protocol by comparing the experimental absorption spectra in the solid phase with the computed spectra on our model systems using various computational settings. **Figure S12Figure S15** show the spectra obtained for **(trans,cis)-NiNi**.

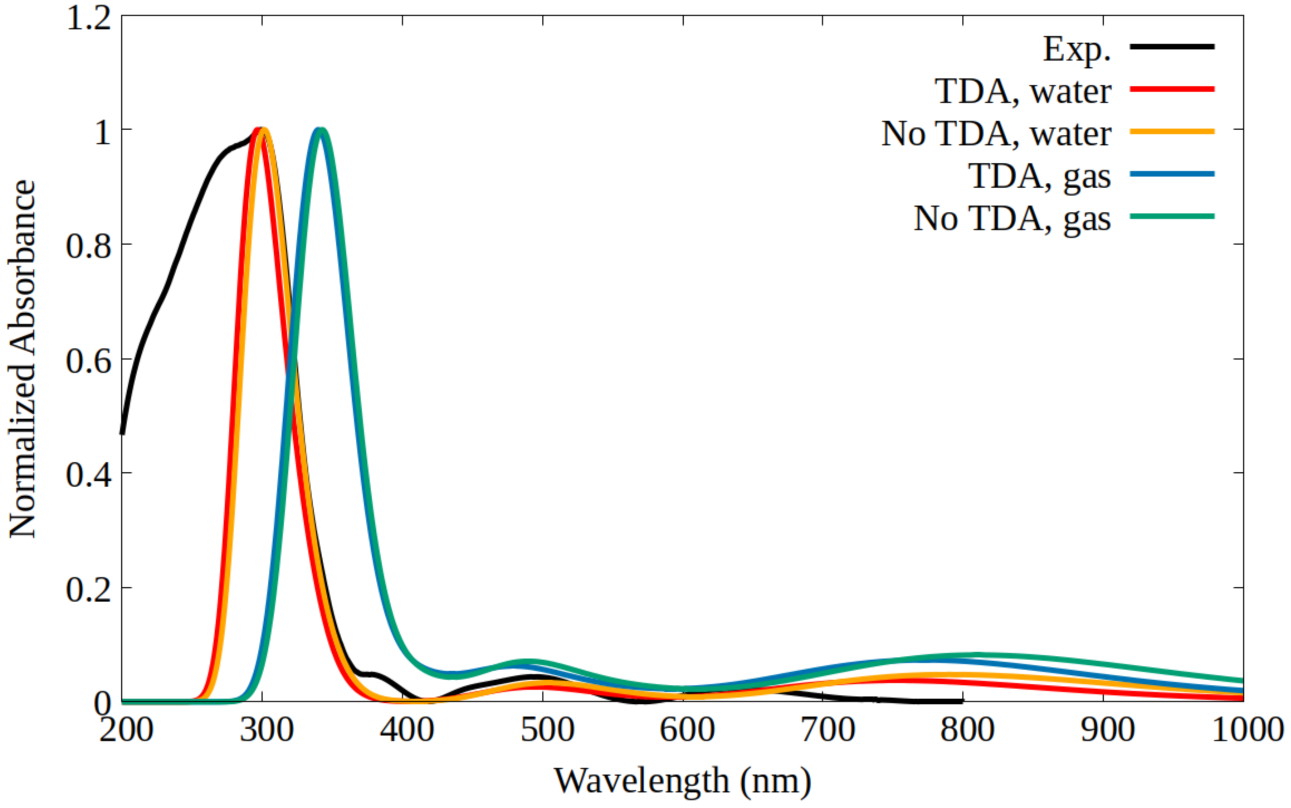

Figure S12. Experimental and computed UV/VIS spectra for **(trans,cis)-NiNi**. The model features an overall spin multiplicity of 5. The computed spectra were obtained with various computational settings for the environment and with and without the TDA approximation. The agreement between theory and experiment improves when an implicit solvation model is used. In addition, the TDA approximation has essentially no effect on the spectra.

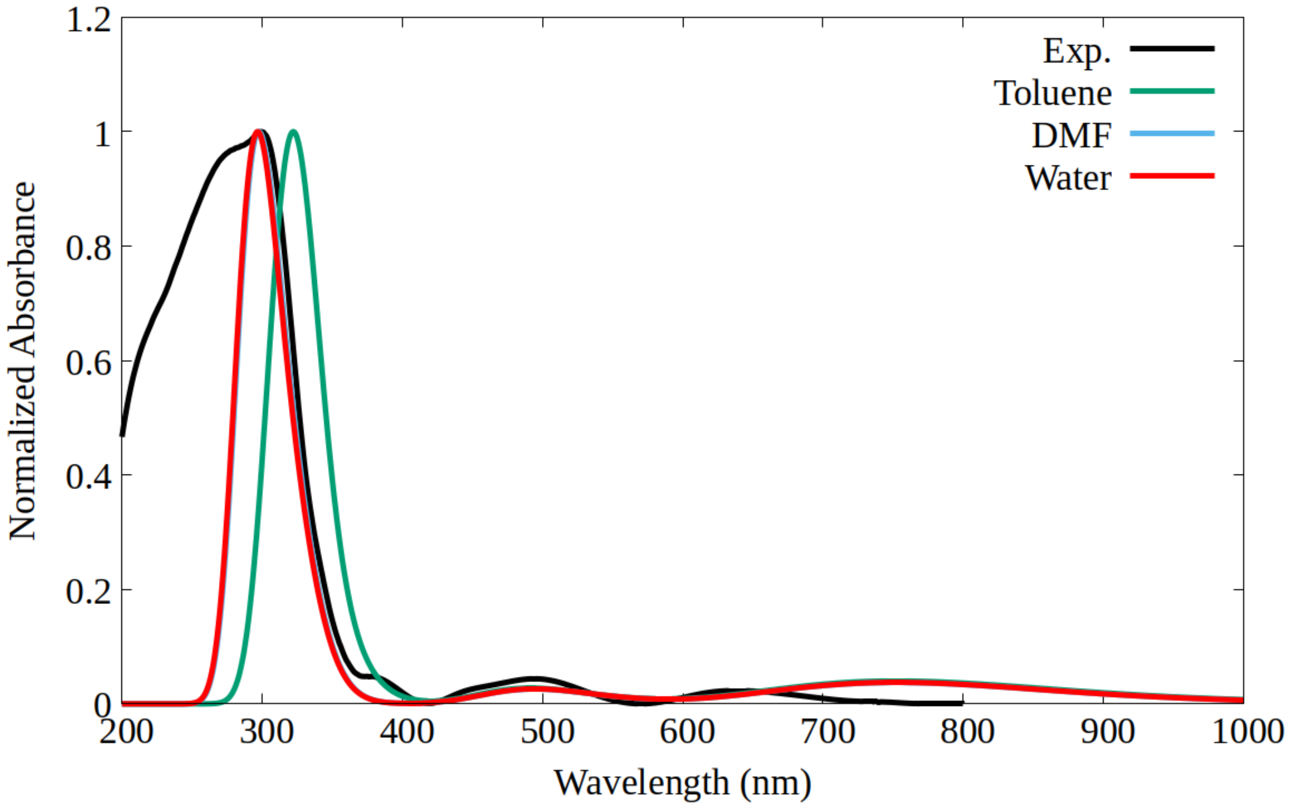

Figure S13. Computed vs experimental UV/VIS spectra for **(trans,cis)-NiNi** considering Toluene, DMF and water within the C-PCM model. While there is not noticeable difference between the spectra computed with water and DMF, which show a remarkable agreement between theory and experiment for both systems, a significant red shift in the maxima is observed with toluene. This is consistent with its significantly smaller dielectric constant.

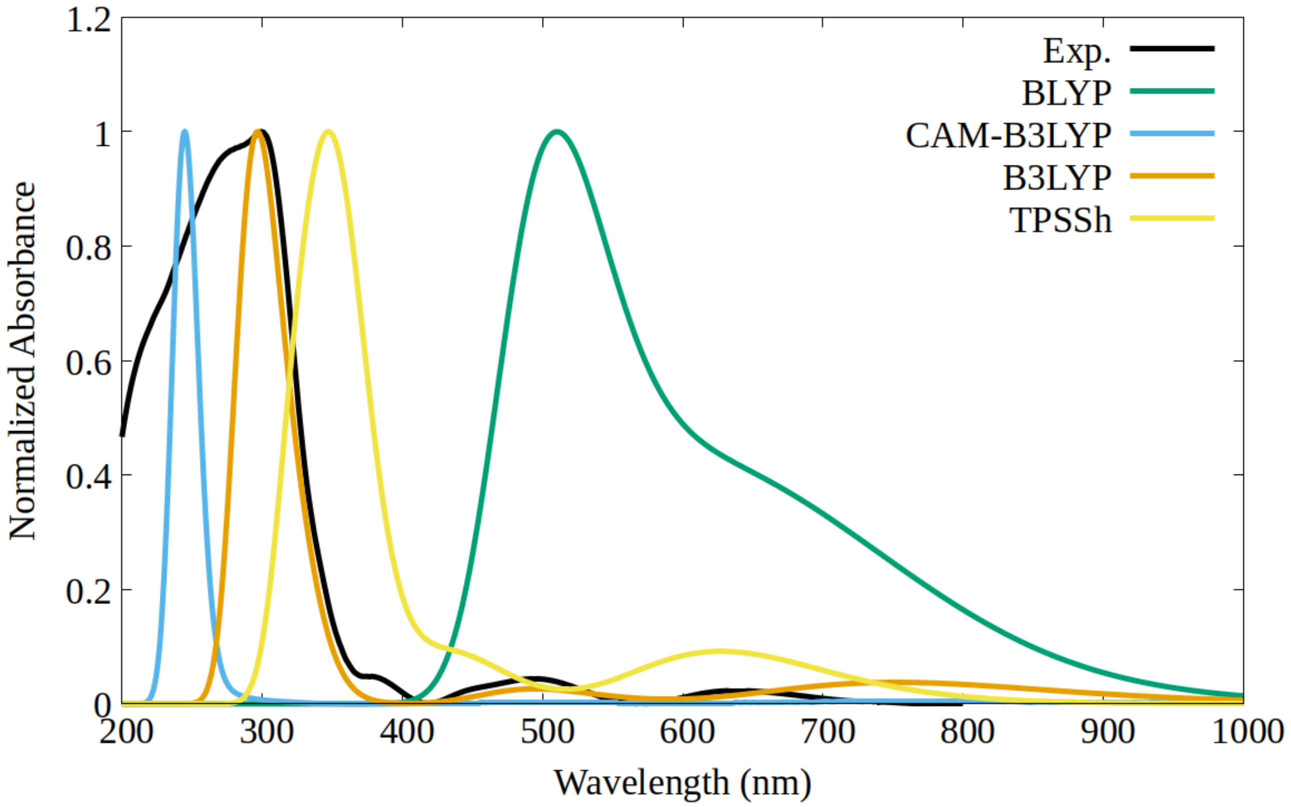

Figure S14. Computed vs experimental UV/VIS spectra for **(trans,cis)-NiNi** using different exchange-correlation functionals (BLYP, B3LYP, CAM-B3LYP and TPSSh). The hybrid B3LYP functional shows the best agreement between theory and experiment.

S11 Ground state electronic structure

Table S4-Table S14 show the energy difference (**ΔE**) between the electronic spin states of the CPs investigated in this work for various conformers and coordination enviroments, together with the corresponding Mulliken spin populations (**SP**) at the metal centers. The results show how each metal prefers a high spin configuration in the lowest energy ground state, regardless of its coordination environment.

Table S4. Electronic energy differences (**ΔE**), Mulliken spin populations (**SP**) and total spin multiplicity (**S_{tot}**) for the various electronic spin states of **(trans,cis)-NiNi**.

S_{tot}	ΔE (eV)	SP Ni(trans)	SP Ni(cis)
**1**	1.79	-	-
**3**	0.64	0.0	1.7
**5**	0	1.7	1.7

Table S5. Electronic energy differences (**ΔE**), Mulliken spin populations (**SP**) and total spin multiplicity (**S_{tot}**) for the various electronic spin states of **(cis)-CoCo**. Both metal centers show the **cis** coordination environment.

S_{tot}	ΔE (eV)	SP Co(cis)	SP Co(cis)
**3**	0.84	0.9	1.0
**5**	0.42	2.8	1.0
**7**	0	2.8	2.8

Table S6. Electronic energy differences (**ΔE**), Mulliken spin populations (**SP**) and total spin multiplicity (**S_{tot}**) for the various electronic spin states of **(trans,cis)-NiCo**. Co and Ni are in **cis** and **trans** coordination environments respectively.

S_{tot}	ΔE (eV)	SP Ni(trans)	SP Co(cis)
**4**	0.41	1.7	0.9
**6**	0	1.7	2.8

Table S7. Electronic energy differences (**ΔE**), Mulliken spin populations (**SP**) and total spin multiplicity (**S_{tot}**) for the various electronic spin states of **(trans,cis)-NiCo**. Ni and Co are in **cis** and **trans** coordination environments respectively.

S_{tot}	ΔE (eV)	SP Co(trans)	SP Ni(cis)
**4**	0.44	1.0	1.7
**6**	0	2.8	1.7

Table S8. Electronic energy differences (**ΔE**), Mulliken spin populations (**SP**) and total spin multiplicity (**S_{tot}**) for the various electronic spin states of **(trans,cis)-NiFe**. Fe and Ni are in **cis** and **trans** coordination environments respectively.

S_{tot}	ΔE (eV)	SP Ni(trans)	SP Fe(cis)
**3**	1.36	0.0	2.0

**5**	0.75	1.7	2.0
**7**	0	1.7	3.8

Table S9. Electronic energy differences (**ΔE**), Mulliken spin populations (**SP**) and total spin multiplicity (**S_{tot}**) for the various electronic spin states of **(trans,cis)-NiFe**. Ni and Fe are in **cis** and **trans** coordination environments respectively.

S_{tot}	ΔE (eV)	SP Ni(trans)	SP Fe(cis)
**3**	0.68	1.7	0.0
**5**	0.67	1.7	2.0
**7**	0	1.7	3.8

Table S10. Electronic energy differences (**ΔE**), Mulliken spin populations (**SP**) and total spin multiplicity (**S_{tot}**) for the various electronic spin states of **(trans,cis)-NiMn**. Mn and Ni are in **cis** and **trans** coordination environments respectively.

S_{tot}	ΔE (eV)	SP Ni(trans)	SP Mn(cis)
**6**	0.64	0.0	4.9
**8**	0	1.7	4.9

Table S11. Electronic energy differences (**ΔE**), Mulliken spin populations (**SP**) and total spin multiplicity (**S_{tot}**) for the various electronic spin states of **(trans,cis)-NiMn**. Ni and Mn are in **cis** and **trans** coordination environments respectively.

S_{tot}	ΔE (eV)	SP Mn(trans)	SP Ni(cis)

**6**	0.61	4.9	0.0
**8**	0	4.9	1.7

Table S12. Electronic energy differences (**ΔE**), Mulliken spin populations (**SP**) and total spin multiplicity (**S_{tot}**) for the various electronic spin states of **(cis)-NiCo**. Both metal centers show the **cis** coordination environment.

S_{tot}	ΔE (eV)	SP Ni(cis)	SP Co(cis)
**4**	0.40	1.7	1.0
**6**	0	1.7	2.8

Table S13. Electronic energy differences (**ΔE**), Mulliken spin populations (**SP**) and total spin multiplicity (**S_{tot}**) for the various electronic spin states of **(cis)-CoFe**. Both metal centers show the **cis** coordination environment.

S_{tot}	ΔE (eV)	SP Co(cis)	SP Fe(cis)
**2**	1.08	1.0	0.0
**4**	0.69	2.8	0.0
**6**	0.41	1.0	3.8
**8**	0	2.8	3.8

Table S14. Electronic energy differences (**ΔE**), Mulliken spin populations (**SP**) and total spin multiplicity (**S_{tot}**) for the various electronic spin states of **(cis)-MnFe**. Both metal centers show the **cis** coordination environment.

S_{tot}	ΔE (eV)	SP Mn(cis)	SP Fe(cis)
**2**	2.32	1.1	0.0
**4**	2.38	1.1	2.0
**6**	0.80	4.9	0.0
**8**	0.75	4.9	2.0
**10**	0	4.9	3.8

S12 Validation of the ground state spin configuration

In **Figure S15Figure S18** the experimental (black) and the computed absorption spectra for **(trans,cis)-NiNi**, **(trans,cis)-NiCo**, **(trans,cis)-NiFe** and **(cis)-MnFe** with different total spin mutliplicities (**S_{tot}**) are shown. The best agreement between experimental end computed spectra is obtained when considering each metal center in its highest spin configuration. This is consistent with the thermodynamic evaluation of the energy difference between the electronic spin states, demonstrating that the ground state features both metal centers in their high-spin configuration. The computed spectra for the bimetallic CPs are the convolution of the spectra obtained for both coordination environment combinations, i.e., the M1(trans)M2(cis) and M1(trans)M2(cis) clusters. This was done in order to consider the dependency of the optical properties on the coordination environment for the two metal centers in the bimetallic case.

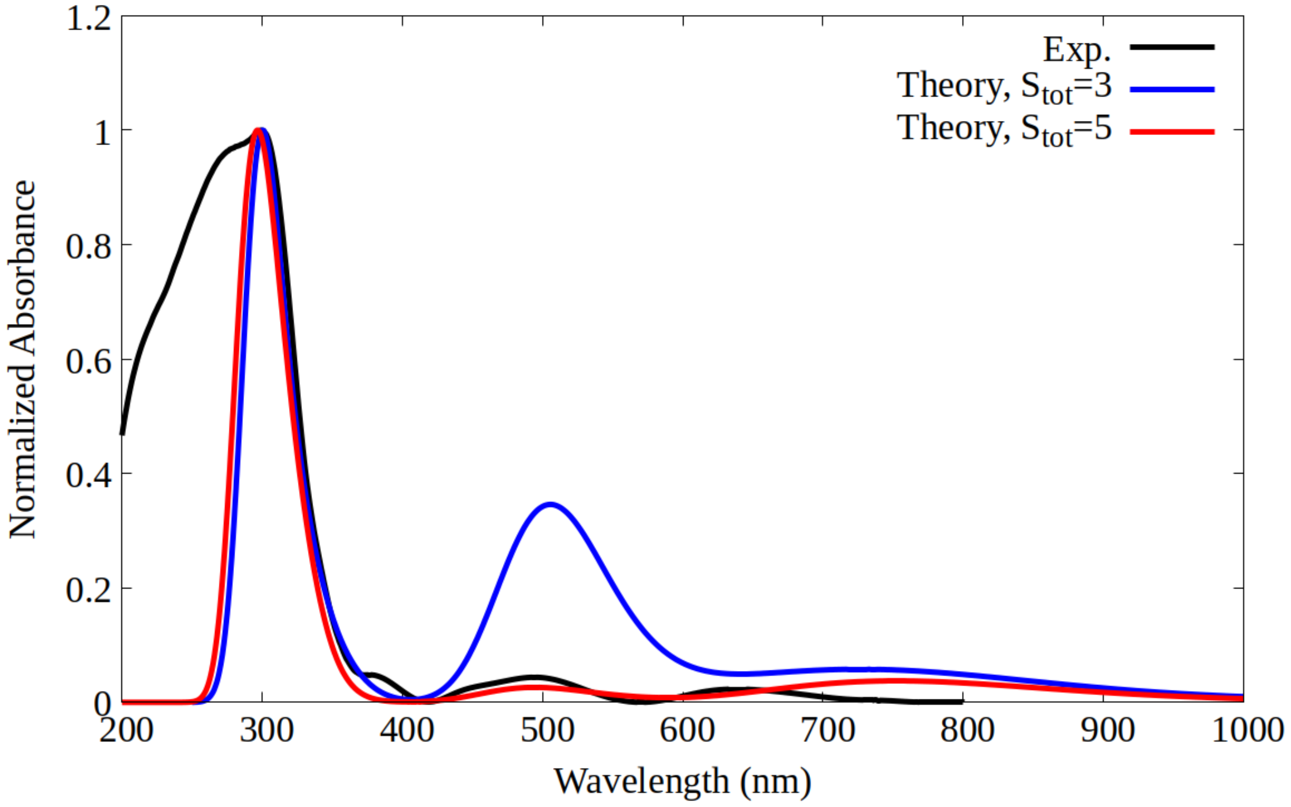

Figure S15. Comparison of the experimental and theoretical spectra for **(trans,cis)-NiNi** with total **S_{tot}** equal to 3 and 5.

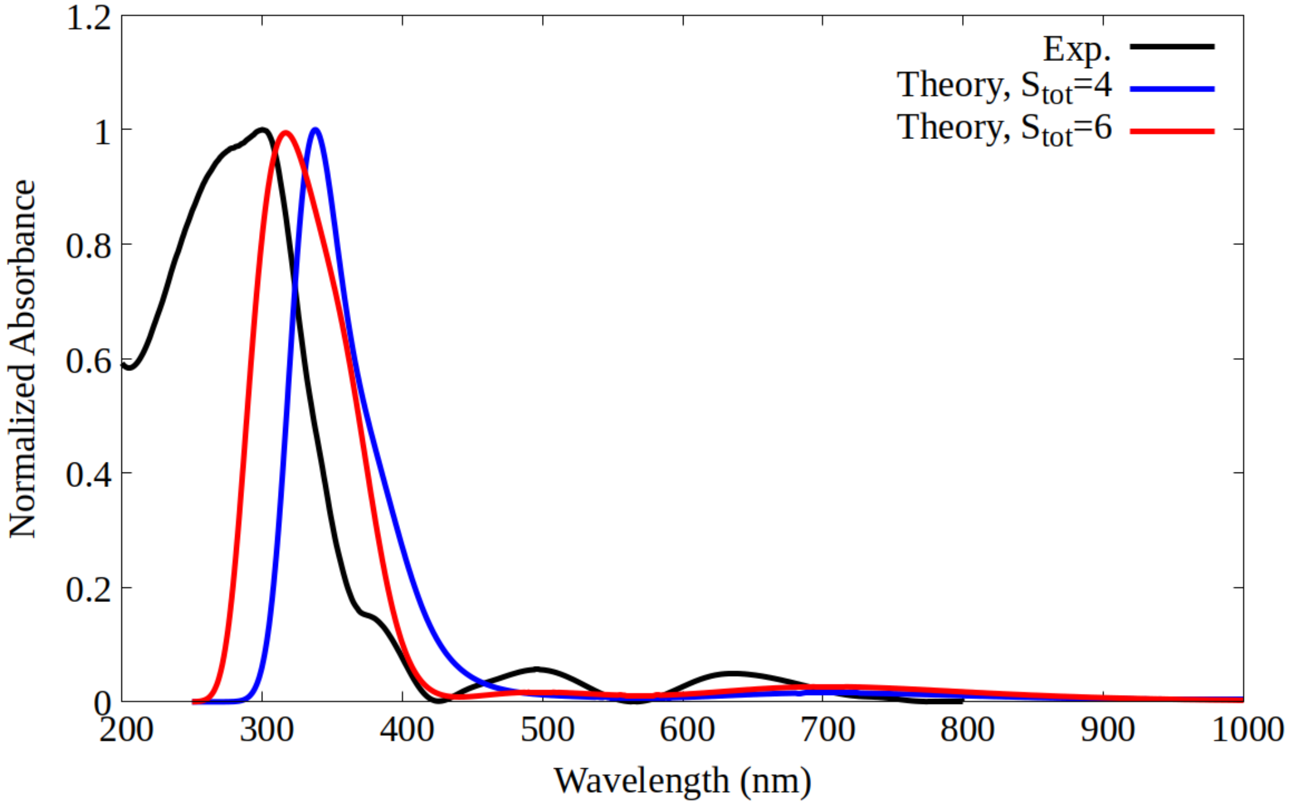

Figure S16. Comparison of the experimental and theoretical spectra for **(trans,cis)-NiCo** with S_{tot} equal to 4 and 6.

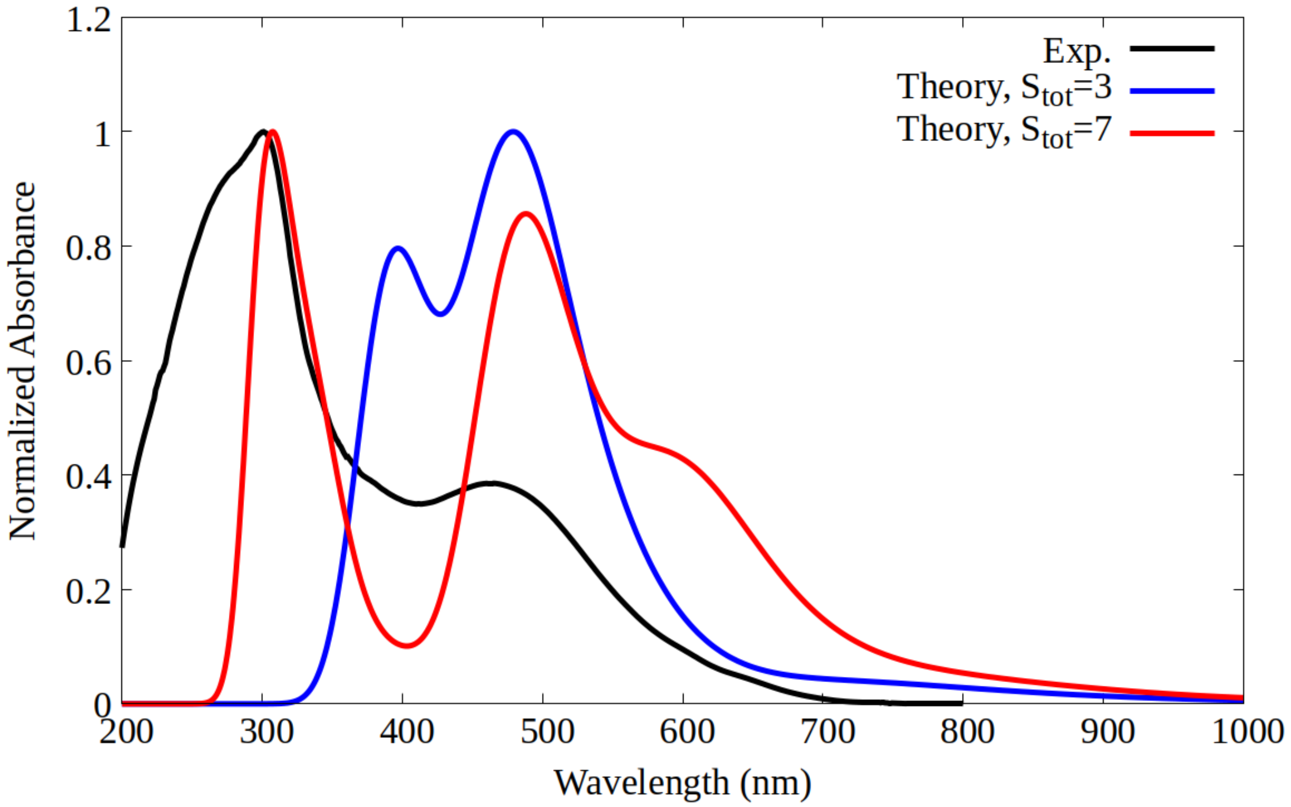

Figure S17. Comparison of the experimental and theoretical spectra for **(trans,cis)-NiFe** with total S_{tot} equal to 3 and 7.

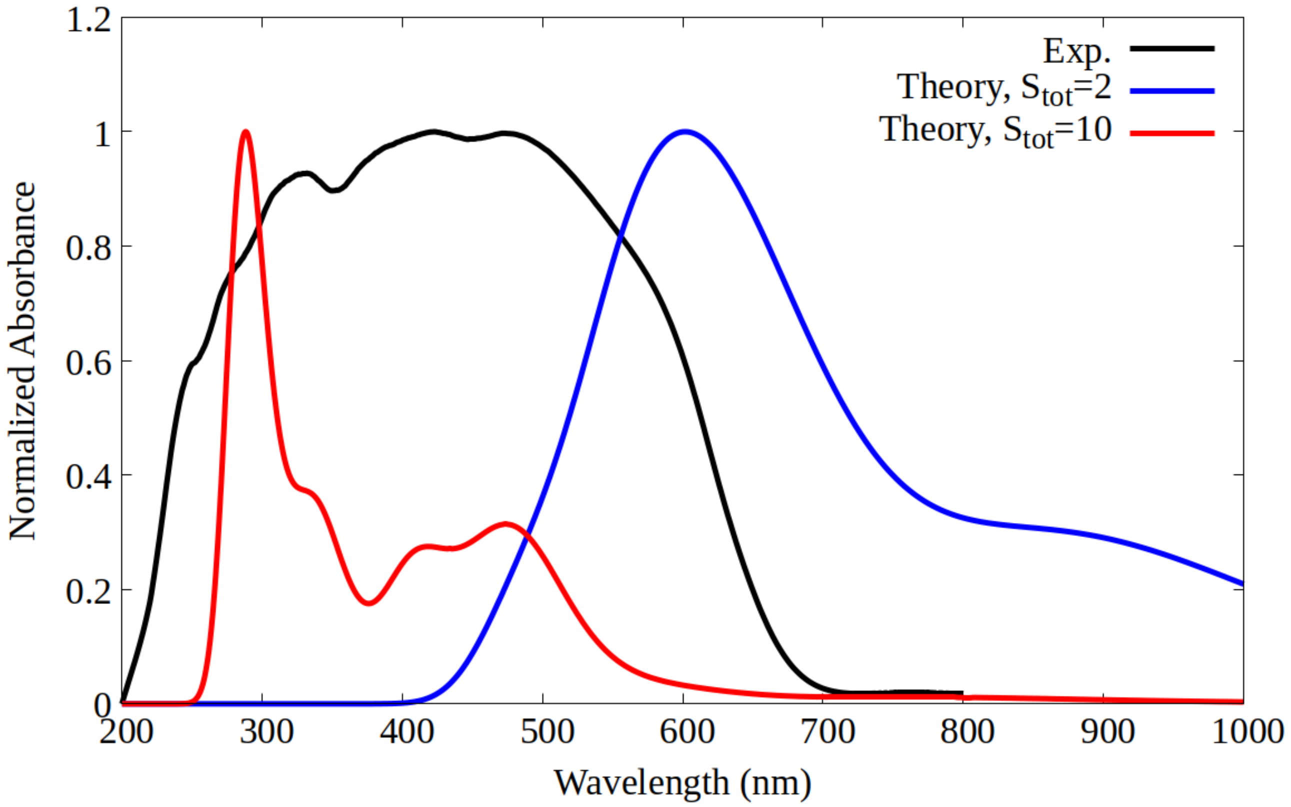

Figure S18. Comparison of the experimental and theoretical spectra for **(cis)-MnFe** with total **S$_{tot}$** equal to 2 and 10.

S13 Electronic structure characterization

To provide a visual representation of the ground state electronic structure of the systems considered in this work, we provide a qualitative orbital energy diagram for prototypical examples using quasi-restricted orbitals (QROs).

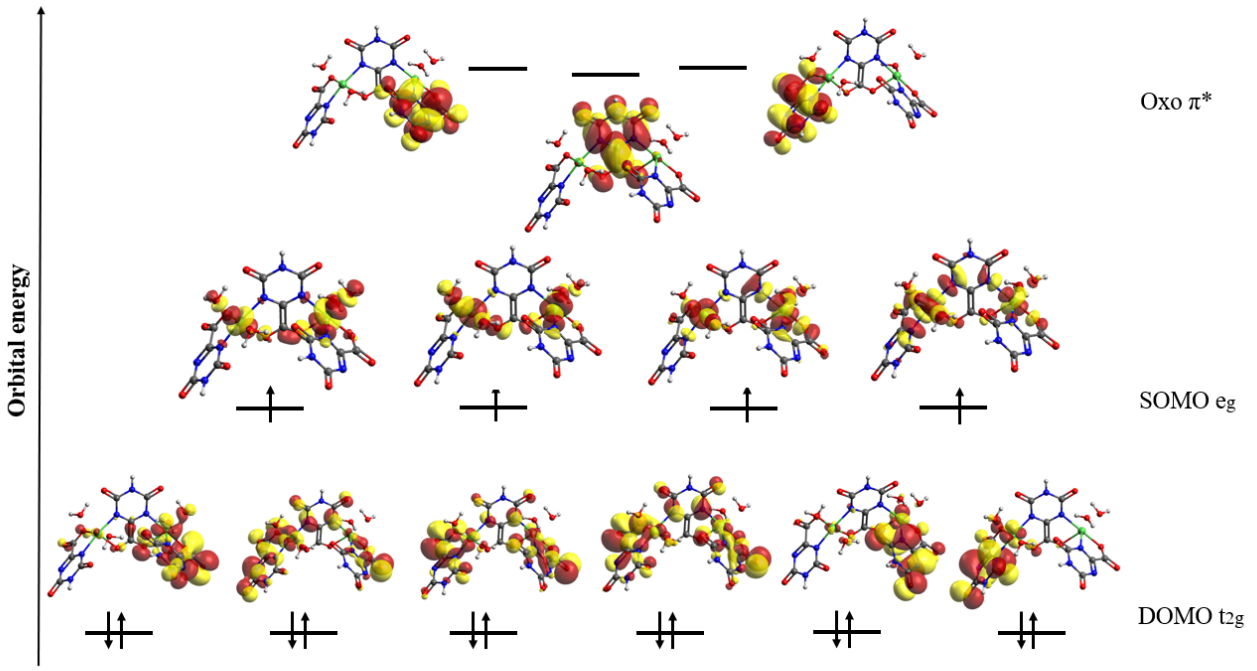

Figure S19. Electronic structure of **(trans,cis)-NiNi** for $\mathbf{S_{tot}}$ equal to 5.

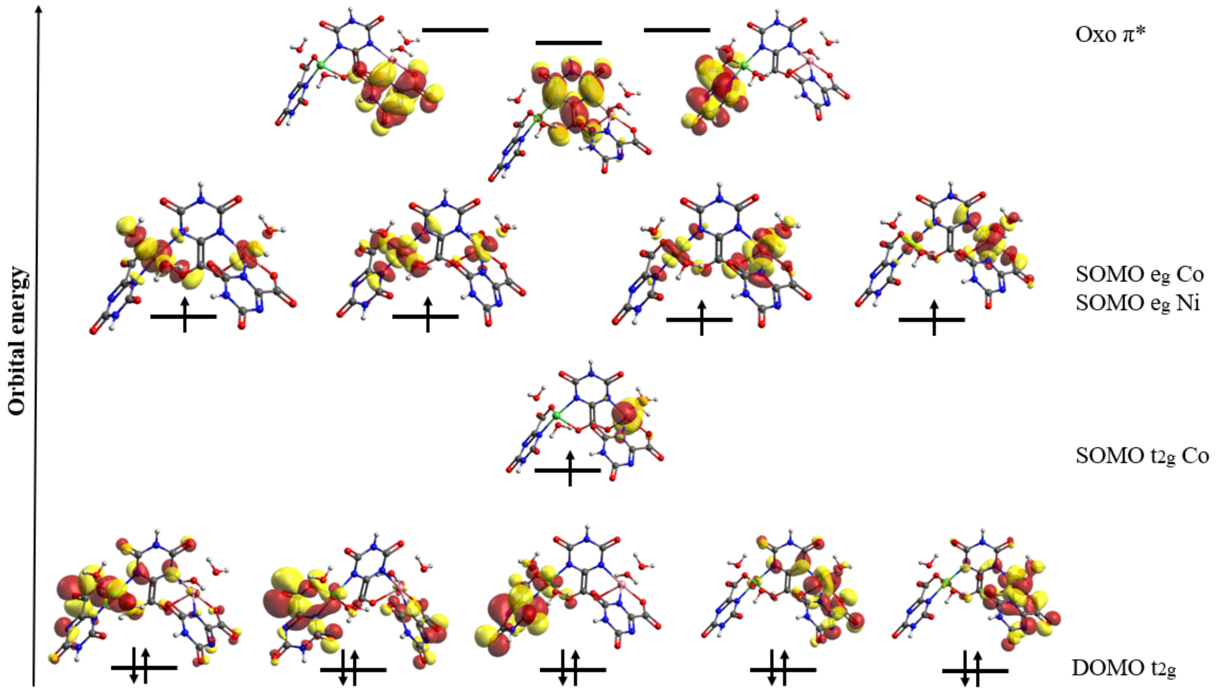

Figure S20. Electronic structure of **(trans,cis)-NiCo** for $\mathbf{S_{tot}}$ equal to 6, with Co (pink) and Ni (green) in

cis and **trans** coordination environments respectively.

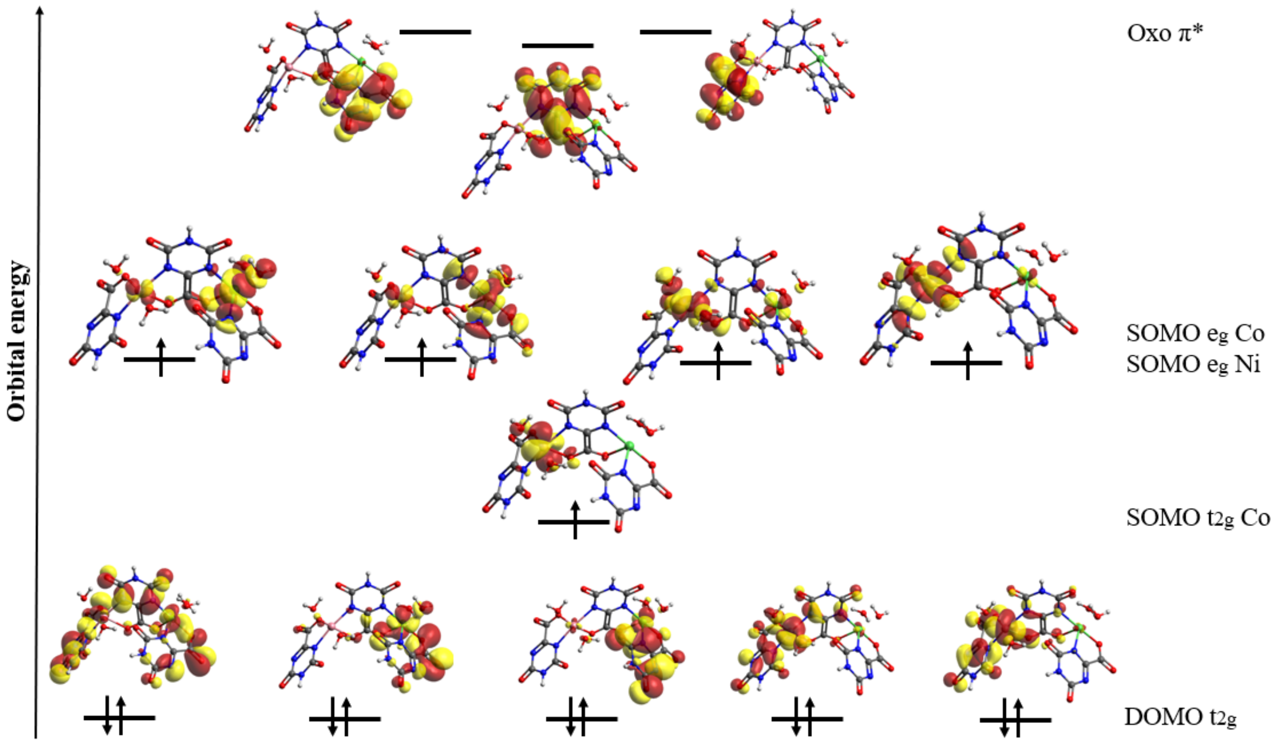

Figure S21. Electronic structure of **(trans,cis)-NiCo** for $\mathbf{S_{tot}}$ equal to 6, with Ni (green) and Co (pink) in **cis** and **trans** coordination environments respectively.

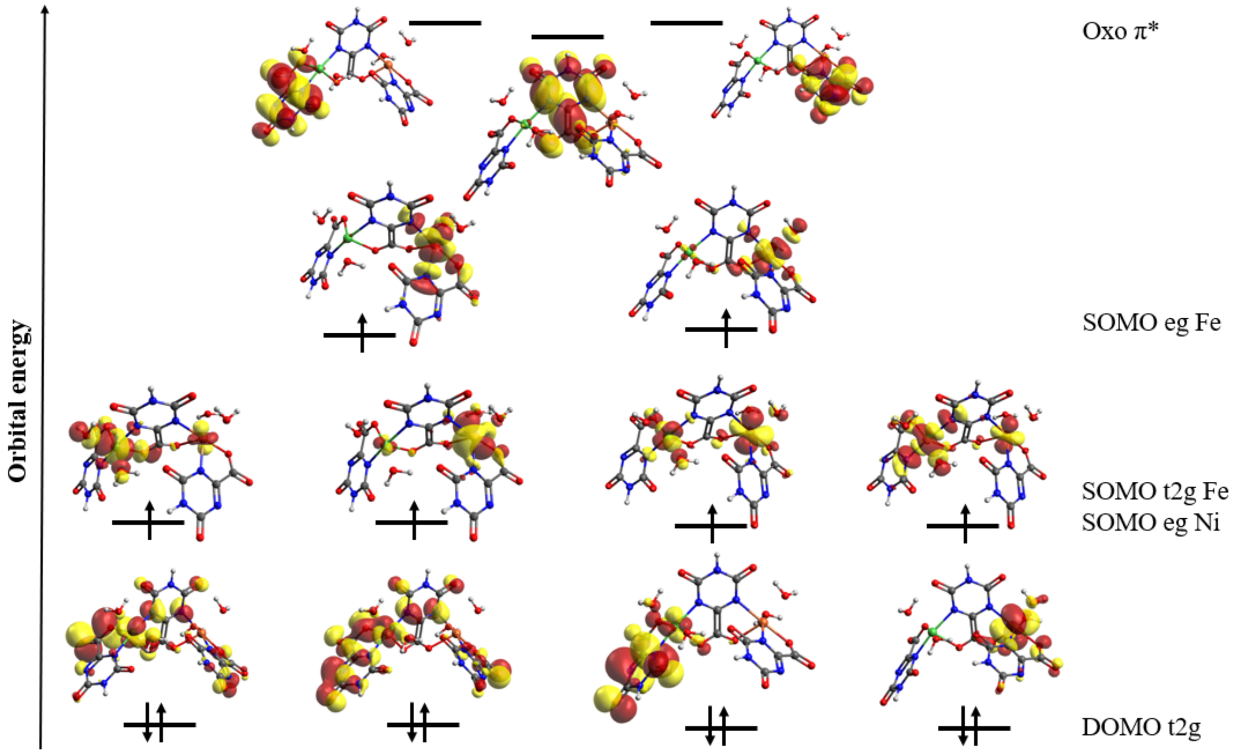

Figure S22. Electronic structure of **(trans,cis)-NiFe** for $\mathbf{S_{tot}}$ equal to 7, with Fe (orange) and Ni (green)

in **cis** and **trans** coordination environments respectively.

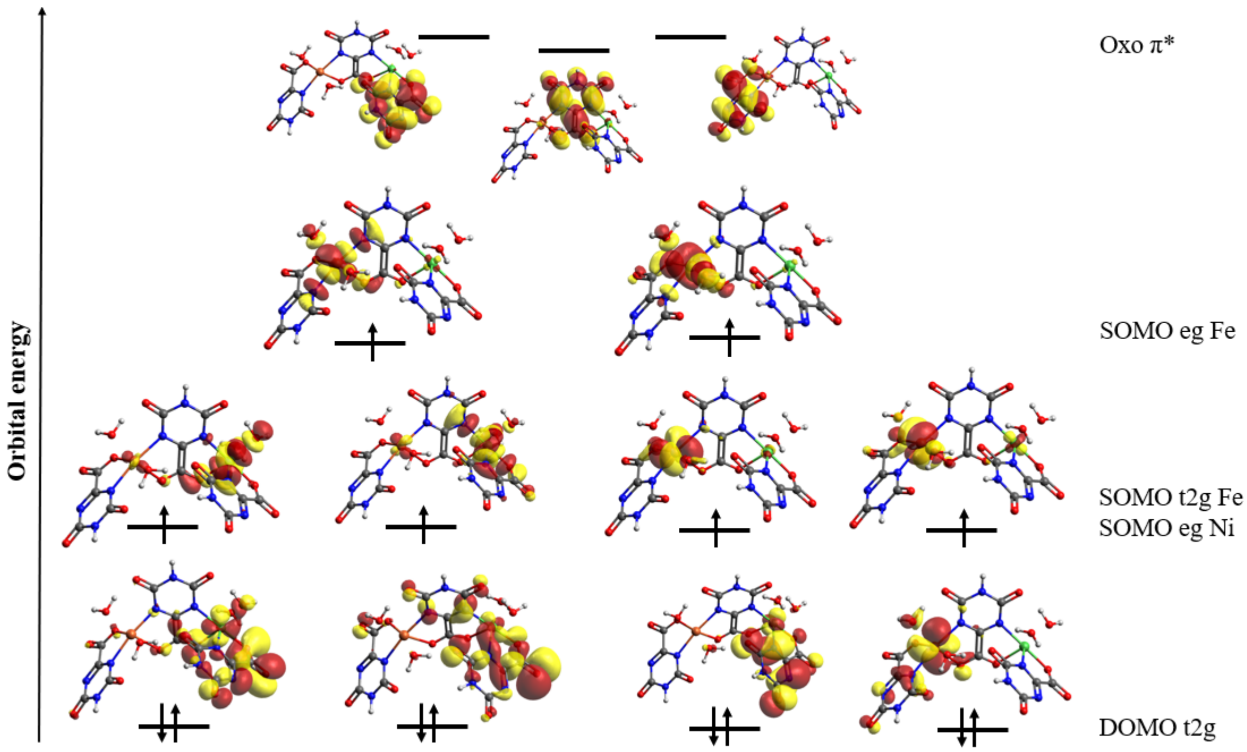

Figure S23. Electronic structure of **(trans,cis)-NiFe** for $\mathbf{S_{tot}}$ equal to 6, with Ni (green) and Fe (orange) in **cis** and **trans** coordination environments respectively.

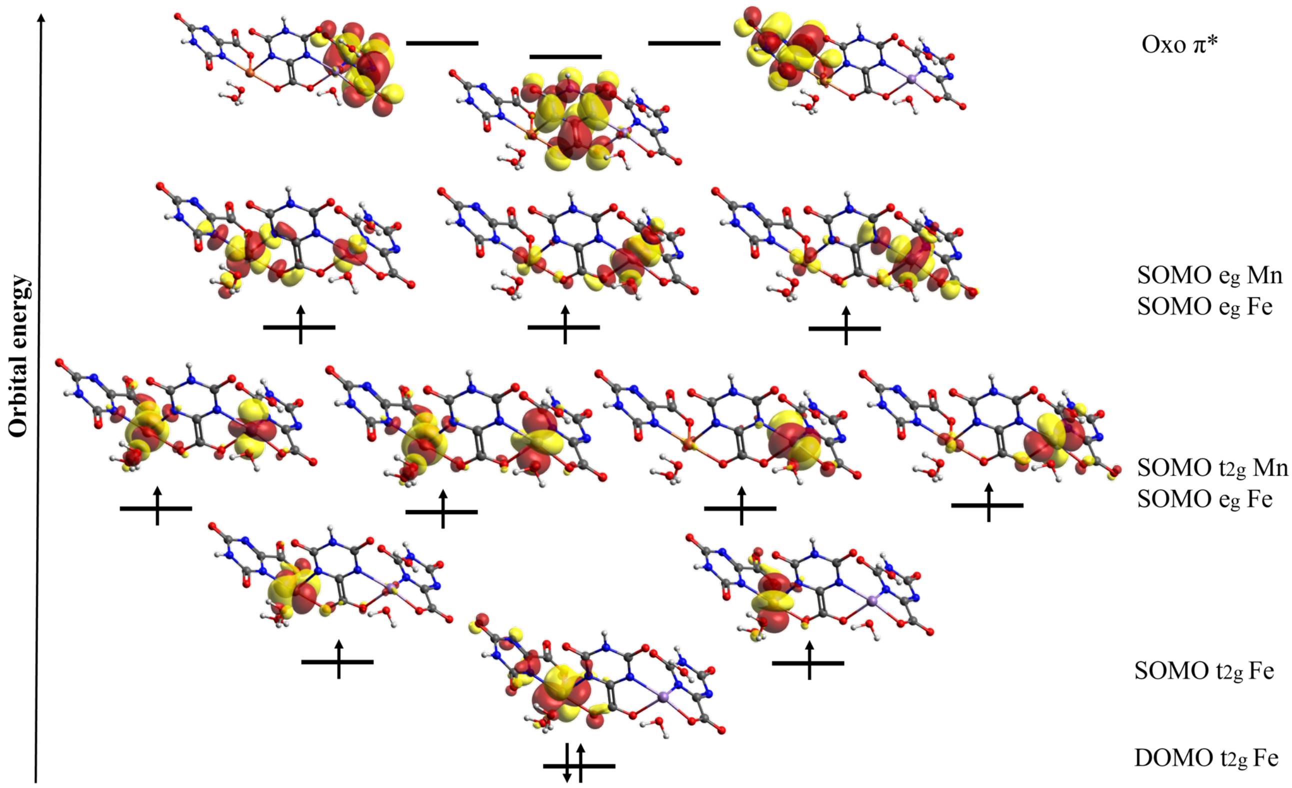

Figure S24. Electronic structure of **(cis)-MnFe** for $\mathbf{S_{tot}}$ equal to 10, with both Fe (orange) and Mn (purple)

in **cis** coordination environment.

S14 Electronic transitions characterization

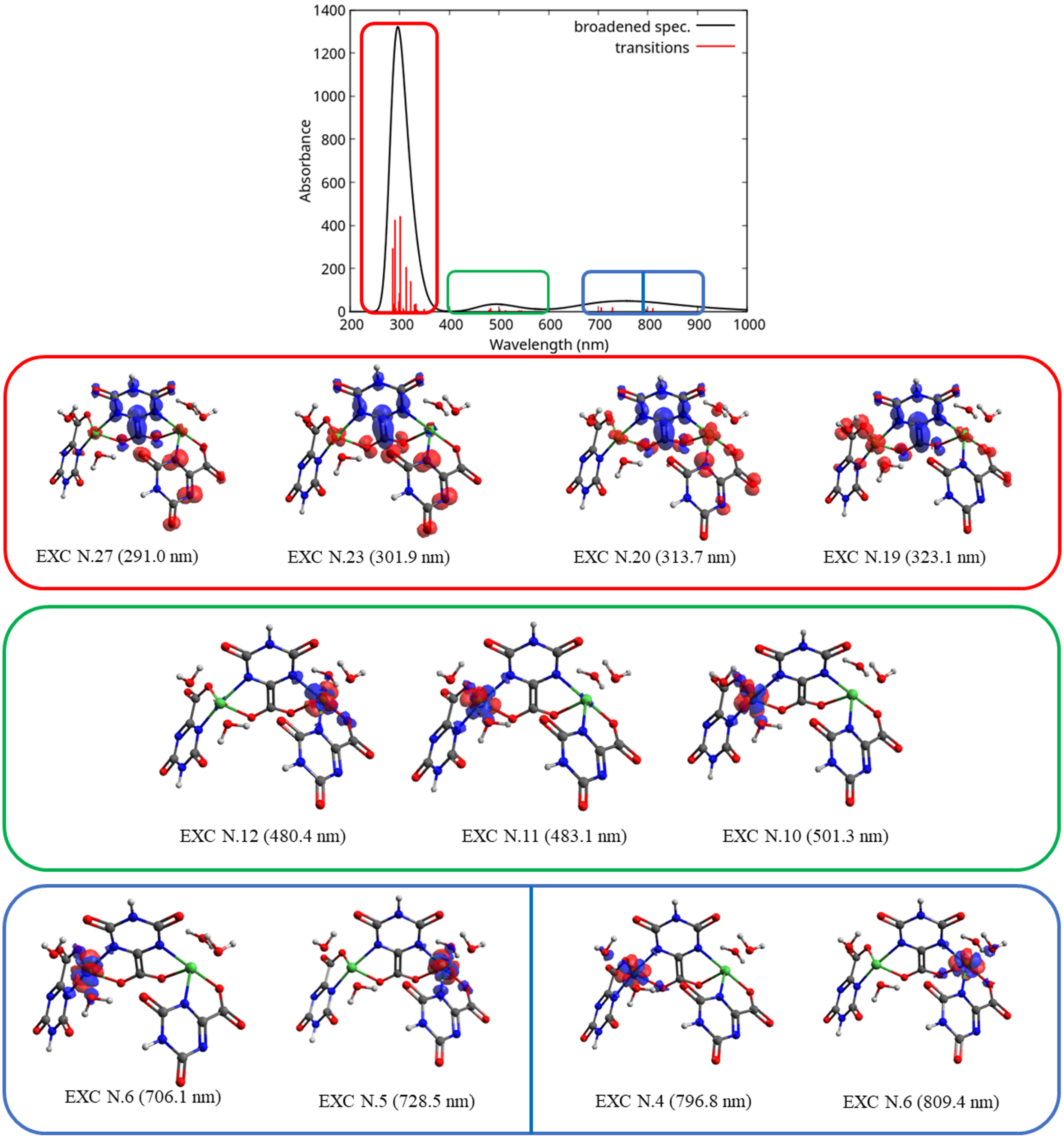

Figure S25. Characterization of the electronic excitations using difference densities for **(trans,cis)-NiNi** with $\mathbf{S_{tot}}$ equal to 5. Red surfaces correspond to regions of charge depletion, while blue surfaces correspond to regions of charge accumulation.

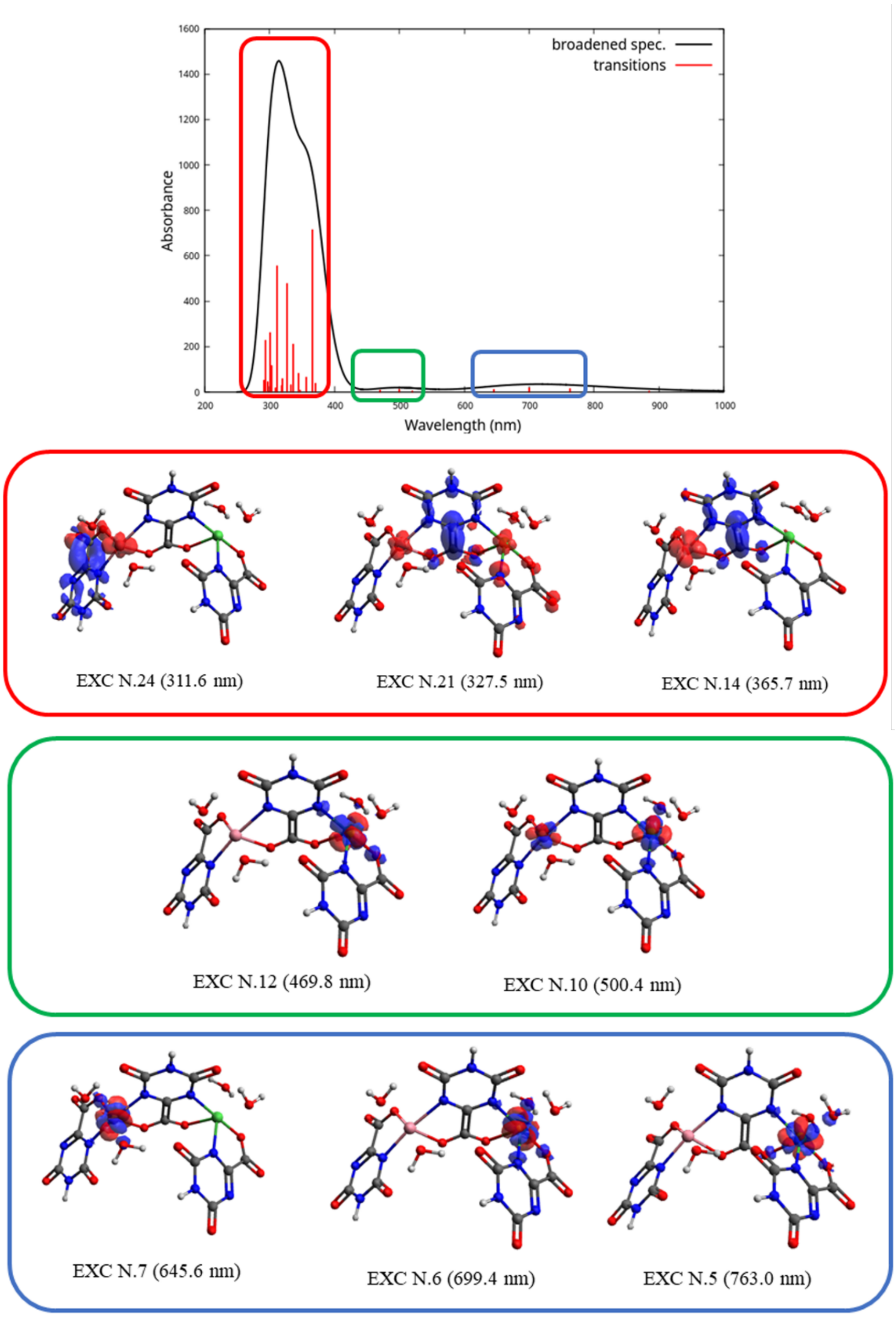

Figure S26. Characterization of the electronic excitations using difference densities for **(trans,cis)-NiCo**, where Ni (green) and Co (pink) are in **cis** and **trans** coordination environments respectively, and $\mathbf{S_{tot}}$ equal to 6. Red surfaces correspond to regions of charge depletion, while blue surfaces correspond to regions of charge accumulation.

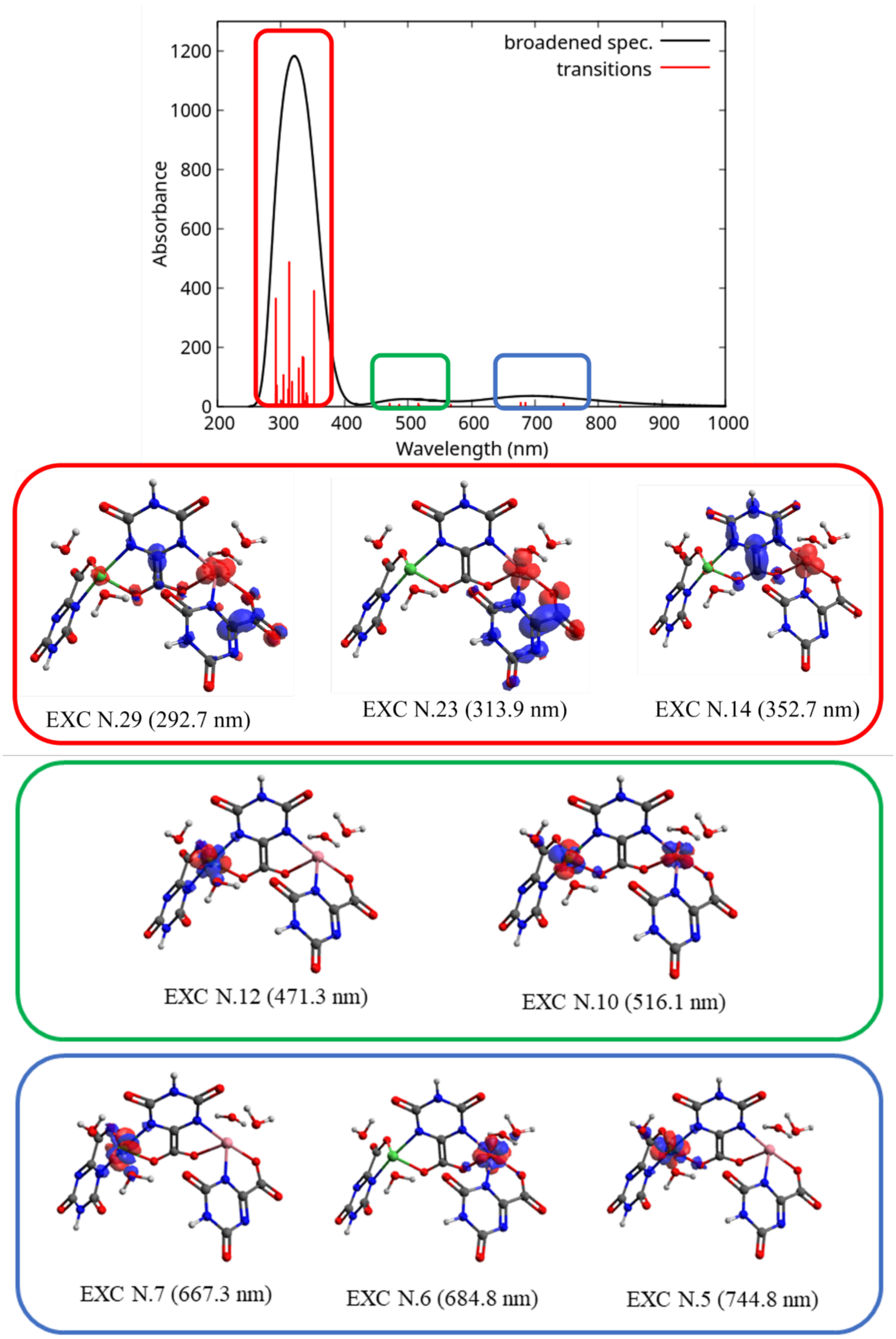

Figure S27. Characterization of the electronic excitations using difference densities for **(trans,cis)-NiCo**, where Ni (green) and Co (pink) are in **trans** and **cis** coordination environments respectively, and $\mathbf{S_{tot}}$ equal to 6. Red surfaces correspond to regions of charge depletion, while blue surfaces correspond to regions of charge accumulation.

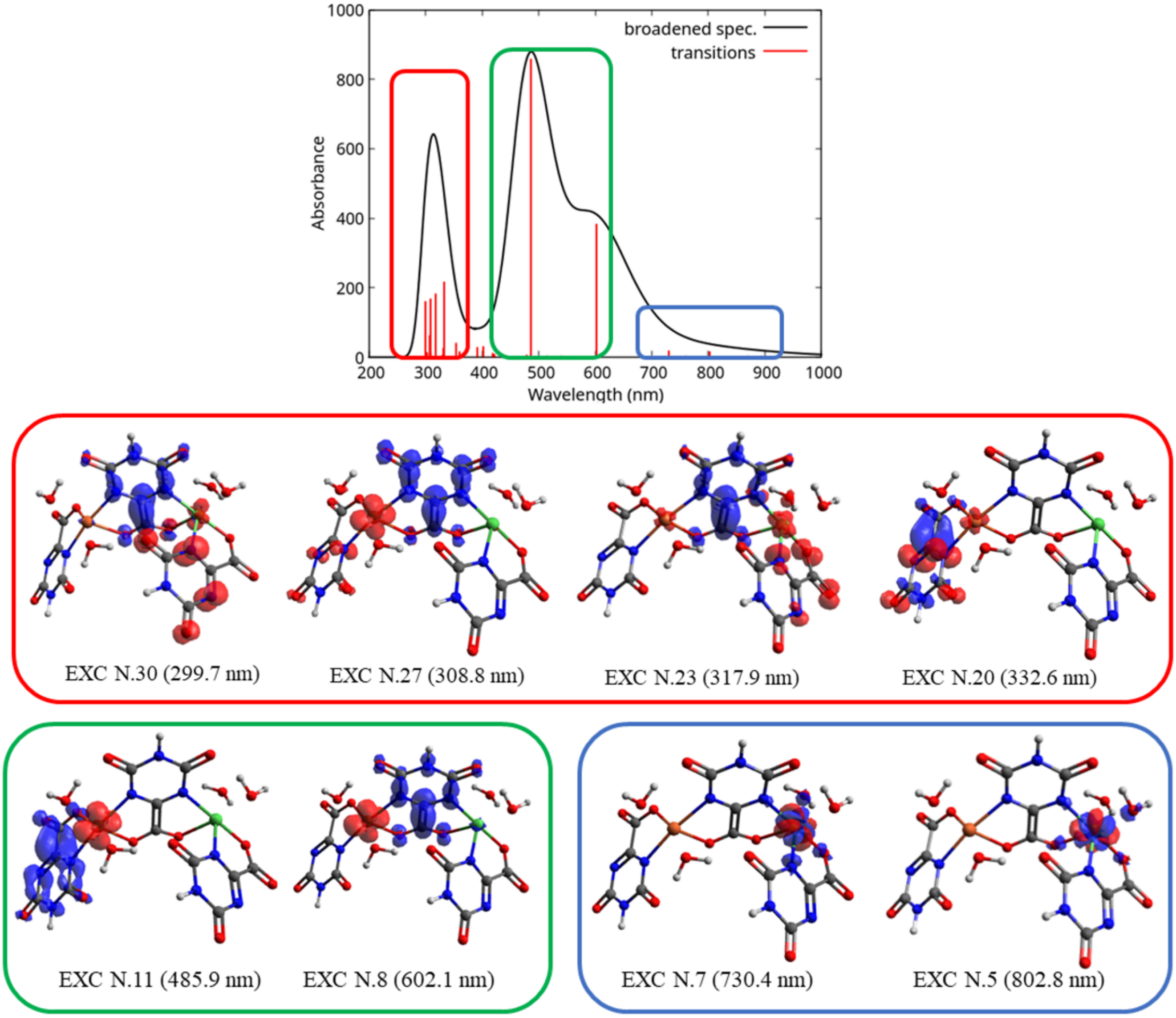

Figure S28. Characterization of the electronic excitations using difference densities for **(trans,cis)-NiFe**, where Ni (green) and Fe (orange) are in **cis** and **trans** coordination environments respectively, and $\mathbf{S_{tot}}$ equal to 7. Red surfaces correspond to regions of charge depletion, while blue surfaces correspond to regions of charge accumulation.

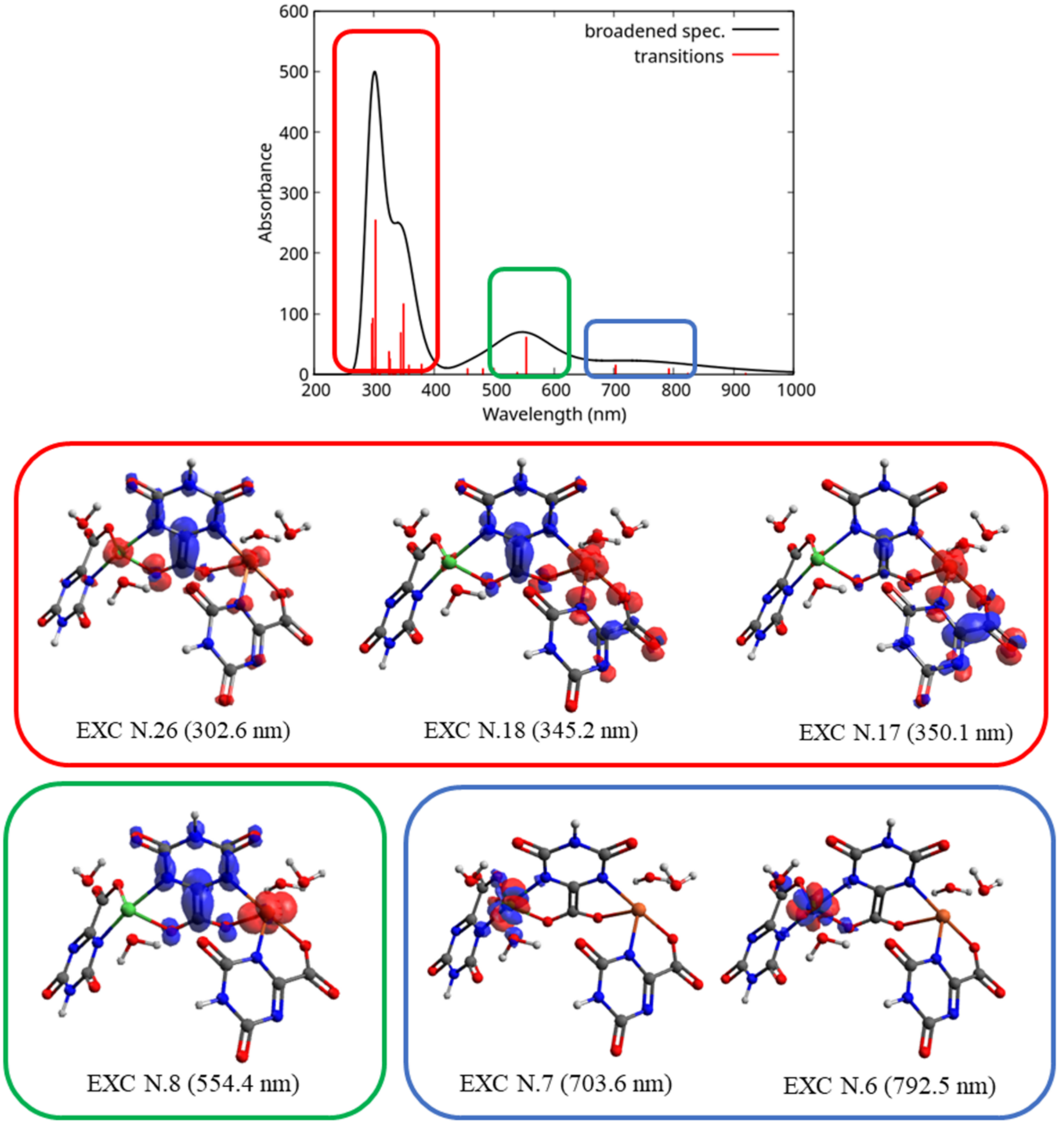

Figure S29. Characterization of the electronic excitations using difference densities for **(trans,cis)-NiFe**, where Ni (green) and Fe (orange) are in **trans** and **cis** coordination environments respectively, and $\mathbf{S_{tot}}$ equal to 7. Red surfaces correspond to regions of charge depletion, while blue surfaces correspond to regions of charge accumulation.

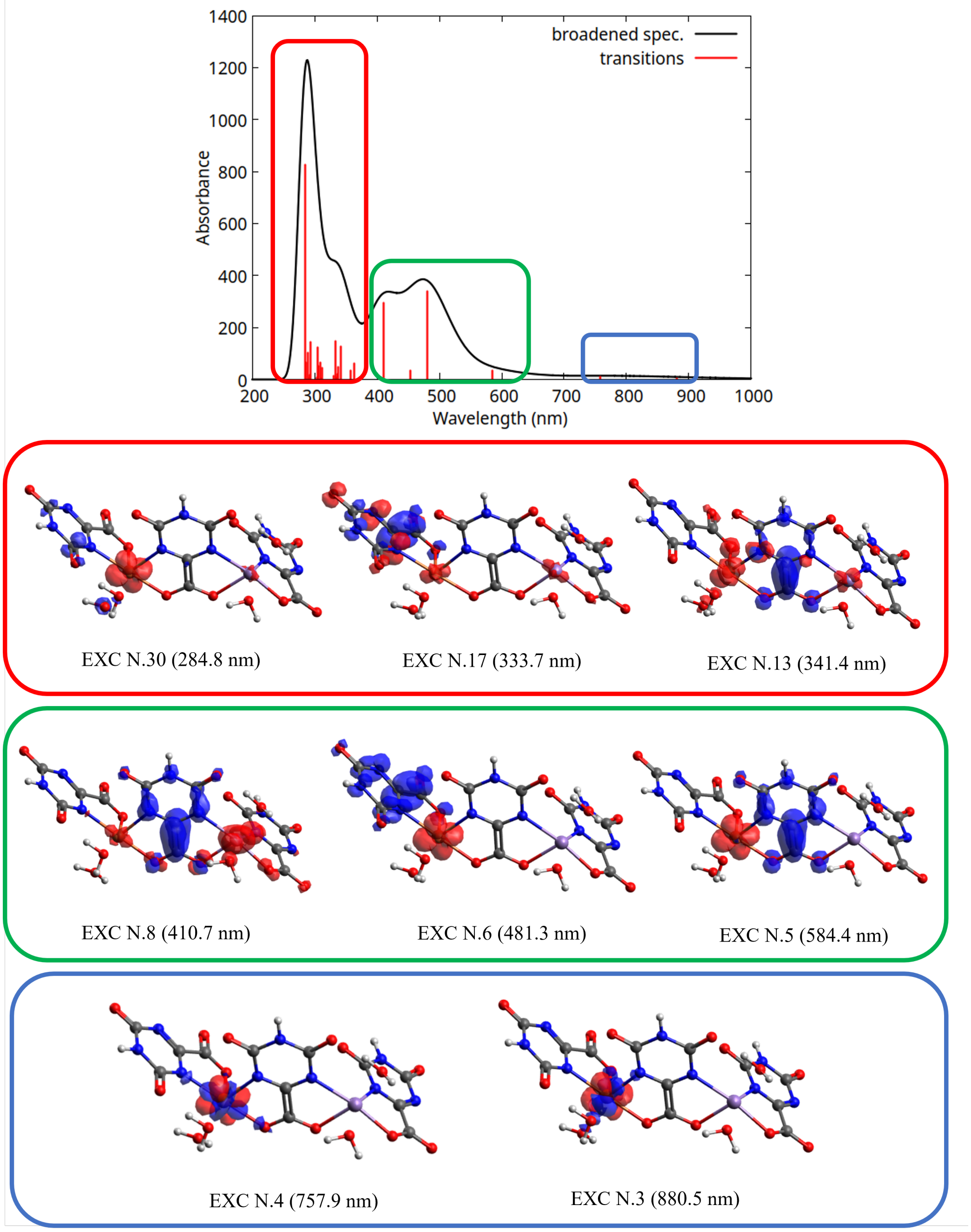

Figure S30. Characterization of the electronic excitations using difference densities for **(cis)-MnFe**, where both Mn (violet) and Fe (orange) are in **cis** coordination environments, and $\mathbf{S_{tot}}$ equal to 10. Red surfaces correspond to regions of charge depletion, while blue surfaces correspond to regions of charge accumulation.

S15 OER reaction free energies in alkaline conditions

To obtain the free energy diagrams for the catalysts considered in this work, it is necessary to derive the reaction energies for each elementary step under alkaline conditions:[17–19]

$$M + OH^- \rightarrow MOH + e^- \quad \text{Eq. S1}$$

$$MOH + OH^- \rightarrow MO + H_2O + +e^- \quad \text{Eq. S2}$$

$$MO + OH^- \rightarrow MOOH + e^- \quad \text{Eq. S3}$$

$$MOOH + OH^- \rightarrow M + O_2 + H_2O + e^- \quad \text{Eq. S4}$$

In which M is the catalyst (in the present example, the catalyst features an uncoordinated site that can be occupied by the incoming OH). ΔG_i is defined as the difference of the electrochemical potentials of products and reagents for all the four steps:

$$\Delta G_1 = \mu_{OH*} - \mu_{i*} - (\mu_{OH^-} - \mu_{e^-}) \quad \text{Eq. S5}$$

$$\Delta G_2 = \mu_{O*} + \mu_{H_2O} - \mu_{OH*} - (\mu_{OH^-} - \mu_{e^-}) \quad \text{Eq. S6}$$

$$\Delta G_3 = \mu_{OOH*} - \mu_{O*} - (\mu_{OH^-} - \mu_{e^-}) \quad \text{Eq. S7}$$

$$\Delta G_4 = \mu_{i*} + \mu_{O_2} + \mu_{H_2O} - \mu_{OOH*} - (\mu_{OH^-} - \mu_{e^-}) \quad \text{Eq. S8}$$

Where μ are the electrochemical potentials of the species involved in the steps (* denotes the intermediate with a specific species absorbed on the metal).

The O_2 potential is derived from the experimental standard free energy formation of the reaction $\frac{1}{2}O_{2(g)} + H_{2(g)} \leftrightarrow H_2O_{(l)}$, which amounts to 2.46eV. It follows that the O_2 potential is equal to:

$$\mu_{O_2(g)} = 4.92 + 2\mu_{H_2O(l)} - 2\mu_{H_2(g)} \quad \text{Eq. S9}$$

To obtain OH^- and e^- potentials (only their difference is needed, see **Eq. S5 Eq. S8**), we assume the equilibrium:

$$H_2O \leftrightarrow H^+ + OH^- \quad \text{Eq. S10}$$

The related potentials will be:

$$\mu_{H_2O} = \mu_{H^+} + \mu_{OH^-} \quad \text{Eq. S11}$$

Adding to both sides the same term μ_{e^-} we obtain:

$$\mu_{OH^-} - \mu_{e^-} = \mu_{H_2O} - (\mu_{H^+} + \mu_{e^-}) \quad \text{Eq. S12}$$

The $(\mu_{H^+} + \mu_{e^-})$ term can be calculated using the computational hydrogen electrode (CHE) approach,[20] assuming the equilibrium:

$$H^+ + e^- \leftrightarrow \frac{1}{2}H_2 \quad \text{Eq. S13}$$

The final OER energy profiles are expressed using the reversible hydrogen electrode (RHE) as reference:

$$\Delta G_1 = \mu_{OH*} - \mu_i - \mu_{H_2O} + \frac{1}{2}\mu_{H_2} - eU_{RHE} \quad \text{Eq. S14}$$

$$\Delta G_2 = \mu_{O*} - \mu_{OH*} + \frac{1}{2}\mu_{H_2} - eU_{RHE} \quad \text{Eq. S15}$$

$$\Delta G_3 = \mu_{OOH*} - \mu_{O*} - \mu_{H_2O} + \frac{1}{2}\mu_{H_2} - eU_{RHE} \quad \text{Eq. S16}$$

$$\Delta G_4 = \mu_{i*} - \mu_{OOH*} + \mu_{O_2} + \frac{1}{2}\mu_{H_2} - eU_{RHE} \quad \text{Eq. S17}$$

In the CPs under study, two water molecules are already coordinated to each metal center, and hence each node presents two active sites. Unless otherwise specified, we will assume that the reaction occurs on a single active site, while keeping all the remaining positions coordinated with water. Notably, the dissociation energy of a single water molecule under the experimental conditions is close to 0 eV.

S16 Reaction intermediates analysis

Table S15. Intermediates reaction free energies (ΔG), total spin multiplicity (**S_{tot}**), Mulliken spin populations (**SP**) for the neighbor metal **M**, the active site **M** and the evolving oxygen **O**, the **M-O** distance (**d_{M-O}**, in Å) and the Mayer bond order (**BO**) related to the product of each step for **(trans,cis)-NiNi**.

(trans,cis)-NiNi	ΔG (eV)	S_{tot}	SP Ni	SP Ni	SP O	d_{M-O}	BO
**M →MOH**	1.96	4	1.7	0.9	-0.1	1.836	0.8779
**MOH→MO**	2.17	5	1.7	0.8	1.0	1.783	0.9176

**MO→MOOH**	0.73	6	1.7	1.7	0.7	2.224	0.2643
**MOOH→M**	0.06	5	1.7	1.7	-	-	-

Table S16. Intermediates reaction free energies (ΔG), total spin multiplicity (**S**$_{tot}$), Mulliken spin populations (**SP**) for the neighbor metal **M**, the active site **M** and the evolving oxygen **O**, the **M-O** distance (**d**$_{M\text{-}O}$, in Å) and the Mayer bond order (**BO**) related to the product of each step for **(cis)-CoCo**.

(cis)-CoCo	ΔG (eV)	S$_{tot}$	SP Co	SP Co	SP O	d$_{M\text{-}O}$	BO
**M →MOH**	1.51	4	2.8	0.0	0.0	1.865	0.9163
**MOH→MO**	1.83	7	2.8	1.9	1.0	1.627	1.7071
**MO→MOOH**	1.44	4	2.8	0.0	0.0	1.879	0.807
**MOOH→M**	0.14	7	2.8	2.8	-	-	-

Table S17. Intermediates reaction free energies (ΔG), total spin multiplicity (**S**$_{tot}$), Mulliken spin populations (**SP**) for the neighbor metal **M**, the active site **M** and the evolving oxygen **O**, the **M-O** distance (**d**$_{M\text{-}O}$, in Å) and the Mayer bond order (**BO**) related to the product of each step for **(trans,cis)-NiFe**.

(trans,cis)-NiFe	ΔG (eV)	S$_{tot}$	SP Ni	SP Fe	SP O	d$_{M\text{-}O}$	BO
**M →MOH**	0.83	8	1.7	4.2	0.3	1.861	1.0176
**MOH→MO**	1.79	7	1.7	3.2	0.6	1.629	1.8295
**MO→MOOH**	1.73	8	1.7	4.2	0.4	1.903	0.928
**MOOH→M**	0.57	7	1.7	3.8	-	-	-

Table S18. Intermediates reaction free energies (ΔG), total spin multiplicity (S_{tot}), Mulliken spin populations (**SP**) for the neighbor metal **M**, the active site **M** and the evolving oxygen **O**, the **M-O** distance ($d_{M\text{-}O}$, in Å) and the Mayer bond order (**BO**) related to the product of each step for **(cis)-MnFe**.

(cis)-MnFe	ΔG (eV)	S_{tot}	SP Mn	SP Fe	SP O	$d_{M\text{-}O}$	BO
**M →MOH**	0.78	11	4.9	4.2	0.3	1.866	1.0059
**MOH→MO**	1.76	10	4.9	3.1	0.6	1.632	1.8213
**MO→MOOH**	1.82	11	4.9	4.2	0.3	1.915	0.8663
**MOOH→M**	0.55	10	4.9	3.8	-	-	-

Table S19. Intermediates reaction free energies (ΔG), total spin multiplicity (S_{tot}), Mulliken spin populations (**SP**) for the neighbor metal **M**, the active site **M** and the evolving oxygen **O**, the **M-O** distance ($d_{M\text{-}O}$, in Å) and the Mayer bond order (**BO**) related to the product of each step for **(trans,cis)-NiCo**.

(trans,cis)-NiCo	ΔG (eV)	S_{tot}	SP Ni	SP Co	SP O	$d_{M\text{-}O}$	BO
**M →MOH**	1.42	3	1.7	0.0	0.0	1.867	0.8702
**MOH→MO**	1.91	6	1.7	1.9	1.0	1.628	1.6994
**MO→MOOH**	1.43	3	1.7	0.0	0.0	1.872	0.8169
**MOOH→M**	0.16	6	1.7	2.8	-	-	-

Table S20. Intermediates reaction free energies (ΔG), total spin multiplicity (S_{tot}), Mulliken spin populations (**SP**) for the neighbor metal **M**, the active site **M** and the evolving oxygen **O**, the **M-O** distance ($d_{M\text{-}O}$, in Å) and the Mayer bond order (**BO**) related to the product of each step for **(trans,cis)-NiFe**. In order to study this reaction mechanism on the Nickel site, it was necessary to consider the

neighbor Iron as **M-OH**.

(trans,cis)-NiFe	ΔG (eV)	S_{tot}	SP Fe	SP Ni	SP O	$d_{M\text{-}O}$	BO
**M →MOH**	1.96	7	4.3	0.9	-0.1	1.835	0.8868
**MOH→MO**	2.18	8	4.2	0.8	1.0	1.785	0.9166
**MO→MOOH**	0.74	9	4.2	1.7	0.7	2.216	0.2661
**MOOH→M**	0.05	8	4.2	1.7	-	-	-

Table S21. Intermediates reaction free energies (ΔG), total spin multiplicity (S_{tot}), Mulliken spin populations (**SP**) for the neighbor metal **M**, the active site **M** and the evolving oxygen **O**, the **M-O** distance ($d_{M\text{-}O}$, in Å) and the Mayer bond order (**BO**) related to the product of each step for **(trans,cis)-NiMn**.

(trans,cis)-NiMn	ΔG (eV)	S_{tot}	SP Ni	SP Mn	SP O	$d_{M\text{-}O}$	BO
**M →MOH**	0.83	7	1.7	3.9	0.0	1.827	0.9811
**MOH→MO**	2.09	6	1.7	2.5	0.5	1.660	1.8763
**MO→MOOH**	1.39	7	1.7	4.0	-0.1	1.855	0.8685
**MOOH→M**	0.62	8	1.7	4.9	-	-	-

Table S22. Intermediates reaction free energies (ΔG), total spin multiplicity (S_{tot}), Mulliken spin populations (**SP**) for the neighbor metal **M**, the active site **M** and the evolving oxygen **O**, the **M-O** distance ($d_{M\text{-}O}$, in Å) and the Mayer bond order (**BO**) related to the product of each step for **(cis)-CoFe**.

(cis)-CoFe	ΔG (eV)	S_{tot}	SP Co	SP Fe	SP O	$d_{M\text{-}O}$	BO

**M →MOH**	0.93	9	2.8	4.2	0.3	1.868	0.9918
**MOH→MO**	1.74	8	2.8	3.2	0.6	1.632	1.8214
**MO→MOOH**	1.75	9	2.8	4.2	0.3	1.914	0.8711
**MOOH→M**	0.49	8	2.8	3.8	-	-	-

Table S23. Intermediates reaction free energies (ΔG), total spin multiplicity (S_{tot}), Mulliken spin populations (**SP**) for the neighbor metal **M**, the active site **M** and the evolving oxygen **O**, the **M-O** distance (d_{M-O}, in Å) and the Mayer bond order (**BO**) related to the product of each step for **(cis)-NiCo**.

(cis)-NiCo	ΔG (eV)	S_{tot}	SP Ni	SP Co	SP O	d_{M-O}	BO
**M →MOH**	1.36	6	1.7	0.0	0.0	1.874	0.8679
**MOH→MO**	1.92	9	1.7	1.9	1.0	1.628	1.7097
**MO→MOOH**	1.41	6	1.7	0.0	0.0	1.874	0.831
**MOOH→M**	0.23	9	1.7	2.8	-	-	-

Table S24. Intermediates reaction free energies (ΔG), total spin multiplicity (S_{tot}), Mulliken spin populations (**SP**) for the neighbor metal **M**, the active site **M** and the evolving oxygen **O**, the **M-O** distance (d_{M-O}, in Å) and the Mayer bond order (**BO**) related to the product of each step for **(trans,cis)-NiNi**. In this case, the spin configuration of the active site is kept constant along the reaction path.

(trans,cis)-NiNi	ΔG (eV)	S_{tot}	SP Ni(n)	SP Ni(as)	SP O	d_{M-O}	BO
**M →MOH**	2.50	6	1.7	1.9	0.7	1.851	1.0104
**MOH→MO**	1.62	7	1.7	1.8	1.7	1.764	1.1928

**MO→MOOH**	0.73	6	1.7	1.7	0.7	2.224	0.2643
**MOOH→M**	0.06	5	1.7	1.7	-	-	-

Table S25. Intermediates reaction free energies (ΔG), total spin multiplicity (**S_{tot}**), Mulliken spin populations (**SP**) for the neighbor metal **M**, the active site **M** and the evolving oxygen **O**, the **M-O** distance (**$d_{M\text{-}O}$**, in Å) and the Mayer bond order (**BO**) related to the product of each step for **(cis)-CoCo**. In this case, the spin configuration of the active site is kept constant along the reaction path.

**(cis)-CoCo**	**ΔG (eV)**	**S_{tot}**	**SP Co**	**SP Co**	**SP O**	**$d_{M\text{-}O}$**	**BO**
**M →MOH**	1.86	8	2.8	3.1	0.4	1.811	1.108
**MOH→MO**	2.14	9	2.8	3.0	1.4	1.748	1.4534
**MO→MOOH**	0.93	8	2.8	2.8	0.7	2.130	0.3805
**MOOH→M**	-0.01	7	2.8	2.8	-	-	-

S17 OER intermediates

Figure S31 shows the optimized structure of all OER intermediates for **(trans,cis)-NiNi**, here used as prototypical example.

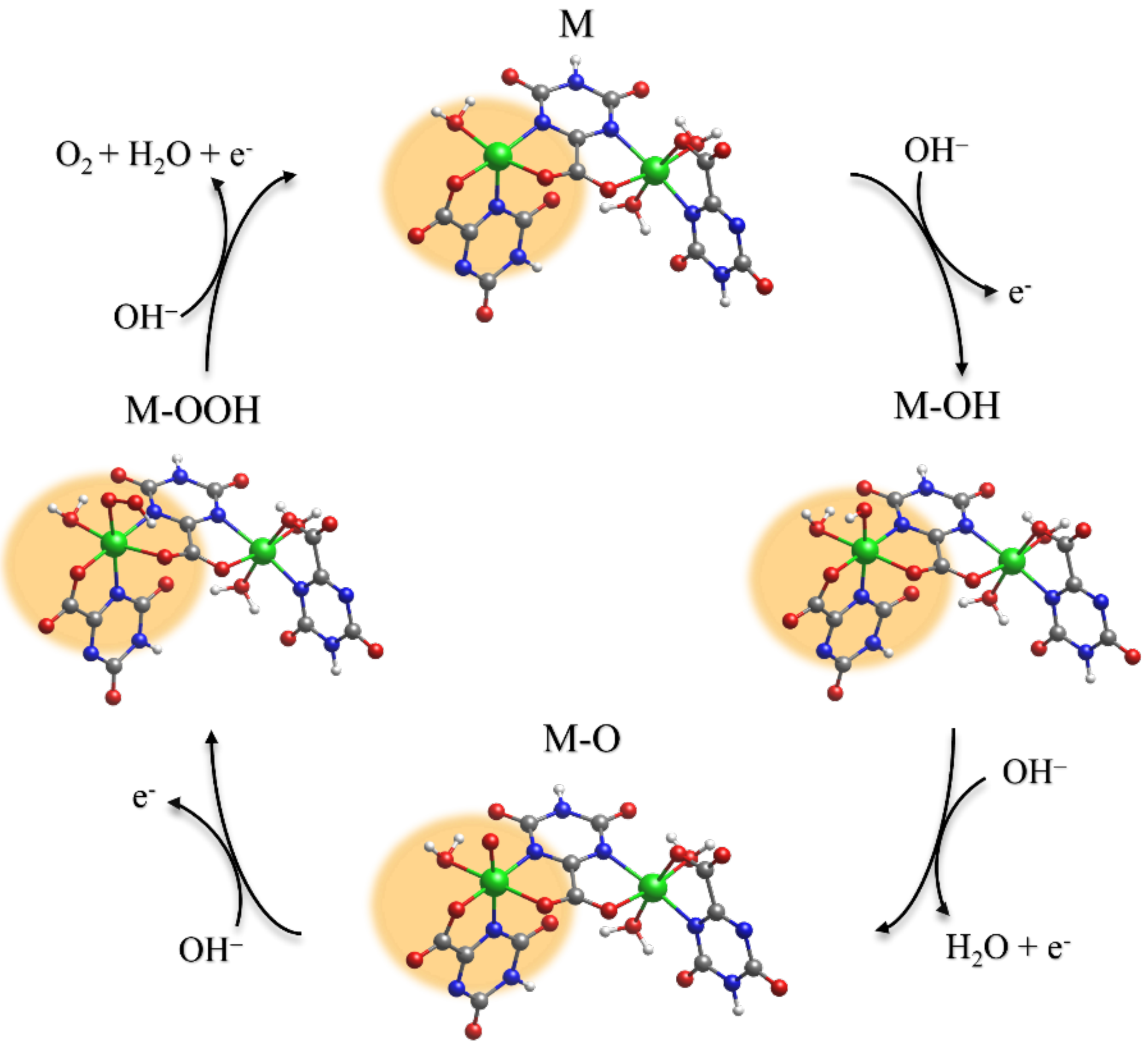

Figure S31. Optimized structures of **(trans,cis)-NiNi** intermediates.

S18 Structures

50

(cis,trans)-NiNi

Ni 8.9714498835 1.1521924412 3.6997178651

Ni	10.4125963864	-3.4611393607	6.3293631311
O	13.4067193620	-2.9040374283	7.7538098584
O	15.6672164001	-6.4039220888	5.9606625871
O	9.4805030509	-4.5835216599	7.9449216927
O	10.0682480502	-5.1177054549	5.1214917181
O	11.0517557181	-6.9421144014	4.2845682649
O	11.2879730336	4.3100121654	2.6647397720
O	13.2437735160	3.6885278829	7.1644386974
O	7.1768722460	-3.2609828289	7.4261570645
O	5.9969980137	0.5644596129	5.2968848714
O	10.8972253779	-1.9595010189	7.7862642991
O	11.0365873815	-2.2186973272	4.6910425393
O	10.3568846056	-0.4821958345	3.4567916404
O	7.8936751096	0.5799777357	1.8820935001
O	7.3712729169	2.5526568556	4.1135540533
O	9.9086968762	2.5564690485	2.5631763702
O	10.0328561467	0.4938017499	6.8695806050
N	14.5228954705	-4.6612657355	6.8545112245
N	13.4412920992	-6.2047387139	5.4515189304
N	12.2605934088	-4.3853384301	6.4249653513
N	12.0315781957	3.5707166228	5.2228572077
N	11.6375469951	2.0937892710	7.0035005498
N	10.2973071851	1.9445649910	5.1149928923
N	6.5641326176	-1.3926664585	6.2939812819
N	8.0187212811	-0.3854246618	4.7797400547
N	8.6401335954	-2.3752570622	5.8999564315

C	12.3677443932	-5.4821244258	5.6590929397
C	11.0580243518	-5.9051418393	4.9496572818
C	13.3804515326	-3.9228962531	7.0497633416
C	14.5994753561	-5.8085733415	6.0709314839
C	12.3593648715	3.1668083289	6.4919327321
C	10.6105600077	1.4557041691	6.3479033852
C	8.8444616315	-1.3777478935	5.0597086868
C	10.1984044864	-1.3587488427	4.3389401689
C	7.4548067207	-2.4038109374	6.5873211647
C	6.8184250404	-0.3429095032	5.4357684988
C	11.0523822640	2.9393150296	4.6221113816
C	10.7333153709	3.3294599524	3.1596448565
H	15.3688011783	-4.3426904480	7.3084696318
H	9.4299216435	-5.4961211640	7.6280720523
H	8.5629149929	-4.2262195939	7.8608841122
H	11.8838440788	1.7551444072	7.9243001845
H	5.6885061988	-1.3792592237	6.8018919556
H	10.7300471722	-1.0432380207	7.4790171091
H	11.8728932848	-2.0978136640	7.8430270259
H	7.8769409374	-0.3845573403	1.8037837340
H	8.4232127283	0.8884293332	1.1320228504
H	6.7011027400	1.9372215983	4.5005334379
H	7.0033724889	2.8560055969	3.2723331517

50

(cis)-CoCo

O	2.8999383728	-2.0539802491	7.3647724213
O	3.9660845927	-4.4472173276	14.3644249667
N	4.6221960339	-2.7463899625	13.0148169636
C	2.6381003381	-0.8818793254	7.6403579222
O	5.2905737734	-1.0618783187	11.6429231831
C	4.1793715769	-3.2426638970	14.2337579940
O	2.4782629805	-0.4022042454	8.8113937130
N	2.6898921151	-0.2718510289	5.2625820980
C	4.9005804501	-1.4173607256	12.7519783131
C	2.4799256026	0.1399474433	6.4897159979
O	2.7239605433	0.3899783090	3.0673815529
C	2.5447893783	0.6464358240	4.2544068100
N	4.0024359728	-2.3291941850	15.2407262308
O	5.2254751925	2.1925425447	7.7414968845
H	-0.3561589280	0.5604241794	8.2302563479
C	5.1837500655	2.1315367009	8.9597751601
N	4.7087866378	-0.5642238223	13.8072034433
N	2.1297174095	1.3726151381	6.8874598666
N	6.3421684997	2.2190694997	9.7216009032
H	7.2170577110	2.3217028070	9.2236994351
N	4.0226242517	1.9730232989	9.6842282220
H	-0.4506513419	0.9046049545	9.7295754435
C	4.2723253654	-1.0731487368	14.9643203354
Co	2.0383243132	1.6185956196	8.9731156961
O	-0.1340794295	1.2426223109	8.8796140549
C	6.3993276434	2.1556341697	11.0943619107

C	4.1119337073	1.9292813733	10.9984922024
O	1.7507936410	1.6652656565	11.0657939531
N	2.1704317729	1.9316394469	4.6361893601
O	7.4873457387	2.2115525612	11.6774166463
N	5.2021367650	2.0277761056	11.7429146953
C	2.7976263512	1.7273099491	11.7563272823
H	7.3517358820	1.7429908895	13.3111847500
C	1.9609074949	2.3280958116	5.9333425795
O	2.8689318074	1.6356827976	13.0015936115
H	2.0573328575	2.6258245362	3.9092232317
Co	4.8690770420	1.5331795821	13.8047635535
O	6.9818803777	1.4937881268	14.1973459581
C	4.0668956064	-0.0264242671	16.0841862531
O	3.6701177807	-0.4003295663	17.1883732301
O	1.6389730587	3.5008839760	6.1831314458
O	1.6159080400	3.7439221955	8.8228429620
O	4.3382078023	1.1785008379	15.7536405905
H	7.2960550418	0.5995076255	14.3874053308
H	0.7443071681	3.9452156178	9.1891013431
H	1.5253658827	3.8062375900	7.8333462445
O	4.8492547809	3.6746059988	14.2465621266
H	4.4035421299	4.1872345848	13.5574760065
H	4.3529173988	3.8475037096	15.0595037835
H	4.7537627515	-3.4055461143	12.2594069903

50

(cis)-CoFe

O	2.4879575518	-2.1754991830	7.3074048320
O	4.1030268950	-4.5175756491	14.4230980945
N	4.4130260206	-2.7846212212	12.9927721494
C	2.3518076544	-0.9827499309	7.5717777632
O	4.7181903784	-1.0658335163	11.5379602947
C	4.2755084741	-3.3092857361	14.2709207316
O	2.2058069846	-0.4861133403	8.7426036564
N	2.5832061974	-0.3712852133	5.2053421052
C	4.6213789074	-1.4491154766	12.7011762915
C	2.3545732059	0.0547571829	6.4259081526
O	2.8063546765	0.3024977792	3.0248511856
C	2.6050170265	0.5677853245	4.2069410409
N	4.3365584363	-2.4170570407	15.3099220693
O	5.1593676154	2.3003435663	7.7562328026
H	-0.5748846455	1.1456096894	7.9905136421
C	5.1347736419	2.2009785277	8.9721519037
N	4.7053441241	-0.6218795713	13.7905648916
N	2.1271771681	1.3120604019	6.8262670743
N	6.2968007352	2.3335108908	9.7221885101
H	7.1563829779	2.5082376681	9.2178703027
N	3.9907010530	1.9577660032	9.7032879748
H	-0.6412192661	0.7257380607	9.4691271766
C	4.5402552989	-1.1554212928	15.0071566734
Fe	1.9650867076	1.5343127797	9.0005178262
O	-0.2857730744	1.4034624897	8.8772117271

C	6.3769772737	2.2270395424	11.0909474763
C	4.0999144923	1.8769725836	11.0139186680
O	1.7406876481	1.5430156713	11.1278247284
N	2.3861029592	1.8876204444	4.5930008698
O	7.4649421957	2.3385575677	11.6643728719
N	5.1977626447	1.9963345766	11.7464734127
C	2.8002476351	1.6421042519	11.7953607040
H	7.2866568825	2.0875025189	14.8934904588
C	2.1481246370	2.2901339552	5.8833972266
O	2.8943586750	1.5814125125	13.0403873813
H	2.4048656661	2.5985289166	3.8736957152
Co	4.9188584472	1.4641803925	13.7802696643
O	7.0576548293	1.4911312346	14.1685847354
C	4.5755856056	-0.1315342695	16.1650149560
O	4.5293722472	-0.5395373876	17.3270514625
O	1.9678036544	3.4933276046	6.1387348032
O	1.5760221057	3.6677351809	8.7470182273
O	4.6449437622	1.0888490521	15.7988155264
H	7.3938456855	1.9036519620	13.3347035963
H	0.6438651678	3.8573461968	8.9219332343
H	1.6875602212	3.7391298553	7.7562911392
O	4.7503062632	3.5644485853	14.3504452456
H	4.0419568820	4.0176521138	13.8715899855
H	4.5038403401	3.6090639806	15.2858749607
H	4.3515403344	-3.4248482350	12.2125430779

50

(cis)-MnFe

O	2.2167216910	-1.9995217037	6.7389966296
O	4.6011315804	-4.6225340337	15.5459143906
N	4.9547948227	-3.1554971307	13.8520212032
C	2.1550014209	-0.8371223475	7.1337932022
O	5.3127461689	-1.6957466593	12.1434574296
C	4.6625263584	-3.4508066831	15.1777534840
O	2.0332962239	-0.4631737227	8.3526295696
N	2.4790308678	0.0114658942	4.8548062416
C	5.0507149296	-1.8790605232	13.3306748366
C	2.2307368335	0.3151644879	6.1073247872
O	2.7860500196	0.9022880038	2.7641891744
C	2.5651537490	1.0493665126	3.9633701526
N	4.4538307418	-2.3840766250	16.0141362230
O	5.0795387682	2.2424806428	7.5842829951
H	-0.6837119788	1.3444722709	7.8151517230
C	5.0694568664	2.0741351727	8.7924194260
N	4.8341315283	-0.8643965033	14.2230424291
N	2.0408431157	1.5308729895	6.6337976906
N	6.2445185609	2.1301895519	9.5307043490
H	7.1019950670	2.3108324288	9.0245885569
N	3.9298637955	1.8198308259	9.5273110137
H	-0.7449888942	0.7892179568	9.2487827496
C	4.5496337925	-1.1826719774	15.4916272037
Fe	1.8857628477	1.5351885425	8.8127957301

O	-0.3705593245	1.5051281168	8.7165819412
C	6.3364729502	1.9591112909	10.8910509594
C	4.0526823748	1.6722105823	10.8327716897
O	1.6890548804	1.3638047833	10.9374356175
N	2.3901230349	2.3292681685	4.4834327858
O	7.4360740589	2.0200142447	11.4510305064
N	5.1617323514	1.7260010742	11.5574554585
C	2.7484548739	1.4134222170	11.6081868885
H	7.2826723358	2.2786930160	14.5980833334
C	2.1325651502	2.6026100041	5.8035623306
O	2.8412978054	1.2755799783	12.8476466308
H	2.4589314600	3.1104472987	3.8445765844
Mn	4.8447610632	1.2775665374	13.7704193011
O	7.0577391603	1.5256391270	14.0352932210
C	4.3063095241	0.0322171641	16.4234436659
O	4.0769454744	-0.1710068132	17.6167453302
O	1.9971919879	3.7786610875	6.1826469506
O	1.5572516942	3.6954190149	8.7862992504
O	4.3654057254	1.1726621007	15.8528468656
H	7.3484793038	1.7671531406	13.1169878184
H	0.6249127259	3.8818511131	8.9638457373
H	1.6892102347	3.8677062224	7.8106375043
O	4.6407548011	3.5273081431	14.1792304001
H	4.1939480406	4.0124699942	13.4712160960
H	4.0993860170	3.6658286830	14.9695416886
H	5.1096434194	-3.9302136604	13.2209702537

50

(cis)-NiCo

O	2.1616294290	-2.0977672928	7.6829214164
O	4.6102983264	-4.5650179152	14.2344748563
N	4.7478832363	-2.7884475552	12.8308310611
C	2.1113565146	-0.8827922381	7.8845509926
O	4.8973658172	-1.0335197518	11.4002636012
C	4.6191120823	-3.3431508416	14.0954663026
O	1.9097392963	-0.3154711760	9.0083052897
N	2.6440632948	-0.4215044713	5.5317912354
C	4.7849599950	-1.4329273963	12.5557972177
C	2.3075883176	0.0840828942	6.6938065835
O	3.1589943408	0.1081527319	3.3622688662
C	2.8372806528	0.4547184670	4.4949687622
N	4.4991734600	-2.4627804589	15.1406044849
O	5.0754469379	1.9466094402	7.6654278368
H	-0.5385777236	1.2851892572	8.0227087124
C	5.0400443370	1.9282636114	8.8847611341
N	4.6873039591	-0.6120422142	13.6515082129
N	2.1159029041	1.3762226949	6.9932312400
N	6.2119363520	1.9421814750	9.6317854771
H	7.0842239874	1.9518204481	9.1191294206
N	3.8797837587	1.8926373443	9.6282636622
H	-0.6355205791	1.1875158732	9.5546731684
C	4.5360028514	-1.1819375898	14.8561515452

Ni	1.8834427840	1.7335440506	9.0027055105
O	-0.2709960429	1.7294462108	8.8400754561
C	6.2840253095	1.9274241412	11.0040437287
C	3.9858106108	1.8929857317	10.9425444678
O	1.6102890139	1.8440024336	11.0569983579
N	2.6517565305	1.8047026711	4.7801019154
O	7.3818581089	1.9149113370	11.5712283509
N	5.0894573095	1.9302199453	11.6704581275
C	2.6719509576	1.8306516886	11.7246832610
H	7.0679859244	2.4271950597	14.6912999680
C	2.3052162884	2.2986969982	6.0125018166
O	2.7579234382	1.7717755581	12.9726892103
H	2.7975189232	2.4695288835	4.0319131401
Co	4.7337068435	1.4954265507	13.7364058655
O	6.8499867434	1.6451790892	14.1667551085
C	4.3639351702	-0.1865187781	16.0323789462
O	4.2730206669	-0.6282771414	17.1782574105
O	2.1796662761	3.5214457578	6.1839884592
O	1.7508666331	3.8471457869	8.7799734232
O	4.3148878905	1.0452746237	15.6987508593
H	7.2211131429	1.7999748306	13.2598339630
H	0.8350136741	4.1094875737	8.9461136070
H	1.8702581164	3.8905148663	7.7921546510
O	4.5675759151	3.5878498207	14.3723976865
H	3.8286196642	4.0120631729	13.9132162986
H	4.3089186913	3.5661373181	15.3055851964

H	4.8204198686	-3.4203735166	12.0447641348

50

(trans,cis)-NiCo, Ni(trans) Co(cis)

Ni	10.4198556399	-3.4298581060	6.3844705915
Co	8.9063187476	1.1610609227	3.7545273986
O	10.1940451242	0.4974557188	6.8066931808
O	9.6889443817	2.7075263985	2.6209667592
O	7.2323771185	2.4577526203	4.1763906766
H	6.9101557998	2.9288039819	3.3967305518
H	6.5278019536	1.8247698454	4.4532107040
O	8.0612195059	0.5026437726	1.8635770589
H	8.3981106763	1.0749405005	1.1586039528
H	8.3536433829	-0.3919838138	1.6377702475
O	10.3505773420	-0.4511394801	3.5669832773
O	11.0498866641	-2.1701765361	4.8180249703
O	10.9161032471	-1.9749551112	7.8383221110
H	11.8741664853	-2.1757133176	7.9656686160
H	10.8317920819	-1.0577094309	7.5041789581
O	5.8632743937	0.3843207980	5.2002756269
O	7.1763547107	-3.3176921087	7.4684198055
N	8.6476262161	-2.3774129303	5.9842776510
N	7.9727886959	-0.4324118972	4.8139884062
N	6.5064729354	-1.5072100491	6.2738572003
H	5.6056206371	-1.5420891799	6.7344578404
N	10.3228241120	1.9942049920	5.0785690931

N	11.7923519784	2.1101815946	6.8721275351
H	12.1082898464	1.7512570711	7.7633816165
C	10.5675257263	3.4613034056	3.1564396299
C	11.0189848295	3.0216925811	4.5682734175
C	6.7371608778	-0.4591988496	5.4051238443
C	7.4396569053	-2.4597296265	6.6254007595
C	10.1992315904	-1.3289185920	4.4464314143
C	8.8323863358	-1.3841996225	5.1361393444
C	10.7289639464	1.4769882072	6.2731443985
O	13.3897856901	3.7191111529	6.9609303568
O	11.0728794156	4.4634058329	2.6485829323
N	12.0329138391	3.6490406766	5.1148351975
C	12.4619644024	3.2078116281	6.3403128667
O	11.0825337253	-6.6507668709	3.9979050114
O	10.0631769447	-4.9962259381	5.1034070856
O	9.5230409640	-4.5784727798	7.9508971622
H	8.5934262947	-4.2484910768	7.8860523113
H	9.4948120037	-5.4912495583	7.6316889788
O	15.6032785590	-6.4815132321	5.9877125405
O	13.3660515613	-3.0705753295	7.9738872678
N	12.2444349815	-4.3855501010	6.4637196471
N	13.4129864082	-6.1584817430	5.3931226334
N	14.4715922654	-4.7810815190	6.9745736047
H	15.3033620614	-4.5333561859	7.4940182257
C	14.5508026911	-5.8589938790	6.0980708683
C	13.3446341705	-4.0214364654	7.1796816480

C	11.0640442722	-5.7353272726	4.8224319584
C	12.3542471983	-5.4174547325	5.6141361574

50

(trans,cis)-NiCo, Ni(cis) Co(trans)

Co	10.4150284851	-3.4927608764	6.2431485153
Ni	8.9327532679	1.1539762977	3.7084995615
O	10.0214417934	0.4905070924	6.8684788580
O	9.8672988109	2.5141428063	2.5471565873
O	7.4270127441	2.5864755153	4.1729483786
H	7.0272399704	2.9063107737	3.3527744436
H	6.7475089389	2.0115429554	4.6042140779
O	7.7913655678	0.6299405349	1.9626681447
H	8.2902410692	0.9313069782	1.1891120795
H	7.7308414491	-0.3316888289	1.8701346234
O	10.2453070535	-0.4791430109	3.3983143793
O	10.9101706409	-2.2859049545	4.5394348450
O	10.8327483052	-1.9932164889	7.7077973675
H	11.8016221054	-2.1187526785	7.8488485048
H	10.6644466562	-1.0610257848	7.4512749918
O	6.0255524181	0.6807166794	5.4470346824
O	7.1631242211	-3.2177929693	7.4637720417
N	8.5869883243	-2.3649497477	5.8812643035
N	7.9800998087	-0.3374991950	4.8150287687
N	6.5758835276	-1.3030423537	6.4003146875
H	5.7289956668	-1.2631953068	6.9534128648

N	10.2983173844	1.9072685785	5.0874830838
N	11.6677365572	2.0504438333	6.9560719805
H	11.9185215931	1.7177342216	7.8777876210
C	10.7262646132	3.2691432712	3.1163533949
C	11.0651978786	2.8824893307	4.5751770639
C	6.8207809375	-0.2567187005	5.5353755323
C	7.4373888516	-2.3555901897	6.6264823978
C	10.0945264632	-1.3785898145	4.2581316279
C	8.7803380492	-1.3642189479	5.0441090706
C	10.6162158542	1.4288680033	6.3232278501
O	13.3152082112	3.6067604728	7.0736720989
O	11.2966049729	4.2303344762	2.6009109414
N	12.0668034084	3.4996380639	5.1543726757
C	12.4070156952	3.1000367436	6.4212431185
O	11.3131819794	-6.9001855712	4.1261601138
O	10.2204124390	-5.1501006277	4.9888031688
O	9.3947313564	-4.6853210749	7.7193900535
H	8.4919867203	-4.2779842416	7.7229455954
H	9.2878587192	-5.5753569902	7.3561839568
O	15.7728147785	-6.3527850453	6.1788958138
O	13.3339013975	-2.9256416186	7.8704235905
N	12.3129300971	-4.3776463096	6.4116226761
N	13.5873063900	-6.1735373649	5.5086579022
N	14.5354962391	-4.6510626689	7.0254804560
H	15.3378887602	-4.3362836991	7.5548476797
C	14.6897492905	-5.7761781687	6.2216258183

C	13.3740701841	-3.9262808708	7.1399033942
C	11.2429120033	-5.8960929084	4.8381654564
C	12.4926460993	-5.4641943130	5.6432557230

50

(trans,cis)-NiFe, Ni(trans) Fe(cis)

Ni	10.4183607142	-3.4401855292	6.3957092579
Fe	8.9389457001	1.1773569361	3.6649302819
O	10.1543637099	0.4598322748	6.7933113465
O	9.8248069361	2.7569192737	2.6479219899
O	7.1220193199	2.3867301541	3.9088391887
H	6.7012837187	2.6454639616	3.0782341118
H	6.4960592563	1.7711213267	4.3648481578
O	8.0860696695	0.4552739419	1.7378329027
H	8.0358475736	1.1509319410	1.0667128526
H	8.6820518420	-0.2138950316	1.3715649865
O	10.2669429945	-0.5423241286	3.4437511255
O	11.0110889757	-2.2017610093	4.7453953929
O	10.9413936862	-1.9518380031	7.8484775516
H	11.9079920242	-2.1340289674	7.9272275931
H	10.8195786710	-1.0412969986	7.5032444310
O	5.8900835715	0.4575732171	5.2866214787
O	7.2169785897	-3.2225201097	7.5740594597
N	8.6383058172	-2.3557341260	5.9976896484
N	7.9519623960	-0.4270740863	4.8030804756
N	6.5420696306	-1.4165482277	6.3786104826

H	5.6678095474	-1.4107320406	6.8889066874
N	10.3403446416	2.0038513641	5.1140954077
N	11.7880407713	2.0313220135	6.9308323424
H	12.0839900271	1.6350529223	7.8131306281
C	10.6274307076	3.5397412326	3.2592603569
C	11.0600717386	3.0401820342	4.6560755650
C	6.7532552746	-0.4038802475	5.4652643094
C	7.4626983410	-2.3901920317	6.7006746357
C	10.1484067616	-1.3768403545	4.3707643594
C	8.8060126709	-1.3887928810	5.1147325371
C	10.7181586761	1.4433939187	6.2973346781
O	13.4199339763	3.5999314404	7.0930844371
O	11.0707738193	4.6061281120	2.8341664518
N	12.0758676855	3.6311063305	5.2366242830
C	12.4863107893	3.1329901985	6.4475405819
O	11.0646337757	-6.7379842776	4.0759390761
O	10.0451960309	-5.0635224672	5.1491835635
O	9.5118806158	-4.5815261325	8.0138454419
H	8.5981349138	-4.2071702713	7.9622617728
H	9.4391436154	-5.4835113993	7.6713397009
O	15.6162471059	-6.4831753778	5.9684547221
O	13.4103198840	-3.0105703337	7.8794047867
N	12.2574781423	-4.3902793580	6.4513945798
N	13.4122249221	-6.1891496813	5.4111564649
N	14.4996063798	-4.7527811197	6.9192983951
H	15.3422751104	-4.4819243907	7.4088397390

C	14.5639342245	-5.8610454992	6.0804455617
C	13.3736748887	-3.9919041414	7.1244704689
C	11.0506111078	-5.7996725802	4.8740764829
C	12.3538048061	-5.4481475421	5.6317291842

50

(trans,cis)-NiFe, Ni(cis) Fe(trans)

Ni	8.9162381752	1.2128515001	3.7059462813
Fe	10.3763461573	-3.5073846897	6.2120582936
O	13.4578982105	-2.8293858646	7.6885009191
O	15.8198729237	-6.3926828932	6.1713709133
O	9.3730123346	-4.6271068149	7.9009872808
O	10.2293237839	-5.2381737140	5.1177255636
O	11.2196128582	-7.1427032710	4.4971604621
O	11.2714293653	4.3531581032	2.7037888196
O	13.3275316910	3.5638658942	7.1296310055
O	7.0830591741	-3.2786377694	7.3171163881
O	5.9767363460	0.6547912277	5.3571359346
O	10.9262904383	-2.0118138942	7.6364065940
O	10.9229989558	-2.2427161534	4.5053632386
O	10.2396353318	-0.4479972256	3.3600135884
O	7.7462076981	0.7257179127	1.9162413079
O	7.3784019928	2.6499460929	4.2222751067
O	9.8564586149	2.6289012428	2.5843630397
O	10.0427686949	0.4449872654	6.8315549863
N	14.6231099996	-4.6212287140	6.9301237741

N	13.5981037226	-6.2747340470	5.6153236311
N	12.3650736357	-4.4067124283	6.4301661053
N	12.0652063515	3.5236166726	5.2172362869
N	11.6850607620	2.0061935026	6.9666714008
N	10.2998752823	1.9319065420	5.1066647008
N	6.5083446353	-1.3501634710	6.2735746196
N	7.9600376409	-0.3320296190	4.7649924139
N	8.5482604873	-2.3809420134	5.7974151100
C	12.5095146825	-5.5529722634	5.7523625466
C	11.2207648771	-6.0467613130	5.0540496653
C	13.4636471921	-3.8933522873	7.0484657990
C	14.7366989544	-5.8151510147	6.2239700735
C	12.4162768263	3.0783050216	6.4660395602
C	10.6302316428	1.4068626965	6.3193955484
C	8.7616549172	-1.3593478713	4.9864134044
C	10.0936347996	-1.3518727135	4.2176273429
C	7.3739500246	-2.3985032896	6.5076990391
C	6.7753394288	-0.2789356781	5.4476681076
C	11.0604384584	2.9269899623	4.6232919761
C	10.7111354357	3.3675607143	3.1820481045
H	15.4532112997	-4.2596868798	7.3809515688
H	9.3410394452	-5.5678961202	7.6816205565
H	8.4550845448	-4.2897727231	7.7778397973
H	11.9444348738	1.6397821794	7.8731213361
H	5.6431752881	-1.3305442245	6.7988475989
H	10.7357411194	-1.0788823418	7.3921008043

H	11.9071389905	-2.1290086628	7.7227550916
H	7.7026939821	-0.2353886239	1.8094993775
H	8.2438565666	1.0460458352	1.1496381026
H	6.7001692714	2.0396263920	4.6035309101
H	6.9977499806	2.9962730228	3.4036463693

50

(trans,cis)-NiMn, Ni (trans) Mn (cis)

Ni	10.3996582320	-3.4433525339	6.4076797878
Mn	8.9199572537	1.2196910362	3.6348761531
O	10.1990919118	0.4841898663	6.7495739810
O	9.8202161887	2.8611132091	2.6244896897
O	7.0929532974	2.4066315521	4.1138677423
H	6.6704985329	2.8101594189	3.3438160471
H	6.4538134194	1.7403410379	4.4732527769
O	8.0657877488	0.6688548115	1.6073223118
H	8.4315168547	1.2232333289	0.9029255412
O	10.2377868727	-0.5352060209	3.4703170691
O	10.9747556760	-2.2128736222	4.7489258232
O	10.9293642646	-1.9310205886	7.8366235543
H	11.8952782784	-2.1180928728	7.9152539299
H	10.8173722982	-1.0252552494	7.4747876872
O	5.8045109953	0.3591846088	5.2507354996
O	7.1930532187	-3.2771337849	7.5714446280
N	8.6088095032	-2.3849161594	6.0040641515
N	7.8882810518	-0.4806866335	4.7891467651

N	6.4841170177	-1.4996339843	6.3530522245
H	5.6046372708	-1.5127682682	6.8542084469
N	10.3790580178	2.0448401688	5.0898741562
N	11.8629773963	2.0283790606	6.8765013871
H	12.1741700888	1.6182384918	7.7471174785
C	10.6428793567	3.6142607415	3.2467613390
C	11.0989819476	3.0779765994	4.6272301851
C	6.6839705843	-0.4848610046	5.4400962361
C	7.4271988864	-2.4452947847	6.6937258811
C	10.1107145059	-1.3860758789	4.3812059004
C	8.7607959534	-1.4196849771	5.1136402761
C	10.7722582066	1.4654916688	6.2577006547
O	13.5209574016	3.5706098770	7.0202441660
O	11.0943666985	4.6869243035	2.8448810515
N	12.1356478160	3.6473847040	5.1955285105
C	12.5659830356	3.1265587917	6.3892635777
O	11.0470713466	-6.7679206382	4.1250168246
O	10.0270249453	-5.0837185834	5.1823984664
O	9.5153199254	-4.5720152371	8.0485378111
H	8.5933950602	-4.2199606736	7.9838341627
H	9.4652163020	-5.4840154193	7.7295927012
O	15.6076286535	-6.4728576638	5.9885988337
O	13.4004873462	-2.9833339062	7.8671551485
N	12.2449023016	-4.3838625481	6.4619013918
N	13.3997276924	-6.1928887887	5.4394479380
N	14.4905880793	-4.7335082707	6.9226904768

H	15.3347571187	-4.4540834479	7.4047632004
C	14.5540005684	-5.8525289127	6.0980159238
C	13.3632179492	-3.9738712956	7.1241510873
C	11.0336484839	-5.8191740177	4.9108354598
C	12.3400650465	-5.4524407592	5.6560292129
H	8.2708393984	-0.2418267515	1.3513667506

50

(trans,cis)-NiMn, Ni (cis) Mn (trans)

Mn	10.3281976167	-3.6012028768	6.1979147876
Ni	8.9024877308	1.1937849695	3.7054947685
O	10.0063253095	0.4194220268	6.8359177609
O	9.8850302688	2.5311496157	2.5533365546
O	7.4806782446	2.6882879438	4.2258379065
H	7.0835740018	3.0469585148	3.4205129856
H	6.7800731909	2.1399399678	4.6586204357
O	7.7106686200	0.7491131341	1.9666227691
H	8.2106251172	1.0442299523	1.1913724692
O	10.1200435661	-0.4900439026	3.3341511527
O	10.7326531083	-2.3468650878	4.4207292432
O	10.8148295107	-2.0552365772	7.7169632988
H	11.7951588168	-2.1548651868	7.8060337308
H	10.6253096356	-1.1289230297	7.4522745514
O	6.0154441941	0.8349842497	5.4931398175
O	6.9911584043	-3.1464210217	7.4240682348
N	8.4268546892	-2.3463306019	5.8236339960

N	7.9034855056	-0.2698426595	4.8055905901
N	6.4829761193	-1.1888024447	6.4034152053
H	5.6510087063	-1.1156835246	6.9753431358
N	10.3158326519	1.8591784920	5.0788142797
N	11.7066407855	1.9195536905	6.9362980112
H	11.9553046596	1.5607131752	7.8487618349
C	10.7666951364	3.2508206866	3.1332522590
C	11.1082584724	2.8203915858	4.5794366814
C	6.7635765832	-0.1428894996	5.5505401608
C	7.2946935849	-2.2875322391	6.5935656715
C	9.9507679181	-1.4007296789	4.1791601843
C	8.6547267789	-1.3403237232	4.9997996438
C	10.6282816305	1.3467391894	6.3025254228
O	13.4057176684	3.4180027065	7.0684920794
O	11.3568609610	4.2103193767	2.6369888174
N	12.1346252466	3.3929034215	5.1611516427
C	12.4744866950	2.9554622344	6.4154399061
O	11.5518731029	-7.1101175681	4.2797562553
O	10.3375321800	-5.4084867156	5.0713386799
O	9.1418792051	-4.7277931379	7.7430232637
H	8.2640739232	-4.2705514631	7.7121653277
H	8.9864682611	-5.6280259398	7.4257879713
O	15.9823032784	-6.1714943427	6.2138408610
O	13.3748040710	-2.7991168882	7.7489520096
N	12.4119246120	-4.3889152965	6.3978014099
N	13.7787477386	-6.1605051232	5.5809304958

N	14.6607532902	-4.4960845318	6.9813748920
H	15.4504144167	-4.1046343646	7.4776531277
C	14.8680213814	-5.6562616757	6.2439249573
C	13.4590079987	-3.8370494756	7.0747569401
C	11.4143866754	-6.0787696934	4.9391603805
C	12.6449206491	-5.5104928725	5.6954105697
H	7.6191480872	-0.2087637906	1.8633928694

III. References

(1) Altomare, A.; Cuocci, C.; Giacovazzo, C.; Moliterni, A.; Rizzi, R.; Corriero, N.; Falcicchio, A. EXPO2013: A Kit of Tools for Phasing Crystal Structures from Powder Data. *J Appl Cryst* **2013**, *46* (4), 1231–1235. https://doi.org/10.1107/S0021889813013113.

(2) Toby, B. H.; Von Dreele, R. B. GSAS-II: The Genesis of a Modern Open-Source All Purpose Crystallography Software Package. *J Appl Cryst* **2013**, *46* (2), 544–549. https://doi.org/10.1107/S0021889813003531.

(3) de Wolff, P. M. The Definition of the Indexing Figure of Merit M20. *J Appl Cryst* **1972**, *5* (3), 243–243. https://doi.org/10.1107/S002188987200932X.

(4) Galli, S.; Tagliabue, G.; Masciocchi, N.; Wang, W. G.; Barea, E.; Navarro, J. A. R. From 1D Homoleptic to 2D Heteroleptic Pillared Coordination Polymers Containing Oxonato Bridges. *Inorganica Chimica Acta* **2011**, *371* (1), 79–87. https://doi.org/10.1016/j.ica.2011.03.008.

(5) McCrory, C. C. L.; Jung, S.; Peters, J. C.; Jaramillo, T. F. Benchmarking Heterogeneous Electrocatalysts for the Oxygen Evolution Reaction. *J. Am. Chem. Soc.* **2013**, *135* (45), 16977–16987. https://doi.org/10.1021/ja407115p.

(6) Neese, F. The ORCA Program System. *WIREs Computational Molecular Science* **2012**, *2* (1), 73–78. https://doi.org/10.1002/wcms.81.

(7) Becke, A. D. A New Mixing of Hartree–Fock and Local Density-functional Theories. *J. Chem. Phys.* **1993**, *98* (2), 1372–1377. https://doi.org/10.1063/1.464304.

(8) Lee, C.; Yang, W.; Parr, R. G. Development of the Colle-Salvetti Correlation-Energy Formula into a Functional of the Electron Density. *Phys. Rev. B* **1988**, *37* (2), 785–789. https://doi.org/10.1103/PhysRevB.37.785.

(9) Vosko, S. H.; Wilk, L.; Nusair, M. Accurate Spin-Dependent Electron Liquid Correlation Energies for Local Spin Density Calculations: A Critical Analysis. *Can. J. Phys.* **1980**, *58* (8), 1200–1211. https://doi.org/10.1139/p80-159.

(10) Stephens, P. J.; Devlin, F. J.; Chabalowski, C. F.; Frisch, M. J. Ab Initio Calculation of Vibrational Absorption and Circular Dichroism Spectra Using Density Functional Force Fields. *J. Phys. Chem.* **1994**, *98* (45), 11623–11627. https://doi.org/10.1021/j100096a001.

(11) Grimme, S.; Antony, J.; Ehrlich, S.; Krieg, H. A Consistent and Accurate *Ab Initio* Parametrization of Density Functional Dispersion Correction (DFT-D) for the 94 Elements H-Pu. *The Journal of Chemical Physics* **2010**, *132* (15), 154104. https://doi.org/10.1063/1.3382344.

(12) Grimme, S. Density Functional Theory with London Dispersion Corrections. *WIREs Comput Mol Sci* **2011**, *1* (2), 211–228. https://doi.org/10.1002/wcms.30.

(13) Weigend, F.; Ahlrichs, R. Balanced Basis Sets of Split Valence, Triple Zeta Valence and Quadruple Zeta Valence Quality for H to Rn: Design and Assessment of Accuracy. *Phys. Chem. Chem. Phys.* **2005**, *7* (18), 3297–3305. https://doi.org/10.1039/B508541A.

(14) Barone, V.; Cossi, M. Quantum Calculation of Molecular Energies and Energy Gradients in Solution by a Conductor Solvent Model. *J. Phys. Chem. A* **1998**, *102* (11), 1995–2001. https://doi.org/10.1021/jp9716997.

(15) Cossi, M.; Rega, N.; Scalmani, G.; Barone, V. Energies, Structures, and Electronic Properties of Molecules in Solution with the C-PCM Solvation Model. *J. Comput. Chem.* **2003**, *24* (6), 669–681. https://doi.org/10.1002/jcc.10189.

(16) Li, H.; Jensen, J. H. Partial Hessian Vibrational Analysis: The Localization of the Molecular Vibrational Energy and Entropy. *Theoretical Chemistry Accounts: Theory, Computation, and Modeling (Theoretica Chimica Acta)* **2002**, *107* (4), 211–219. https://doi.org/10.1007/s00214-001-0317-7.

(17) Liang, Q.; Brocks, G.; Bieberle-Hütter, A. Oxygen Evolution Reaction (OER) Mechanism under Alkaline and Acidic Conditions. *J. Phys. Energy* **2021**, *3* (2), 026001. https://doi.org/10.1088/2515-7655/abdc85.

(18) Man, I. C.; Su, H.; Calle-Vallejo, F.; Hansen, H. A.; Martínez, J. I.; Inoglu, N. G.; Kitchin, J.; Jaramillo, T. F.; Nørskov, J. K.; Rossmeisl, J. Universality in Oxygen Evolution Electrocatalysis on Oxide Surfaces. *ChemCatChem* **2011**, *3* (7), 1159–1165. https://doi.org/10.1002/cctc.201000397.

(19) Rossmeisl, J.; Qu, Z.-W.; Zhu, H.; Kroes, G.-J.; Nørskov, J. K. Electrolysis of Water on Oxide Surfaces. *Journal of Electroanalytical Chemistry* **2007**, *607* (1–2), 83–89. https://doi.org/10.1016/j.jelechem.2006.11.008.

(20) Nørskov, J. K.; Rossmeisl, J.; Logadottir, A.; Lindqvist, L.; Kitchin, J. R.; Bligaard, T.; Jónsson, H. Origin of the Overpotential for Oxygen Reduction at a Fuel-Cell Cathode. *J. Phys. Chem. B* **2004**, *108* (46), 17886–17892. https://doi.org/10.1021/jp047349j.
